# PsittaWel: A welfare assessment tool for companion parrots

**DOI:** 10.1017/awf.2026.10089

**Published:** 2026-05-14

**Authors:** Andrea Piseddu, Yvonne van Zeeland, Lauren Hemsworth, Jean-Loup Rault

**Affiliations:** 1 Animal Welfare Science Unit, Vetmeduni Vienna, Austria; 2 Division of Zoological Medicine, Department of Clinical Sciences, Faculty of Veterinary Medicine, Utrecht University, The Netherlands; 3 Animal Welfare Science Centre, Faculty of Science, The University of Melbourne, Parkville, VIC, Australia

**Keywords:** Animal welfare, caregiver, indicator, monitoring, pet, Psitacciformes, Psittacine, welfare protocol

## Abstract

While parrots are popular companion animals, many experience significant welfare challenges, often resulting from not fulfilling their behavioural, environmental, and social needs. To address this issue, we developed a welfare assessment tool designed specifically for companion parrot caregivers. We involved a panel of seven experts with extensive working experience with companion parrots and in animal welfare assessment. Using an iterative process consisting of four online surveys and nine focus group meetings, the expert panel was invited to select relevant welfare indicators, rephrase selected indicators into user-friendly questions, identify missing content, and finally review the wording and structure of the tool. A preliminary version of the tool was then evaluated by four additional anonymous external experts and 69 parrot caregivers for its completeness, clarity and practicality through an online survey. Feedback from this stage was incorporated, and the revised version was subsequently reviewed again by the initial expert panel. The resulting tool, PsittaWel (freely downloadable at: https://www.vetmeduni.ac.at/psittawel), consists of 75 questions covering 145 welfare indicators and is divided into eight sections covering key aspects of parrot welfare: general information; physical health; housing and physical activity; provision of enrichment and exploration; nutrition and maintenance behaviours; social and reproductive behaviours; parrot-human interactions; maladaptive and fear-related behaviours. Further research is needed to assess the reliability of the indicators. Nevertheless, PsittaWel is already a valuable tool for caregivers to monitor welfare and identify potential room for improvement, ultimately improving the lives of their companion parrot.

## Introduction

In the last decades, public concern for animal mental and physical health has significantly increased, leading to the urgency to develop approaches that provide objective assessment of animal welfare. In this regard, a large body of literature has focused on identifying and validating measurements to help recognise and mitigate suffering, while ensuring that animals under human care experience positive mental states (Rault *et al.* 2025) and are provided a life worth living (Farm Animal Welfare Council 2009; Green & Mellor 2011). These measurements are commonly referred to as welfare indicators and can be categorised into two groups: (1) animal-based indicators, i.e. behavioural, physical and physiological parameters that reflect how well an animal is coping with its environment; and (2) environment-based indicators, i.e. husbandry and management practices that inform about the appropriateness of the environment itself in preventing mental and physical suffering and promoting positive mental states and rewarding experiences (EFSA Panel on Animal Health Welfare 2012b; van der Staay *et al.* 2025). The animal- and environment-based welfare indicators selected for inclusion in the welfare assessment tool should be valid and cover the different physical welfare domains (i.e. nutrition, physical environment, health, behavioural interactions) in order for the tool to provide a comprehensive overview of an animal’s welfare state (Mellor *et al.* 2020). Moreover, the selected welfare indicators need to be measurable with reasonable effort, cost, time, and skill to make a welfare assessment tool practical in real-world settings (EFSA Panel on Animal Health and Welfare [AHAW] 2012a; Yon *et al.* 2019).

Welfare assessment tools have been developed for a wide range of animals kept in captivity, including in farm (Butterworth *et al.* 2009; Blokhuis *et al.* 2013; Wickens 2015; Courboulay *et al.* 2020; Vasdal *et al.* 2022; Dalmau *et al.* 2024), zoo (Yon *et al.* 2019; Holst & Miller-Morgan 2021; Maher *et al.* 2021; Narshi *et al.* 2022; Clegg *et al.* 2023; Dazord & Primault 2024; McGill *et al.* 2025) and laboratory settings (Spangenberg & Keeling 2016; Paterson *et al.* 2024). Despite growing concern for assessing the welfare of animals kept as companions, welfare assessment tools have thus far focused mostly on dogs and cats (Mullan & Main 2007; Freeman *et al.* 2016; Dawson *et al.* 2017, 2018; de Assis & Mills 2021; Malkani *et al.* 2022; Schmutz *et al.* 2022).

For parrots, despite their popularity (Kidd & Kidd 1998; Meyers 1998; Anderson 2003; Engebretson 2006) and millions of them being kept in private households and other captive conditions (e.g. zoos, shelters, reintroduction and breeding centres) (Mellor *et al.* 2021), science-based guidelines for assessing parrot welfare are still lacking. Due to parrots being highly demanding in terms of cognitive and social needs, and essentially non-domesticated, it can be difficult to fulfil their needs in a captive setting, with limited opportunities for species-typical behaviours (e.g. physical activity, including flying, foraging, exploration, social interactions), or an inappropriate environment (e.g. exposure to aversive stimuli or inappropriate parrot-caregiver interactions). This can lead to the development of a range of behavioural problems, including aggression (biting), incessant screaming, stereotypies or feather-damaging behaviour (Seibert 2005; Engebretson 2006; Welle & Luescher 2006; van Zeeland *et al.* 2009; Wilson 2022a,b; Piseddu *et al.* 2024). Moreover, caregivers of companion parrots often find such problems difficult or impossible to manage, which can result in the parrot being relinquished (Meehan & Mench 2006). Additionally, the challenge to meet their complex dietary requirements or inappropriate husbandry practices can lead to serious health problems, such as vitamin and mineral deficiencies, obesity and associated diseases such as atherosclerosis (Engebretson 2006; Matson & Koutsos 2006; Beaufrère 2013; Wissink-Argilaga & Pellett 2015; Burns 2024). In fact, a lack of knowledge among parrot caregivers has been listed as a key factor contributing to compromised welfare (Chalmers *et al.* 2024).

Given the popularity of parrots as companion animals and the many welfare challenges that they face, a welfare assessment tool tailored for parrot caregivers is needed. Such a tool could help caregivers to: (1) evaluate the welfare state of their parrots by observing their behaviour and physical condition; (2) evaluate the appropriateness of the provided diet, living environment and social interactions; and (3) evaluate whether important species-typical needs are fulfilled. Additionally, this tool could be useful to monitor welfare over time, and aid in resource prioritisation (i.e. where are husbandry or dietary adjustments most urgently needed; where should focus of research be directed towards; Jones *et al.* 2022; Rowe & Mullan 2022), thereby eventually helping to unravel and reduce risks for health and behavioural problems and subsequent premature death or relinquishment through raising awareness and educating caregivers on parrots’ needs.

Similar to the methods employed in other studies (Yon *et al.* 2019; Truelove *et al.* 2020; Ghimire *et al.* 2024), we first identified potential welfare indicators for parrots through a systematic literature review (Piseddu *et al.* 2024). The identified welfare indicators were subsequently evaluated for their internal (i.e. the extent to which they reflect parrot welfare) and external validity (i.e. their applicability across all parrot species), as well as for their feasibility and importance through an online Delphi consultation survey involving experts in parrot behaviour, welfare and veterinary care (Piseddu *et al.* 2025). The present study aimed to select a sub-set of these previously identified parrot welfare indicators and incorporate them in a parrot welfare assessment tool that covers the essential aspects of parrot welfare and can be used by caregivers to regularly monitor the welfare of their companion parrots. To achieve this, a panel of parrot and animal welfare experts participated in iterative online surveys and focus group meetings with the objective of reaching consensus on: (1) which previously identified indicators to include in the tool based on their importance and feasibility for caregivers; (2) whether and what essential information is missing and should be added; (3) whether and what items are irrelevant or redundant and should be excluded from the final tool; and (4) how to phrase the questions on the selected indicators to ensure clarity for the caregiver(s). We then consulted additional external experts and parrot caregivers through an online survey to further refine the welfare assessment tool based on the feedback provided.

## Materials and methods

### Ethical considerations

The project was assessed by the Ethics Committee of the Medical University of Vienna, which determined that, in accordance with the European Medicines Agency’s Guideline for Good Clinical Practice and the Declaration of Helsinki (Kurihara *et al.* 2024), an ethical approval was not required for this study. All participants involved in the expert panel provided their written informed consent prior to participating in both the online surveys and the online meetings, including explicit written consent to be acknowledged by name. Participants in the final online surveys (caregivers and external parrot experts) provided their informed consent to participate, but conducted the survey anonymously, with no personal information required. All data were handled and stored in compliance with the European General Data Protection Regulation.

### Welfare assessment tool development steps

**Figure 1.**
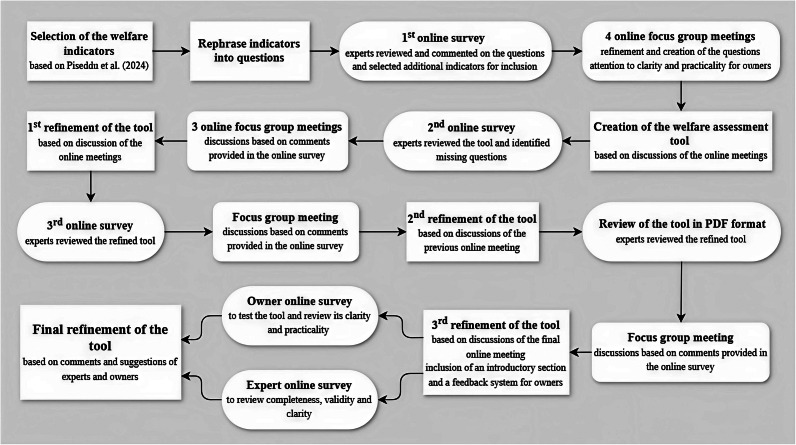
Flowchart illustrating the iterative process used to develop the parrot welfare assessment tool. The process involved four online surveys and nine focus group meetings with a panel of parrot experts, as well as an online survey with parrot caregivers and one with parrot experts external to the panel. The feedback and insights gathered through surveys and focus group meetings were used to refine and improve the assessment tool over multiple iterations.

The development of the parrot welfare assessment tool consisted of three main steps: (1) the selection of welfare indicators; (2) the creation and refinements of the tool through an iteration of online surveys and focus group meetings with a panel of parrot experts; and (3) the consultation of anonymous experts and parrot caregivers to improve tool’s completeness, validity, clarity and practicality. All the steps are described in detail in the following paragraphs and summarised in Figure 1.

### Selection of parrot welfare indicators

One hundred and forty-two welfare indicators were selected from the results of previous studies to identify valid and feasible welfare indicators across extant parrot species through a systemic literature review (Piseddu *et al.* 2024) and Delphi consultation survey (Piseddu *et al.* 2025). These indicators included several animal- (i.e. behaviours and body measurements reflective of parrot welfare) and environment-based indicators (i.e. husbandry, nutritional and managemental practices impacting parrot welfare). Of the 142 indicators, 31 were preselected by the investigators who developed questions related to these indicators to be presented to the expert panel for evaluation prior to including these in the tool. The remaining 111 indicators were presented to the experts to be assessed for their potential inclusion in the tool.

### Welfare indicators preselected for inclusion in the tool

For the preselection of welfare indicators, we focused on the animal-based indicators that ranked in the top 20 of most-important animal-based indicators and which reached at least 65% of expert agreement for being valid for all or most parrot species (Piseddu *et al.* 2025). From this list, we preselected the 14 animal-based indicators that received at least 65% agreement among experts for being feasible to be assessed by caregivers (Table S1; Supplementary material). Additionally, we preselected the top 10 environment-based indicators, all of which reached at least 65% agreement for having high relevance for parrot welfare (Table S1; Supplementary material). We also preselected seven other indicators from the list that failed to meet our preselection criteria yet were similar or covered the same (biological) construct as six indicators that passed the preselection criteria, thereby allowing these indicators to be combined into one single item (Table S1; Supplementary material). For example, the question reflecting the indicator *expression of escape and fear-related behaviours* was integrated with the indicators *tremors*, *hiding* and *freezing* in order to capture different behavioural manifestations of fear in one item. Using these 31 preselected welfare indicators, the investigators developed 24 multiple-choice questions, of which six questions combined multiple indicators. As the welfare assessment tool aimed to identify and select indicators of both compromised and good welfare states, answer options for the questions were structured to reflect varying levels of welfare, ranging from compromised to good in the case of animal-based indicators and ranging from high risk for compromised welfare to high likelihood for good welfare for the environment-based indicators (see PsittaWel; freely downloadable at: https://www.vetmeduni.ac.at/psittawel).

### Welfare indicators presented to the experts for potential inclusion in the tool

One-hundred and eleven welfare indicators identified in our previous study (Piseddu *et al.* 2025) failed to meet the preselection criteria. These included 61 animal-based indicators, of which six ranking in the top 20 that failed to reach 65% agreement for feasibility and 55 that reached 65% agreement for being valid but not ranked in the top 20, and 50 environment-based indicators that reached at least 65% agreement for having high relevance for parrot welfare but not ranking in the top 10 of most important criteria (Table S2; Supplementary material). Although these indicators did not meet the preselection criteria, they were still presented to the expert panel with the aim of: (1) being re-evaluated for their feasibility and importance; and therefore; (2) to be included in the assessment tool through clear questions that accurately reflected the welfare construct they represented.

### Online surveys and focus group meetings

#### Expert recruitment

For this project, experts were recruited using a purposive sampling technique (Campbell *et al.* 2020). We targeted veterinarians and behavioural consultants with extensive working experience with companion parrots, as well as senior researchers with expertise related to parrot welfare or animal welfare assessment. Potential participants were identified through the investigators’ professional networks as well as through their recognised expertise in the field. No geographical restrictions were applied, and fluency in English was the only requirement. In line with guidelines for conducting focus group meetings, we aimed to include between six and ten participants (Krueger & Casey 2000; Rabiee 2007). Experts were invited to participate via email, with a brief explanation of the project aims and their potential role in shaping the development of the welfare assessment tool. To reach the desired group size, invitations were sent in stages rather than simultaneously to all potential participants: additional experts were contacted when initially invited experts declined to participate. In total, 14 experts were approached.

#### Protocol description

To develop the welfare assessment tool, we employed a mixed-method approach that combined expert consultation through four online surveys and nine focus group meetings using an iterative manner (Figure 1). The online surveys, created and administered using ‘LimeSurvey’ (Limesurvey GmbH), were used to gather comments and suggestions from experts in a structured format, ensuring that their first feedback was not influenced by other participants (Dror *et al.* 2018). These responses were then systematically organised and used as a basis for further discussion during the focus group meetings. This process allowed us to collect and refine expert feedback at multiple stages during the development of the tool. This approach also ensured that experts who failed to attend a focus group could still provide input, which was subsequently incorporated into the discussions.

The online meetings were conducted via the platform ‘Cisco Webex’ (Cisco Systems 2025) and lasted approximately 2 h, in line with recommendations from the literature to ensure optimal participant engagement (Rabiee 2007). The discussions were moderated by the investigators (AP, YvZ, LH, JLR) who ensured that all experts could express their opinion and every perspective was considered. As part of their role as moderators, the investigators guided the discussions by proposing new definitions, summarising findings, suggesting additional viewpoints and prompting further elaboration when appropriate. Time allocated for discussing a welfare indicator depended mostly on the number of suggestions and feedback received. A discussion was considered concluded when all experts expressed satisfaction with the outcome and summary statement.

#### First round of online survey and focus group meetings

In preparation for the focus group meetings, in June 2024 the experts were invited to fill out the first online survey, which needed to be completed no later than prior to the fourth focus group meeting held December 2024 (the full surveys in all languages can be found in OnlineSurveys as Supplementary material). The survey was divided into five sections, each containing a specific subset of indicators that were scheduled to be discussed in the upcoming online focus group meetings (Table S3; Supplementary material). This division was based on the biological constructs or the husbandry and management practices that the indicators reflected: nutrition, maintenance behaviours, body measurements; maladaptive and fear-related behaviours, body displays; housing and enrichment, locomotor and exploratory behaviours; parrot-human interactions and human-directed behaviours; social and reproductive behaviours. In each section of the survey, experts needed to: (1) indicate whether they agreed or disagreed with the phrasing of the question, and answer options proposed for the 24 indicators that the investigators had selected for inclusion in the tool. In case of disagreement, comments and suggestions for improvement could be provided in a dedicated comment box; (2) review the lists of indicators that failed to reach the selection criteria and indicate whether and which of these were deemed important enough to include in the welfare assessment, and, for those considered relevant, how they would recommend rephrasing these into a question. The experts were informed in advance about the content to be discussed in the upcoming four online meetings and were invited to complete the corresponding sections of the online survey at least one week prior to each discussion. Reminders were sent throughout the period of the four meetings to encourage completion of the relevant sections. The comments and suggestions received in the online survey were collated into a comprehensive document by one of the researchers (AP) and subsequently sent to the experts one week before the online focus group meetings for their review and preparation. This also allowed experts who failed to complete the survey to review the feedback in advance and understand the topics that would be discussed during the meetings.

We conducted four online focus group meetings between July and December 2024 (Table S3; Supplementary material). The discussion of the results focused on: (1) improvement of question clarity for the 24 welfare indicators where experts commented on the suggested phrasing of the questions; (2) reaching consensus regarding the inclusion of additional welfare indicators that were considered to be important to be included in the tool by one or more experts; and (3) how to construct appropriate questions and answer options for the additional welfare indicators on which consensus was reached regarding their inclusion in the tool. During each focus group meeting, the moderators briefly summarised the survey findings for each indicator, including the corresponding comments and suggestions, and then opened the discussion to allow each expert to provide input. Special attention was given to ensure that the questions created during the discussion captured the context-dependent validity of certain measurements as welfare indicators. For instance, for the indicator aggressive and fearful responses, the frequency in which these behaviours were expressed (i.e. daily, weekly, monthly) was deemed more appropriate in relation to welfare assessment than its simple occurrence (yes/no). Efforts were also made to ensure that each answer option accurately represented a clear gradient of welfare conditions from compromised to good (animal-based indicators) or of likelihood of experiencing compromised to good welfare (environment-based indicators). Additionally, care was taken to design questions and answer options that were meaningful for caregivers and encouraged them to take practical measurements or make objective observations, enhancing both the accuracy and educational value of the assessment.

#### Creation of the first draft of the welfare assessment tool

After completion of the four online focus group meetings, based on experts’ input received, the investigators revised the 24 existing questions with regard to important indicators, and included 44 new questions for the indicators that were deemed important by the experts to be included in the welfare tool. These 68 questions were then combined to create a first draft of the welfare assessment tool consisting of eight sections: general information, physical health, housing and physical activity, provision of enrichment and exploration, nutrition and maintenance behaviours, social and reproductive behaviours, parrot-human interactions, maladaptive and fear-related behaviours.

#### Second round of online survey and focus group meetings

The experts had the opportunity to review the first draft of the welfare assessment tool through an online survey (see Supplementary material). Specifically, they were asked to review the indicators and questions for which consensus was reached in the previous round mostly for textual revisions, while providing further comments and suggestions for the additional indicators that were added in at a later stage (as focus group meetings did not always allow for sufficient time to reach consensus on the exact phrasing of the question and answer options). At the end of each section, experts were also invited to indicate whether any essential information was missing and if any content was considered non-essential and could therefore potentially be removed. Additionally, experts were asked to indicate their preferred order of presenting the sections in the assessment tool.

The outcome of the second survey was subsequently discussed with the experts during a second series of three online focus group meetings held between February and March 2025, using a similar approach as for the first round to reach consensus on the suggested revisions from round two (Table S3; Supplementary material). Additionally, experts discussed which essential questions were missing in the tool and which existing questions could be removed if deemed not the most important for parrot welfare. Particular attention was also paid to refining wording in such a way as to ensure questions would be easy to understand for parrot caregivers.

#### First refinement of the welfare assessment tool

After the completion of the second round of online focus group meetings, based on the experts’ input, the investigators revised all existing questions for their content and clarity, questions on essential topics were added, irrelevant ones removed, and sections were reorganised in the order that was deemed most logical. In addition, some revisions were made by the investigators to improve content clarity.

#### Third round of online survey, second refinement and focus group meetings

Similar to the previous rounds, experts were asked to complete an online survey to review and provide comments and feedback on the updated version of the tool. The input received in the online survey was discussed on a focus group meeting held in April 2025 (Table S3; Supplementary material).

A second revised version was sent to the experts incorporating both the refinements discussed during the focus group meetings and some additional revisions made independently by the authors (in Supplementary material). Experts were then given a final opportunity to provide input on the tool that was sent back to the primary researcher (AP). These final comments were subsequently discussed during a second online meeting held in May 2025 (Table S3; Supplementary material), aiming to reach consensus on the last remaining issues.

#### Third refinement of the welfare assessment tool

Following the third round of focus group meetings, the investigators made a second series of refinements to the tool based on the expert consensus reached during the meetings. In addition, an introductory section was developed to guide caregivers on how to accurately fill out and complete the assessment. To enhance its educational value, an instantaneous feedback feature using emojis was integrated into each question. The emojis were placed next to each answer option to enable caregivers to quickly understand whether the observed behaviours in general would be indicative of good or compromised welfare, and whether their husbandry and management practices were more likely to promote good welfare vs increase the risk of welfare concerns. Additionally, a legend explaining how to interpret the different emojis was placed at the beginning of each section.

### Consultation with external experts and caregivers

#### External expert survey

To ensure that no relevant aspect was overlooked and that the content of the tool was clearly formulated and considered valid, the last version of the welfare assessment tool was submitted for review to nine parrot experts who had not participated in the earlier discussions. These experts were selected based on their working experience with parrots and were invited to anonymously evaluate the tool through an online survey (see Supplementary material). At the end of each section, participants were able to fill out comment boxes to flag information that they considered inaccurate, unclear, or difficult to interpret (Table S4; Supplementary material). After completing their review of the tool, they were subsequently asked to provide their country of residence and share their impressions on the tool’s length, relevant yet missing content, and whether they would consider using the tool to support their professional interactions with parrot caregivers (Table S4; Supplementary material). The external experts had two weeks to complete the online survey.

#### Caregiver survey

To ensure that questions were clearly phrased and measurements were practical for everyday use, the final version of the welfare assessment tool was shared as an online survey accessible via a link for two weeks with parrot caregivers through the client networks of the experts who participated in the focus group meetings (survey available in Supplementary material). These clients were subsequently invited to review this version of the tool and use it to assess the welfare of their companion parrots. Similar to the external experts, at the end of each section, respondents fill out comment boxes to indicate any questions that they found unclear or difficult to interpret, as well as any behaviours or types of information that they found challenging to observe or collect in order to provide a response (Table S4; Supplementary material). Additionally, after completing the assessment, caregivers were asked similar questions as the external experts in relation to their country of residence, their impressions on the tool’s length, missing content, and usefulness of the tool for regularly monitoring the welfare of their companion parrot(s) (Table S4; Supplementary material).

### Final refinement of the welfare assessment tool

Based on feedback received from external parrot experts and parrot caregivers, and on a review of the tool conducted by the authors, the welfare assessment tool underwent a third and final refinement, which involved rephrasing of any questions that were considered unclear, incorporating missing information, and removing measurements that were deemed impractical or potentially misleading. The final version of the welfare assessment tool was sent to the panel of initial experts for a final review, and small refinements were made according to the feedback received.

## Results

### Participants’ demographics

#### Expert panel

Eight professionals with diverse backgrounds accepted the invitation to join our expert panel. One expert participated only in the initial survey round and the first online meetings before withdrawing from the project. The remaining seven experts, bringing complementary and overlapping expertise in veterinary medicine, animal behaviour consultancy, and research in animal welfare (Table 1), contributed to the subsequent surveys and focus group discussions, although not all completed all surveys and participated in every meeting. Participation in the online surveys ranged from 2 to 7 experts, while attendance at the focus group meetings ranged from 2 to 6 participants across the nine sessions (Table S3; Supplementary material). Some experts unable to attend certain meetings still contributed by providing their input through the online surveys. All participants had the opportunity to review the refinements and provide feedback even when they could not attend the discussions.

**Table 1.** Table displaying the professional background of each expert who participated in the development of the parrot welfare assessment tool. Each expert was assigned a number between 1 and 7. The panel included behavioural consultants, veterinarians, and researchers, with some experts having expertise in more than one area. A checkmark (✓) indicates the corresponding expertise of each participant, while a dash (–) indicates the absence of that expertise

Expert	Behavioural consultant	Veterinarian	Researcher
1	✔	**–**	**–**
2	✔	**–**	**–**
3	✔	**–**	**–**
4	✔	**–**	**–**
5	**–**	✔	✔
6	**–**	✔	✔
7	**✔**	✔	✔

#### Parrot caregivers and external experts

A total of 69 parrot caregivers participated in the online survey. Of these, 41 (59.4%) completed the survey in full, while the remaining 28 (40.6%) provided partial responses. Participants included caregivers of 27 parrot species, with grey parrots (*Psittacus Erithacus*; n = 13) being the most commonly kept by participants, followed by cockatiels (*Nymphicus hollandicus*; n = 9) and Eclectus parrots (*Eclectus roratus*; n = 5) (for the full list, see Table S5; Supplementary material). Forty-nine participants did not indicate their country of residence; the remaining 27 reported to live in the US (n = 11), Germany (n = 8), Italy (n = 7) and the Netherlands (n = 1).

Four external experts took part in the online survey, with 2 completing it in full and 2 partially. Two experts were avian veterinarians, one was both a parrot behavioural consultant and a researcher, and one was a researcher in parrot welfare and cognition. One expert was from Italy, one from US; the remaining two did not indicate their country of residence.

### Welfare assessment tool characteristics

The finalised tool is presented as a fillable PDF form (freely downloadable at: https://www.vetmeduni.ac.at/psittawel) and comprised of 75 questions: six address parrots’ demographic information and 69 cover 145 welfare indicators (Tables S6 and S7; Supplementary material).

#### Questions characteristics

The indicators are presented in question formats that best reflected their relationship to welfare. Question types include single-choice, multiple-choice, and yes/no answers, while some are organised in tables to allow more structured responses. Sixty-one questions target a single animal- or environment-based indicator, while eight (those in table-format) address multiple indicators within specific categories (e.g. signs of illness or social, reproductive, and maladaptive behaviours; Tables S6 and S7; Supplementary material). Several questions, particularly those related to physical conditions or specific types of enrichment, utilised accompanying photographs or illustrations to help caregivers select the most appropriate response. Answer options are structured according to a gradient from good to compromised welfare for questions targeting animal-based indicators, and from a high risk of compromised welfare to high likelihood of experiencing good welfare for the environment-based indicators. For eight questions, such as those related to rearing history or social housing, a clear gradient from compromised to good could not be defined by the experts due to context-specific interpretations; nevertheless, these questions are kept in the tool as these were considered useful, possibly in consultation with professionals such as behavioural consultants or veterinarians, to better understand and improve the parrots’ welfare.

#### Welfare indicators initially selected for inclusion

Of the 24 important welfare indicators preselected by the investigators for inclusion, 22 were retained and addressed within 21 questions of the assessment tool, either through dedicated items or within specific tables, with two of these questions presented in table format integrating additional indicators suggested by the expert panel (Tables S6 and S7; Supplementary material). The remaining three indicators, i.e. feather-damaging behaviour, daily food intake and amount of time spent sleeping, were considered by the panel of experts difficult or impractical to be assessed by caregivers due to the challenges in obtaining accurate measurements of these indicators. For this reason, these aspects were replaced with more practical and meaningful alternatives based on the literature, i.e. plumage condition score (adapted from Mellor *et al.* 2022) as an indirect measure of feather-damaging behaviour or, as proposed by the experts, changes in food and water consumptions and changes in resting/sleeping patterns as more practical measurement of daily food intake and amount of time spent sleeping (Table S8; Supplementary material).

#### Welfare indicators selected by the expert panel for inclusion

The experts selected and consented to include an additional 25 questions addressing indicators from the list that had not met the initial inclusion. Of these 25 questions, seven (table-format) are integrated with new welfare indicators proposed by the panel and that were missing from the list (Tables S6 and S7; Supplementary material). The experts’ input also resulted in an additional 23 questions addressing seven animal- and 16 environment-based indicators that were not part of the original proposed lists (Tables S6 and S7; Supplementary material).

#### Sections of the welfare assessment tool

**Figure 2.**
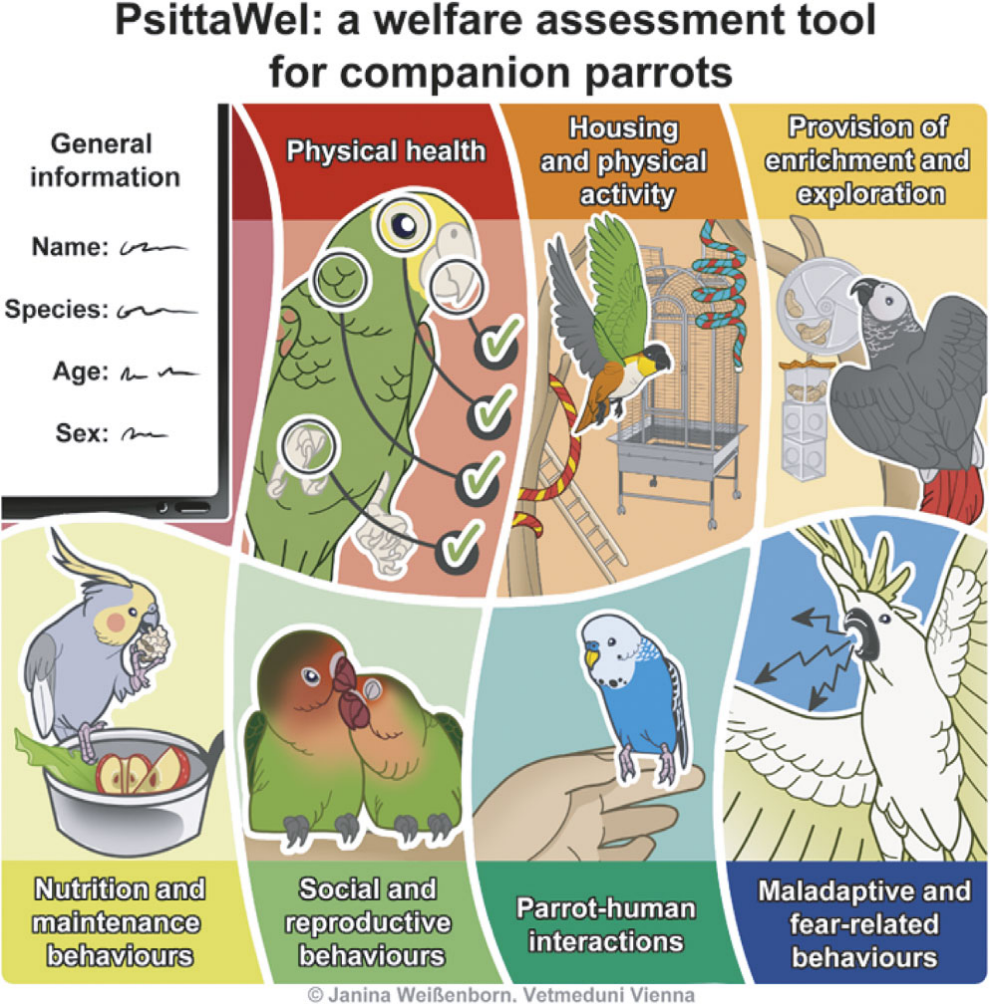
Overview of the eight sections included in the Parrot Welfare Assessment Tool (PsittaWel): general information, physical health, housing and physical activity, provision of enrichment and exploration, nutrition and maintenance behaviours, social and reproductive behaviours, parrot–human interactions, and maladaptive and fear-related behaviours.

The finalised tool comprises an introduction and eight sections: general information; physical health; housing and physical activity; provision of enrichment and exploration; nutrition and maintenance behaviours; social and reproductive behaviours; parrot-human interactions; maladaptive and fear-related behaviours (Figure 2).

### General introduction to the tool

The introduction is intended to explain to caregivers the purpose and proper use of the tool. It states that the welfare assessment is designed specifically for parrots (order *Psittaciformes*) kept as companions and aims to help caregivers evaluate aspects of their parrot’s physical condition, behaviour, and daily care. The introduction outlines how the questionnaire is structured and advises respondents to base their answers on recent observations, ideally from the past month, with some questions requiring direct interaction with the parrot. It also clarifies that the tool is not suitable for assessing welfare of chicks or breeding parrots, as these groups have different needs and require partly distinct welfare indicators. Importantly, the introduction encourages caregivers to seek support from qualified professionals, such as avian veterinarians or parrot behaviour consultants, if the outcome of the questionnaire points out any health or welfare concerns, underscoring the importance of expert guidance when interpreting and addressing potential issues.

#### Section 1: General information

This section includes 10 questions, of which four focus on the parrot’s demographics (name, species, age, and sex), three on rearing history and acquisition, and – as per expert panel recommendation – three on daily monitoring and veterinary check-ups.

#### Section 2: Physical health

This section consists of seven questions addressing aspects of the parrot’s physical health, including plumage condition, number and appearance of the parrot’s droppings, and pectoral muscle condition (indicative of being over- or underweight). Additionally, a list of signs of illness is provided to allow caregivers to specify whether this condition is present and/or diagnosed by a veterinarian and if the parrot is receiving any treatment for it. Moreover, one question specifically inquires whether the parrot has been previously diagnosed with disease (as the list only contains a limited number of signs of illness, rather than an exhaustive list of conditions/illnesses).

#### Section 3: Housing and physical activity

This section consists of 23 questions of which 17 cover aspects related to the parrot’s housing conditions (e.g. enclosure characteristics, provision of a retreat area, physical enrichment such as perches, branches, ladders etc, hygiene practices, climate, and time spent outside the enclosure), while the remaining five questions focus on the parrot’s daily (physical) activity, including opportunities and ability to fly.

#### Section 4: Provision of enrichment and exploration

This section includes eight questions, of which four focus on various aspects of environmental enrichment, both in terms of enrichment provided (types, frequency of provision and replacement, selection based on preferences) as well as use of the provided enrichment by the parrot (e.g. duration, frequency and type of interaction, exploratory behaviours, responses to novel objects).

#### Section 5: Nutrition and maintenance behaviours

This section consists of 11 questions, of which five focus on nutrition and feeding behaviour, including types and ratios of food provided, caregiver awareness of the appropriateness of the diet, frequency of food/water renewal, selective feeding behaviour by the parrot and (recent) changes in water and food consumption. The remaining six questions target maintenance behaviours, including beak maintenance behaviours, preening, bathing (opportunities and use thereof) and sleeping/resting (daily schedule and changes therein).

#### Section 6: Social and reproductive behaviours

This section includes three questions covering the level and type of social interaction with other parrots (including positive and negative interactions), and the frequency and type of reproductive behaviours displayed, if any.

#### Section 7: Parrot-human interactions

This section includes 10 questions that assess the type of interactions occurring between parrots and humans, focusing on aspects such as (a) time spent in presence of humans, (b) training activities, (c) type and level of interaction and physical contact (including both appropriate and inappropriate types of interaction), (d) display of human-directed bonding and mating behaviours (including extent to which the caregiver allows these) and (e) behaviour and responses in vicinity of caregivers, familiar and unfamiliar people.

#### Section 8: Maladaptive and fear-related behaviours

The last section consists of three questions covering the expression of maladaptive behaviours, including fear-related behaviours, excessive vocalisations, stereotypic and sham behaviours, and others (biting of nails and toes, object feeding), inquiring about their frequency and context within which they occur, if observed. Feather-damaging behaviour was initially included in the tool as one of the 24 important welfare indicators. However, after discussion it was excluded as experts agreed that its detection does not require direct observation of the behaviour itself, as it can be reliably inferred from the assessment of plumage condition already included in *Section 2.*

### Assessment of feedback system by external experts and caregivers

Comments received from both caregivers and external experts indicated that the instantaneous feedback system with emojis was problematic. Some caregivers reported that the immediate feedback, particularly in the form of emojis, was a “turn-off” and negatively affected their motivation to complete the assessment. Moreover, experts commented that the instantaneous feedback could bias the caregivers’ answers. Hence, emojis were replaced with a set of coloured circles (for questions included in the section ‘general information’), of parrot icons (animal-based indicators), house and hand icons (environment-based indicators representing housing conditions and parrot-human interactions, respectively). The icons ranged from green (indicating good welfare or a high likelihood of experiencing it) to red (indicating compromised welfare or a high risk of experiencing it). For colour-blind accessibility, answers were organised such that the answers were arranged in order from most optimal to least desired, either from top to bottom or from left to right (in tables).

### Overall feedback on the welfare assessment tool

#### External experts

The external experts provided 19 comments that were analysed by the investigators to refine 13 questions and ensure their clarity, validity and practicality and completeness of the tool (Table S9; Supplementary material). Only one out of four experts provided feedback regarding the length of the assessment indicating that it was “*too long and felt tiring or overwhelming to complete*”. This expert was also the only one to answer the question about use of the tool with “*Yes, definitely, it would be a valuable resource to support and guide caregivers effectively*”. None of the experts indicated that any questions were missing from the tool.

#### Caregivers

The caregivers provided 156 comments that were analysed for their relevance by the investigators to refine 17 questions and ensure their clarity and practicality (Table S9; Supplementary material). Of the 34 caregivers who responded to the question about the assessment’s length, 22 (64.7%) selected “*a bit lengthy, but definitely useful and worth completing*,” accompanied by comments such as “*I learned a lot from it! Good, bad, what I can improve. Very nice*” and “*I liked the coverage of the different aspects of keeping a parrot, and the welfare of the parrot*”. Nine (26.5%) responded “*the length was appropriate and well-balanced*”, with one comment stating, “*this is a good survey*”; two (5.9%) responded “*it was too long and felt tiring or overwhelming to complete*”, adding remarks like “*I have some trouble understanding the questions in English*”; and one responded (2.9%) “*it felt a bit short, I expected more questions*”.

Of the 33 caregivers who responded regarding the use of the tool, 12 (36.4%) responded “*yes, to some extent, I might use it occasionally, depending on the situation*”, with one commenting that completing the assessment via Limesurvey was “*a bit inconvenient*” and that they would maybe use it “*as an app where answers can be saved and things can be checked off*”. Nine (27.2%) responded “*yes, definitely, it would be a valuable resource to keep track of my parrot’s welfare*”, and another six (18.2%) responded “*maybe, I would need more time or repeated use to decide*”, with one comment stating “*I am not sure the rating is clear. I’m not sure if my guy is getting a good attempt at what he needs or if I’m failing miserably. I felt pretty good, need to improve in areas, but am not sure how I compare to the amazing right goal for him*”. The remaining six (18.2%) answered “*probably not, I don’t think I would find it useful in my daily routine*” with comments such as: “*I would be more interested in participating in the survey if I received feedback about the care of my birds afterwards*”, and “*seems like it only needs to be done once*”.

#### Experts panel

All seven experts reviewed and approved the final version of the welfare assessment tool and provided feedback that led to minor refinements of seven questions to improve their clarity (Table S10; Supplementary material).

## Discussion

The aim of this project was to develop a parrot welfare assessment tool that covers the most important aspects of parrot welfare and that caregivers can use to regularly monitor the welfare of their companion parrots. Following nine online meetings with a panel of experts, amounting to approximately 18 h of discussion, and after incorporating feedback from parrot caregivers and anonymous parrot specialists, we created PsittaWel, a parrot welfare assessment tool consisting of eight sections. While the initial tool only included 24 questions addressing welfare indicators deemed important in a previous Delphi study (Piseddu *et al.* 2025), the number of questions was expanded to 75 based on consultation of the expert panel who deemed all of these questions to be essential to provide caregivers a comprehensive picture of their parrot’s welfare. The final welfare assessment, designed to be used at least on a monthly basis, is estimated to take approximately 40 min for caregivers to complete. While this represent a substantial time investment, the size of the assessment, including the breadth of topics covered, reflects the multifactorial nature of assessing welfare, especially in non-domesticated species like parrots that have complex needs to be fulfilled in captivity (Meehan & Mench 2006).

### Indicators

PsittaWel comprises both animal- and environment-based welfare indicators, covering a relatively larger number of animal-based indicators, as these are believed to more accurately reflect the animal’s current and true welfare state (Smulders *et al.* 2023). While environment-based indicators do not immediately reflect the animal’s well-being, these were considered essential to include because of their risk for compromised welfare. By informing on the appropriateness of husbandry and management practices provided, the tool can assist caregivers in preventing compromised welfare and promote positive mental states. Moreover, answer options are structured such that these reflect a gradient from compromised to good welfare state for animal-based indicators, and from conditions associated with a high risk for compromised welfare to those offering a high likelihood of good welfare for environment-based indicators. The tool is thus designed to both help caregivers recognise potential problems, and to highlight what could contribute to a better quality of life for parrots, as welfare is not simply about eliminating or reducing negative experiences, but also about enabling the animal to engage in rewarding activities (e.g. provision of and interaction with enrichment items, positive social interactions with other parrots and/or humans) (Rault *et al.* 2025).

Some welfare indicators, such as stereotypies and activity levels, were deemed unfeasible to be assessed reliably by caregivers, yet were among those that were most important in our previous Delphi consultation study (Piseddu *et al.* 2025). Nevertheless, during our focus group meetings, the expert panel recommended including these indicators to encourage caregivers to pay closer attention to these aspects of welfare even though they require more time, practice, or systematic approach as these are essential for gaining a comprehensive understanding of the parrot’s welfare. Alternatively, future studies should focus on developing more feasible assessment methods for these indicators for caregivers, which might not allow for an exact measurement of duration or frequency of a behaviour but still provide valid results. For example, the question on plumage condition included in our assessment tool was adapted from a simplified scoring system developed for parrot caregivers, which has been shown to be reliable (Mellor *et al.* 2022). Testing the feasibility and validity of the assessment tool by caregivers through real-time assessment warrant further investigation, as well as potential comparison of results obtained from simplified scoring systems. Furthermore, the intra- and inter-observer reliability of the welfare indicators remain to be assessed as these can be considered valid only if yielding consistent results across different observers and for a similar situation assessed multiple times by the same observer (Browning 2022). Moreover, it would be valuable to investigate whether training or repeated use of the tool can increase the reliability with which the indicators can be assessed, especially for those that may require a higher level of skills and expertise. Given the lack of studies investigating the reliability of the indicators, results from the welfare assessment should ideally be reviewed by a qualified professional, who can guide caregivers in correctly interpreting the findings and discuss possible actions.

### Feedback system

To support caregivers in interpreting the results, a feedback system was incorporated into the assessment that linked each answer option to a corresponding welfare state, or potential risk vs opportunity for compromised or good welfare. Initially, the feedback system was visually represented through emojis next to each answer option but based on feedback from both parrot caregivers and external experts the emojis were perceived as judgmental or emotionally charged. Hence, the emojis were replaced with coloured icons, ranging from green to red. In addition, we added veterinary and behavioural consultant icons to highlight signs of illness or behavioural problems, respectively, that require immediate or urgent professional consultation. As the immediate feedback could still be perceived as discouraging or off-putting, thereby negatively affecting the caregiver’s experience and tool engagement, displaying feedback at the end of the assessment could help to mitigate this effect as well as reduce social desirability bias in the selected answers (Bispo Júnior 2022). Offering the assessment tool in an electronic, dynamic format, such as an app, would therefore be a priority as this allows for more flexible, user-friendly implementation of such a feature in order to only show feedback after completing the assessment.

Several questions targeting animal-based indicators included the answer option “I don’t know”. For this option, a magnifying glass was displayed as feedback to encourage caregivers to spend more time or more closely observe the specific behaviour in question, and increasing their awareness of welfare-relevant behaviours that previously may have been ignored or overlooked. For some questions, the expert panel deemed it difficult to associate the answer options with a clear welfare gradient, as their interpretation is context-dependent. For instance, although parrot species commonly kept as companions are highly social and should not be kept alone, introducing a new parrot should not be automatically assumed to improve the parrot’s welfare (Welle & Luescher 2006) and requires careful observation and continued monitoring, preferably with guidance from a professional. Additionally, benefits of social housing depend on the parrot’s rearing history and temperament, with incompatibility increasing the risk of undesired stress and conflict, and compromised welfare (Welle & Luescher 2006). While hand-rearing and capture from the wild generally are associated with a higher likelihood of welfare issues (Piseddu *et al.* 2025), the expert panel emphasised that assessing the actual impact requires professional guidance to help determine how to best mitigate the long-term, irreversible consequences of early life circumstances. As such, the expert panel recommended retaining questions related to these topics since these offer valuable insights for professionals to gain a better understanding of the parrot’s welfare and ensure a tailored intervention plan for welfare improvement.

A deliberate choice was made to have the feedback system neither attempt to identify underlying causes for welfare concerns, nor provide recommendations for improvement, as these are highly dependent on the individual and context. Whether an animal experiences compromised or good welfare involves a highly complex process where a multitude of factors that can interact with one another can be involved, including personality of the individual, physical health, social dynamics, and environment (Reimert *et al.* 2023). Therefore, guidance from a qualified professional (e.g. avian veterinarian or certified parrot behaviour consultant) is strongly recommended to review the results and discuss potential concerns and improvements. Nevertheless, future studies could help develop a more structured feedback system that provides information on the underlying motivation for a particular welfare gradient of a given answer. The need for such a refinement was underscored by several caregivers who desired clarity regarding the reason for certain answers being associated with (an increased risk for) compromised welfare. The tool does not replace professional support (nor intends to do so), as this remains essential to ensure that any actions taken as a result of the assessment are appropriate, effective, and tailored to the individual needs of each parrot.

When designing the tool, we chose not to include a cumulative scoring system for several reasons: (1) assigning and standardising appropriate weights to each indicator, while essential to produce a meaningful score, is extremely difficult due to variations in welfare needs between individuals (Richter & Hintze 2019); (2) developing a standardised, meaningful and reliable weighted scoring system and scoring algorithms, such as the Welfare Quality® protocols developed for farm animals (Botreau *et al.* 2007), would require much more validation and testing; and (3) the tool is primarily designed to inform and guide parrot caregivers on specific aspects that require further attention or modification, rather than assigning an overall score.

During the feedback round, we received input from 69 caregivers representing 27 different parrot species across four countries. The diversity in species was particularly valuable given the wide range of parrot species kept as companions, while international feedback helped to account for potential cultural differences in parrot care and welfare perceptions. While this provided useful cross-country input, all participating countries were in Europe and North America, so the findings largely reflect Western parrot-keeping contexts. Although many caregivers perceived the assessment as lengthy, the majority of them found the time investment worthwhile, and appreciated the wide range of topics covered. Nevertheless, to enhance its usability among caregivers, future research could explore the possibility of developing an abbreviated version of the assessment that retains its validity while reducing completion time. When inquiring about the use of the assessment tool, nearly one-third of caregivers indicated that they would definitely use it to regularly monitor their parrot’s welfare. Some caregivers also reported learning new things about parrot welfare through completing the assessment. While this finding alludes to the potential for the tool to offer educational value, its effectiveness still needs to be assessed in future studies. Only a few caregivers indicated they did not consider it relevant to their routine caregiving practices, which mostly seemed to relate to the language barrier in understanding and answering the questions for non-native English speakers, emphasising the need of a multilingual tool; and the manner in which the survey was administered, with a website or app suggested to significantly increase user-friendliness and usability of the tool compared to an online survey platform as it would allow for: (1) storage of prior results, thereby increasing efficiency of subsequent assessments and reducing completion time, since many questions (such as those related to housing or nutrition) may not need to be re-assessed repeatedly; (2) tracking (welfare) changes and enabling benchmarking over time; and (3) incorporation of a more detailed and structured feedback system to better comprehend the results. Ultimately, an app or webpage could also assist in gathering anonymous data that could be used in future studies to identify factors posing a risk to or promoting welfare, hence furthering our understanding of parrot welfare.

## Animal welfare implications and conclusion

Parrots are popular companion animals, living in millions of households worldwide. Despite their popularity, they often face significant welfare challenges, largely due to their complex needs that are difficult to meet in captivity, and a common lack of knowledge among their caregivers. Together with an international panel of experts, we developed PsittaWel; a tool designed to guide caregivers to assess and monitor their parrots’ welfare state and current living environment. A list of questions covering animal- and environment-based indicators inform caregivers about potential areas that can be improved upon to enhance their parrots’ quality of life and promote responsible care. Many indicators require further validation to ensure their assessment is reliable and relevant across different users, species and settings. Moreover, implementation of the tool via an app or web platform would improve its usability, allowing caregivers to track progress over time and enhance user engagement. Addressing these aspects is important to maximise the tool’s effectiveness and impact both from a practical and an educational perspective. Nevertheless, as preliminary feedback indicated, PsittaWel is useable in its present form and can be freely downloaded via the following link: https://www.vetmeduni.ac.at/psittawel. We still recommend a thorough review of the results in conjunction with a qualified professional to help ensure correct interpretation and adequacy of any actions taken.

## Supporting information

10.1017/awf.2026.10089.sm001 Piseddu et al. supplementary material Piseddu et al. supplementary material

## Supplementary material

The supplementary material for this article can be found at http://doi.org/10.1017/awf.2026.10089.
