# PsittaWel: A welfare assessment tool for companion parrots – ERRATUM

**DOI:** 10.1017/awf.2026.10100

**Published:** 2026-10-07

**Authors:** Andrea Piseddu, Yvonne van Zeeland, Lauren Hemsworth, Jean-Loup Rault

The Psittawel assessment tool included in the supplementary material for this article was originally published under the incorrect copyright license. It has therefore been removed. The publisher apologises for this error.

## References

[r1] Piseddu A, van Zeeland Y, Hemsworth L, and Rault J-L 2026 PsittaWel: A welfare assessment tool for companion parrots. Animal Welfare 35: e34. 10.1017/awf.2026.10089 PMC13175777 42147779

