# Supplementary material for: PsittaWel: A welfare assessment tool for companion parrots
Source: Anim Welf. 2026 May 14;35:e34. doi: 10.1017/awf.2026.10089 (PMC13175777; doi:10.1017/awf.2026.10089)
Supplement: Piseddu et al. supplementary material [file S096272862610089Xsup001.zip › OnlineSurveys_Original.pdf]

Focus Group Project: Online Survey

Information and Consent

vetmeduni
University of Veterinary Medicine, Vienna

Utrecht University

Dear expert,
Thank you for taking part in this survey. Please read the information about the project that you previously received before filling out this survey.
The aim of this survey is to find a consensus on which parrot welfare indicators should be included in a welfare assessment tool that parrot owners can use and how to use them effectively in practice. The final tool will include only the most important welfare indicators (approximately 40-50) and its function it will be to educate and inform owners about the most important aspects of parrot welfare.

This survey is dived in 5 sections according to the topics that will be discussed in the 5 online meetings. In each section, please indicate if you agree with the scores of indicators that we already decided to include in the assessment tool and select which additional indicators you believe should be included in the tool. The final goal will be to have a maximum of 5 indicators for each welfare dimension (e.g. housing, enrichment, maintenance behaviours, body measurements).

This survey requires approximately 60 min to be completed.
You have the **opportunity to save partially finished surveys** by clicking on the top right button saying "Resume later" that you can find in all pages of the survey. To open the partially saved survey, click on the link to the survey that you received by email.

Please note that:

- Participation is voluntary and anonymous.
- You can withdraw at any moment of the study.
- You have to be at least 18 years old to participate.
- The results of this survey will be included in a PhD thesis and published in a scientific journal.
- Your name will not appear in any published documents related to this study, and your statements will remain anonymous.
- Your personal data will be treated in compliance with the European General Data Protection Regulation and will be stored only as long as needed for the purpose of this study.

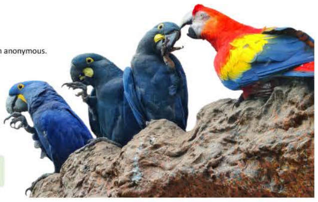

If you have any questions or concerns, please do not hesitate to contact the responsible person of this study:

Andrea Piseddu
Centre for animal Nutrition and Welfare, University of Veterinary Medicine Vienna, Vienna, Veterinaerplatz 1, 1210 Vienna
Andrea.Piseddu@vetmeduni.ac.at

By clicking the button "**Next**" and submitting your responses to the survey you are confirming to have read and understood the above statements and give your informed consent.

There are 49 questions in this survey.

Meeting A: Nutrition, maintenance behaviours, body measurements.

In this section you are presented with a list of animal-based and environment-based measures that will be discussed in the online meeting "nutrition, maintenance behaviours and body measurements".

Some of these indicators already passed the criteria for being included in the final tool. These indicators will be presented to owners as questions with multiple choice or yes/no answers. The answers to these questions map to a scoring system with 4 or 5 points per question, where 0 represents the worst husbandry, management, body condition or behaviour, and 3 or 4 the best one. For these indicators, please indicate if you would modify the questions and corresponding scoring systems.

All the remaining indicators did not pass the criteria but can still be included in the final tool. Please indicate which of them you would include in the tool and suggest how you would rephrase them as questions for the owners, including the answer options.

1 Nutrition

2 The environment-based indicators **"composition of the diet (quantity of fat, cholesterol, fibre, etc.)"**, **"frequency of fresh food provision"**, **"balance between provision of fresh and dried food"** will be combined and included in the assessment tool using the following scoring system:

Please select the option that best describe the diet of your parrot.
0: My parrot's diet consists entirely of seeds with no additional pellets, fresh fruits, or vegetables provided.
1: My parrot's diet consists of (extruded) pellets designed specifically for parrots, or of a mixture of pellets and seeds, with occasionally with some fresh fruits and/or vegetables provided in addition to this diet.
2: My parrot's diet consists primarily of (extruded) pellets designed specifically for parrots, supplemented daily with a moderate amount of fresh fruits, vegetables, seeds and nuts.
3: My parrot's diet consists primarily of (extruded) pellets designed specifically for parrots, supplemented daily with a variety of vegetables, a moderate amount of fresh fruits, and limited quantities of seeds and/or nuts.

Would you change this score?

If you would change the score, please explain in the comment box how you would change it.
Choose one of the following answers

- ○ I agree with the score as it is described.
- ○ I disagree with the current scores and would change these as indicated in the text box (please fill out the text box)
- ● No answer

Please enter your comment here:

3 **Husbandry and mangement conditions related to nutrition that did not pass the criteria for being considered among the most important parrot welfare indicators.**
Would you include any of the following measurements in the parrot welfare assessment tool?

YES - The indicator is valid for all parrot species and feasible for owners and I consider it to be one of the most important indicators of parrot welfare. I would include it in the tool.

MAYBE - The indicator is valid for all parrot species but is not feasible for owners and/or is not one of the most important indicators. I would include the indicator only in a different welfare assessment tool that considers more details.

NO - The measurement is not a valid indicator of welfare or it is valid only for some species. I would not include the measurements in a welfare assessment tool.

	Yes	Maybe	No	No answer
Variety of food items provided	○	○	○	●
Provision of supplements (multivitamin, calcium, essential fatty acids, etc.)	○	○	○	●
Frequency with which food is provided	○	○	○	●
Frequency of cleaning the food bowls	○	○	○	●
Consumption of human food	○	○	○	●
Time of day (morning, afternoon, evening) at which food is provided	○	○	○	●
Availability of clean fresh water	○	○	○	●
Location and number of feeding areas	○	○	○	●
Way in which food is stored	○	○	○	●
Pellet size	○	○	○	●

5 Maintenance behaviours

6 The animal-based indicators **"daily food intake", "daily water intake"** and **"body weight"** will be combined and included in the assessment tool using the following scoring system:

Which statement best describes your parrot's food and water intake?
N/A: I do not (regularly) check my parrot's food and water intake, nor weigh my bird
0: My parrot hardly eats or drinks or eats and drinks significantly less than what it normally does, and as a result has lost <10% of its body weight.
1: My parrot eats and drinks less than what it normally does, and mostly seems to eat its highly favored food items. As a result, my bird has lost 5-10% of its body weight.
2: My parrot has a healthy appetite, though it is highly selective in what it eats, and/or it may drink more than what it normally does. My bird may either have maintained or gained body weight, or at most lost 5% of its body weight.
3: My parrot has a healthy appetite, eats the variety of different food items that I offer, and its water intake is within the normal range. My bird's body weight remains stable, showing some slight, daily fluctuations that are considered within the normal range (1-2%).

Would you change this score?

If you would change the score, please explain in the comment box how you would change it.
Choose one of the following answers

- ○ I agree with the score as it is described.
- ○ I disagree with the current scores and would change these as indicated in the text box (please fill out the text box)
- ● No answer

Please enter your comment here:

7 The animal-based indicator **"amount of time spent sleeping"** will be included in the assessment tool using the following scoring system.

How many hours per night does your parrot rest in complete darkness and without noises or sounds?
N/A: I don't know
0: Differs substantially each day, or there is no complete darkness and/or quiet time available for the parrot to rest
1: Less than 8h OR more than 14h
2: Between 8h and 10h OR between 12 and 14h
3: Between 10h to 12h

Would you change this score?

If you would change the score, please explain in the comment box how you would change it.
Choose one of the following answers

( ) I agree with the score as it is described.
( ) I disagree with the current scores and would change these as indicated in the text box (please fill out the text box)
(•) No answer

Please enter your comment here:

8 **Maintenance behaviours that did not pass the criteria for being considered among the most important parrot welfare indicators.**

Would you include any of the following measurements in the parrot welfare assessment tool?

YES - The indicator is valid for all parrot species and feasible for owners and I consider it to be one of the most important indicators of parrot welfare. I would include it in the tool.

MAYBE - The indicator is valid for all parrot species but is not feasible for owners and/or is not one of the most important indicators. I would include the indicator only in a different welfare assessment tool that considers more details.

NO - The measurement is not a valid indicator of welfare or it is valid only for some species. I would not include the measurements in a welfare assessment tool.

	Yes	Maybe	No	No answer
Preening activity (incl. time of day and frequency, duration)				●
Time of day (morning, afternoon, evening) spent sleeping/resting				●
Interest in bathing				●
Beak maintenance				●

10 Body measurements

11 The animal-based indicator **"number and appereance of droppings"** will be included in the assessment tool using the following scoring system:

Which statement best describes the droppings of your parrot?
N/A: I don't check droppings.
0: My parrot's droppings are scant in number and/or volume, and contain fresh blood or have a tardy black color, indicative of potential life-threatening illness.
1: My parrot's droppings are excessive in number, and/or are discolored, excessively watery and/or contain undigested food particles, indicative of potential digestive issues or other health concerns.
2: My parrot's droppings are mostly within the normal range, though some occasional or slight changes in color, odor, consistency or number can be observed.
3: My parrot's droppings are well-formed and of normal color, with a clear urate portion and minimal odor. The number of droppings are within my bird's normal elimination pattern, indicating optimal digestive health and hydration.

Would you change this score?

If you would change the score, please explain in the comment box how you would change it.
Choose one of the following answers

( ) I agree with the score as it is described.
( ) I disagree with the current scores and would change these as indicated in the text box (please fill out the text box)
(•) No answer

Please enter your comment here:

12 The animal-based indicator **"condition of body and flight feathers"** will be included in the assessment tool using the following scoring system adapted from the score developed by Mellor and colleagues (2022):

What is the condition of your parrot's plumage?
0: The plumage is severely damaged or plucked; my bird is (almost) completely devoid of its feathers
1: The plumage is moderately damaged or plucked; coverts and/or down feathers are missing in several areas of the body leaving a patchy distribution or coverts are missing but down is still mostly or completely present
2: The plumage is mildly damaged or plucked; coverts and/or down feathers are missing in focal areas, but most of the feathers are still intact (though some of these may show signs of damage)
3: The plumage is intact, there are no obvious signs of feather damage or plucking
Score adapted from Mellor et al. (2022)
Note: if the animal is housed with other parrots, possible feather damage could be caused by the other bird(s) rather than the bird itself; additionally, feather damage can be caused by features of the environment (e.g. smaller cage size).

Would you change this score?

If you would change the score, please explain in the comment box how you would change it.
Choose one of the following answers

( ) I agree with the score as it is described.
( ) I disagree with the current scores and would change these as indicated in the text box (please fill out the text box)
(•) No answer

Please enter your comment here:

13 The animal-based indicator **"mentation/alertness"** will be included in the assessment tool using the following scoring system:

What is your parrot's current mentation status or alertness level?
0: Lethargic - my parrot shows minimal responsiveness to stimuli, lacks interest in its surroundings, and exhibits signs of lethargy or disinterest in activities.
1: Moderately alert - my parrot responds to some stimuli but may require more encouragement to engage actively. It demonstrates moderate interest in its surroundings and participates in activities with moderate enthusiasm.
2: Alert - my parrot is alert and responsive to stimuli, showing interest in its surroundings and actively engaging in various activities. It readily interacts with toys, responds to social cues, and demonstrates curiosity and eagerness to explore its environment.
3: Highly alert and engaged - my parrot is highly alert, responsive, and mentally engaged. It shows strong interest in its surroundings, readily explores new stimuli, actively seeks out social interactions, and demonstrates problem-solving skills and cognitive flexibility.

Would you change this score?

If you would change the score, please explain in the comment box how you would change it.
Choose one of the following answers

( ) I agree with the score as it is described.
( ) I disagree with the current scores and would change these as indicated in the text box (please fill out the text box)
(•) No answer

Please enter your comment here:

14 **Body measurements that did not pass the criteria for being considered among the most important parrot welfare indicators.**

Would you include any of the following measurements in the parrot welfare assessment tool?

YES - The indicator is valid for all parrot species and feasible for owners and I consider it to be one of the most important indicators of parrot welfare. I would include it in the tool.

MAYBE - The indicator is valid for all parrot species but is not feasible for owners and/or is not one of the most important indicators. I would include the indicator only in a different welfare assessment tool that considers more details.

NO - The measurement is not a valid indicator of welfare or it is valid only for some species. I would not include the measurements in a welfare assessment tool.

	Yes	Maybe	No	No answer
Respiration/breathing changes (e.g., frequency, depth)				●
Posture (e.g., fluffed appearance, weight bearing)				●
Body weight				●
Pectoral muscle condition score				●
Eye appearance (e.g., half-open, discharge)				●
Gait changes (incl. flight)				●
Beak appearance (e.g., length, shape, position)				●
Cere/nare appearance (e.g., colour, shape, size)				●
Respiratory effort at rest				●
Respiratory effort when disturbed				●

Prolapses	○	○	○	●
Length and frequency of moulting	○	○	○	●

Meeting B: Abnormal and fear-related behaviours, body displays

In this section you are presented with a list of animal-based measures that will be discussed in the online meeting "abnormal and fear-related behaviours, body displays".

Some of these indicators already passed the criteria for being included in the final tool. These indicators will be presented to owners as questions with multiple choice or yes/no answers. The answers to these questions map to a scoring system with 4 or 5 points per question, where 0 represents the worst husbandry, management, body condition or behaviour, and 3 or 4 the best one. For these indicators, please indicate if you would modify the questions and corresponding scoring systems.

All the remaining indicators did not pass the criteria but can still be included in the final tool. Please indicate which of them you would include in the tool and suggest how you would rephrase them as questions for the owners, including the answer options.

16 Abnormal and fear-related behaviours

17 The animal-based indicators **"expression of avoidance or escape behaviours", "hiding", "tremors", "freezing" and "tonic/clonic immobility"** will be combined and included in the assessment tool by using the following scoring system:

How often/when does your parrot show signs indicative of fear and anxiety (i.e. tremors/shivering, freezing, hiding, withdrawing, escaping through flying or moving away, possibly falling of the perch, screeching/high pitched screams)?
0: most of the time, even when in its daily environment
1: regularly, though predominantly when exposed to changes in its daily environment (e.g. presence or approach by new, unfamiliar people/guests or animals, change of furniture, new house decorations, provision of new toys)
2: occasionally, and mostly when exposed to a novel situation, outside of its daily environment (e.g. outdoor activity, visit of a new place or to the vet)
3: rarely, and mostly in response to specific and uncommon situations (e.g. sudden loud noise)
4: never

Would you change this score?

If you would change the score, please explain in the comment box how you would change it.
Choose one of the following answers

○ I agree with the score as it is described.
○ I disagree with the current scores and would change these as indicated in the text box (please fill out the text box)
● No answer

Please enter your comment here:

18 The animal-based indicators **"excessive vocalisation/screaming"** will be included in the assessment tool by using the following scoring system:

How often does your parrot scream or produce excessive, loud vocalizations?
0: For the majority of of the day, and sometimes incessantly for hours with no apparent reason or cause
1: Frequently, and incessant screaming can occur but mostly in specific contexts (e.g. when left alone, presence of unfamiliar people/guests)
2: Occasionally, which can still be at several times during the day, but never incessantly and mostly when exposed to specific contexts (e.g. when left alone, presence of unfamiliar people/guests)
3: Rarely, but never incessantly, and mostly limited to specific moments of the day (e.g. morning and evening) or in response to specific and uncommon situations (e.g. sudden loud noise)
4: Never

Would you change this score?

If you would change the score, please explain in the comment box how you would change it.
Choose one of the following answers

○ I agree with the score as it is described.
○ I disagree with the current scores and would change these as indicated in the text box (please fill out the text box)
● No answer

Please enter your comment here:

19 **Abnormal behaviours that did not pass the criteria for being considered among the most important/feasible parrot welfare indicators.**
Would you include any of the following measurements in the parrot welfare assessment tool?

YES - The indicator is valid for all parrot species and feasible for owners and I consider it to be one of the most important indicators of parrot welfare. I would include it in the tool.

MAYBE - The indicator is valid for all parrot species but is not feasible for owners and/or is not one of the most important indicators. I would include the indicator only in a different welfare assessment tool that considers more details.

NO - The measurement is not a valid indicator of welfare or it is valid only for some species. I would not include the measurements in a welfare assessment tool.

	Yes	Maybe	No	No answer
Whole body stereotypies (head bobbing, rocking)	○	○	○	●
Locomotor stereotypies (route-tracing, pacing)	○	○	○	●
Sham behaviours (e.g., sham chewing, sham flying, sham bathing)	○	○	○	●
Excessive chewing (e.g., wires)	○	○	○	●
Toe/nail biting	○	○	○	●
Spot pecking	○	○	○	●
Excessive masturbation	○	○	○	●

21 Body displays

22 **None of the body displays passed the criteria for being considered among the most important/feasible parrot welfare indicators.**
Would you include any of the following measurements in the parrot welfare assessment tool?

YES - The indicator is valid for all parrot species and feasible for owners and I consider it to be one of the most important indicators of parrot welfare. I would include it in the tool.

MAYBE - The indicator is valid for all parrot species but is not feasible for owners and/or is not one of the most important indicators. I would include the indicator only in a different welfare assessment tool that considers more details.

NO - The measurement is not a valid indicator of welfare or it is valid only for some species. I would not include the measurements in a welfare assessment tool.

	Yes	Maybe	No	No answer
Ruffling of body feathers	○	○	○	●
Beak whipping across perch	○	○	○	●
Beak open all time	○	○	○	●
Wing flapping	○	○	○	●
Beak Grinding	○	○	○	●
Scratching	○	○	○	●
Absence of body displays	○	○	○	●
Cheek, beard feather ruffling	○	○	○	●

Meeting C: Housing and enrichment, locomotor and exploratory behaviours

In this section you are presented with a list of animal-based and environment-based measures that will be discussed in the online meeting "housing and enrichment, locomotor and exploratory behaviours".

Some of these indicators already passed the criteria for being included in the final tool. These indicators will be presented to owners as questions with multiple choice or yes/no answers. The answers to these questions map to a scoring system with 4 or 5 points per question, where 0 represents the worst husbandry, management, body condition or behaviour, and 3 or 4 the best one. For these indicators, please indicate if you would modify the questions and corresponding scoring systems.

All the remaining indicators did not pass the criteria but can still be included in the final tool. Please indicate which of them you would include in the tool and suggest how you would rephrase them as questions for the owners, including the answer options.

[24] Housing

25 The environmet-based indicator **"time spent out of the cage"** will be included in the assessment tool using the following scoring system:

How often does your parrot spend time out of its cage?
0: Never or less than once a week on average
1: Several times a week, but not every day
2: Every day, but the majority of the daytime is spent inside the cage (e.g. only out of the cage when someone is at home, out of the cage for <3 hours/day)
3: Most or all of the time (e.g. only in the cage when sleeping)

Would you change this score?

Choose one of the following answers

- ○ I agree with the score as it is described.
- ○ I disagree with the current scores and would change these as indicated in the text box (please fill out the text box)
- ● No answer

Please enter your comment here:

26 The environment-based indicator **"access to outdoor spaces"** will be included in the assessment tool using the following scoring system:

How often does your parrot spend time outdoor?
0: Never or (on average) less than once a week
1: Several days a week, but not every day
2: Daily for brief periods (<1 hour)
3: Daily for several (2+) hours

Would you change this score?

Choose one of the following answers

- ○ I agree with the score as it is described.
- ○ I disagree with the current scores and would change these as indicated in the text box (please fill out the text box)
- ● No answer

Please enter your comment here:

27 The environmet-based indicators **"cage characteristics"**, **"inability to fly due to cage size"** and **"opportunities to do physical exercise"** will be cobined and included in the assessment tool using the following scoring systems:

Which statement most accurately describes the size of your parrot's main enclosure (where it resides most of the daytime) and its ability to move around?

0: My parrot is unable to fully extend its wings in any direction, and has limited space to move
1: My parrot can fully extend its wings, but only in one direction, and has limited space available to climb
2: My parrot can fully extend its wings in all directions, and has space to climb and hop around
3: My parrot has ample space to move around, fly, hop and climb

What type of material is your parrot's cage made from?
0: Plastic-coated wire, zinc, wood or acrylic
1: Wrought iron
2: Powder-coated metal
3: Stainless steel

Which statement describes most accurately how the bars of your parrot's main enclosure are oriented?
0: There are no bars to allow climbing (e.g. solid panels made of glass, acrylic)
1: The enclosure has vertical bars only
2: The enclosure has horizontal bars only
3: The enclosure has a grid-patterned bars

Would you change this scores?

Choose one of the following answers

- ○ I agree with the score as it is described.
- ○ I disagree with the current scores and would change these as indicated in the text box (please fill out the text box)
- ● No answer

Please enter your comment here:

28 **Housing conditions that did not pass the criteria for being considered among the most important parrot welfare indicators.**

Would you include any of the following measurements in the parrot welfare assessment tool?

YES - The indicator is valid for all parrot species and feasible for owners and I consider it to be one of the most important indicators of parrot welfare. I would include it in the tool.

MAYBE - The indicator is valid for all parrot species but is not feasible for owners and/or is not one of the most important indicators. I would include the indicator only in a different welfare assessment tool that considers more details.

NO - The measurement is not a valid indicator of welfare or it is valid only for some species. I would not include the measurements in a welfare assessment tool.

	Yes	Maybe	No	No answer
Environmental temperature	○	○	○	●
Position of the cage in the room	○	○	○	●
Room where the cage is positioned (kitchen, living room, bedroom, etc.)	○	○	○	●
Exposure to artificial light at night	○	○	○	●
Perches' characteristics (e.g., diameter, material)	○	○	○	●
Position and height of the perches (e.g., in relation to feeders or human-eye level)	○	○	○	●
Presence of a retreating area/room to rest, sleep or withdraw	○	○	○	●
Air quality (e.g. presence of air purifier, exposure to fresh air)	○	○	○	●
Exposure to direct sunlight/UV light	○	○	○	●
Frequency of cage cleaning	○	○	○	●
Exposure to Noise	○	○	○	●
Environmental Humidity	○	○	○	●
Artificial light characteristics (e.g. type, intensity)	○	○	○	●
Presence of a nesting area	○	○	○	●
Size/number of windows in the room	○	○	○	●
Presence of a platform to stand on	○	○	○	●
Size of the room where the cage is positioned	○	○	○	●

[30] Enrichment

31 The environmet-based indicators "**provision of foraging enrichment**, "**manner/way in which food is offered to the bird (presented in a bowl, via enrichment, etc.)**" and "**variety of enrichment provided**" will be combined and included in the assessment tool using the following scoring systems:

Please indicate how often you provide foraging enrichment (i.e. toys, devices or other opportunities that stimulate the parrots' natural behaviour to search, procure and ex-tract food)?
0: Never or at most a few times per month, and limited to treats
1: Once to a few times per week, and limited to treats
2: Most days of the week, and limited to treats
3: Every day, not just for treats but also to provide the daily food

Please select which types of foraging enrichment you provide:
multiple food stations - scatter feeding - foraging tray or box (food mixed with inedible items) - larger chunks of food or whole food items - commercial puzzle feeders/foraging toys – DIY (do it yourself) destructible foraging toys

Would you change this score?

Choose one of the following answers

- ○ I agree with the score as it is described.

Please enter your comment here:

○ I disagree with the current scores and would change these as indicated in the text box (please fill out the text box)

● No answer

32 The environmet-based indicators "**provision of cognitive enrichment**" and "**variety of enrichment provided**" will be combined and included in the assessment tool using the following scoring systems:

Please indicate how often you train your bird, provide puzzles, games or other activities that provide mental stimulation.
0: Never or at most a few times per month
1: Once to a few times per week
2: Most days of the week, but not every day
3: Every day

Please select which types of cognitive enrichment you provide.
Training – Puzzles – Games

Would you change this score?

Choose one of the following answers

○ I agree with the score as it is described.

○ I disagree with the current scores and would change these as indicated in the text box (please fill out the text box)

● No answer

Please enter your comment here:

33 **Enrichment-related information that did not pass the criteria for being considered among the most important parrot welfare indicators.**

Would you include any of the following measurements in the parrot welfare assessment tool?

YES - The indicator is valid for all parrot species and feasible for owners and I consider it to be one of the most important indicators of parrot welfare. I would include it in the tool.

MAYBE - The indicator is valid for all parrot species but is not feasible for owners and/or is not one of the most important indicators. I would include the indicator only in a different welfare assessment tool that considers more details.

NO - The measurement is not a valid indicator of welfare or it is valid only for some species. I would not include the measurements in a welfare assessment tool.

	Yes	Maybe	No	No answer
Rotation of enrichment	○	○	○	●
Provision of chewable items	○	○	○	●
Amount of enrichment provided	○	○	○	●
Opportunities to select items based on preference (e.g., for colour, shape or type of material)	○	○	○	●
Provision of visual enrichment	○	○	○	●
Provision of auditory enrichment	○	○	○	●
Provision of climbing toys, swings and ladders	○	○	○	●
Area of the room/cage where enrichment is placed	○	○	○	●
Size of enrichment in relation to parrot size	○	○	○	●

35 Exploratory behaviours

36 The animal-based indicator "**interaction with enrichment**" will be included in the assessment tool using the following scoring system:

How often does your parrot interact (e.g. chew, play, manipulate) with the enrichment provided ?
0: (Almost) never
1: Once per week, < once per week
2: Occasionally or intermittently, bouts of intensive play are mixed with periods that the bird hardly interacts or at most 2-3 days per week
3: Regularly, i.e. most days, but at least several times per week (4+ days)
4: Daily

Would you change this score?

Choose one of the following answers

○ I agree with the score as it is described.

○ I disagree with the current scores and would change these as indicated in the text box (please fill out the text box)

● No answer

Please enter your comment here:

37 The animal-based indicators **"response to novel objects"** and **"response to eletronic devices"** will be combined and included in the assessment tool using the following scoring system:

How does your parrot react towards unfamiliar objects (e.g. new toys, new home decoration)?
0: It shows an extreme fear response (e.g. excessive, incessant screaming, frantic movements, flying away, falling of the perch), which happens with any type of unfamiliar object.
1: It shows signs indicative of fear (e.g. withdrawal, freezing) to most objects; certain objects (e.g. larger toys, larger furniture or electronic household devices) may trigger a more extreme fear response
2: It appears interested in the object, and cautiously approach it, after initially being reluctant to do so; certain objects (e.g. larger furniture or electronic household devices) may still trigger a mild to moderate fear response.
3: It actively approaches and explores the object (e.g. touching, chewing, manipulating); certain objects may still trigger a mild fear response.

Would you change this score?

Choose one of the following answers

○ I agree with the score as it is described.

○ I disagree with the current scores and would change these as indicated in the text box (please fill out the text box)

● No answer

Please enter your comment here:

38 **Exploratory behaviours that did not pass the criteria for being considered among the most important parrot welfare indicators.**

Would you include any of the following measurements in the parrot welfare assessment tool?

YES - The indicator is valid for all parrot species and feasible for owners and I consider it to be one of the most important indicators of parrot welfare. I would include it in the tool.

MAYBE - The indicator is valid for all parrot species but is not feasible for owners and/or is not one of the most important indicators. I would include the indicator only in a different welfare assessment tool that considers more details.

NO - The measurement is not a valid indicator of welfare or it is valid only for some species. I would not include the measurements in a welfare assessment tool.

	Yes	Maybe	No	No answer
Response in unfamiliar environments	○	○	○	●
Time spent foraging	○	○	○	●
Response to novel food items	○	○	○	●

40 Locomotor behaviours

41 The animal-based indicator **"ability to fly"** will be included in the assessment tool using the following scoring system:

Which statement most accurately reflects your parrot's ability to fly?
0: My parrot is not able to fly; it has received a unilateral wing trim or a bilateral wing trim which includes the primaries and secondaries, is deflighted using a surgical technique (e.g. pinioning), or is suffering from a chronic condition that permanently affect its ability to fly.
1: My parrot is only able to glide in a downwards flight over a short distance due to a bilateral, transverse wing trim and/or is permanently housed in an enclosure in which flight is not possible.
2: My parrot is fully flighted or has received a skinny wing trim bilaterally, and is allowed to fly indoors or in an aviary across short distances or for short periods during the day.
3: My parrot is fully flighted and is housed permanently in an aviary where it can fly, or is a free-flighted bird that is allowed to fly outdoors across larger distances or longer periods at a time.

Would you change this score?

Choose one of the following answers

○ I agree with the score as it is described.

Please enter your comment here:

○ I agree with the score as it is described.

○ I disagree with the current scores and would change these as indicated in the text box (please fill out the text box)

● No answer

Please enter your comment here:

42 **Locomotor behaviours that did not pass the criteria for being considered among the most important parrot welfare indicators.**

Would you include any of the following measurements in the parrot welfare assessment tool?

YES - The indicator is valid for all parrot species and feasible for owners and I consider it to be one of the most important indicators of parrot welfare. I would include it in the tool.

MAYBE - The indicator is valid for all parrot species but is not feasible for owners and/or is not one of the most important indicators. I would include the indicator only in a different welfare assessment tool that considers more details.

NO - The measurement is not a valid indicator of welfare or it is valid only for some species. I would not include the measurements in a welfare assessment tool.

	Yes	Maybe	No	No answer
Level of activity (time spent inactive vs walking, climbing, flying)	○	○	○	●
Time spent in high positions	○	○	○	●
Swinging	○	○	○	●

Meeting D: Human-animal interactions and human-directed behaviours

In this section you are presented with a list of animal-based and environment-based measures that will be discussed in the online meeting "human-animal interactions and human-directed behaviours".

Some of these indicators already passed the criteria for being included in the final tool. These indicators will be presented to owners as questions with multiple choice or yes/no answers. The answers to these questions map to a scoring system with 4 or 5 points per question, where 0 represents the worst husbandry, management, body condition or behaviour, and 3 or 4 the best one. For these indicators, please indicate if you would modify the questions and corresponding scoring systems.

All the remaining indicators did not pass the criteria but can still be included in the final tool. Please indicate which of them you would include in the tool and suggest how you would rephrase them as questions for the owners, including the answer options.

44 Human-animal interactions

45 The environment-based indicator **"rearing history"** will be included in the assessment tool using the following scoring system:

Please select the option that best describes your parrot's rearing history.
N/A: unknown
0: Wild-caught
1: Hand-reared without siblings
2: Hand-reared with siblings
3: Parent-reared

Would you change this score?

Choose one of the following answers

○ I agree with the score as it is described.

○ I disagree with the current scores and would change these as indicated in the text box (please fill out the text box)

● No answer

Please enter your comment here:

46 **Human-animal interactions that did not pass the criteria for being considered among the most important parrot welfare indicators.**

Would you include any of the following measurements in the parrot welfare assessment tool?

YES - The indicator is valid for all parrot species and feasible for owners and I consider it to be one of the most important indicators of parrot welfare. I would include it in the tool.

MAYBE - The indicator is valid for all parrot species but is not feasible for owners and/or is not one of the most important indicators. I would include the indicator only in a different welfare assessment tool that considers more details.

NO - The measurement is not a valid indicator of welfare or it is valid only for some species. I would not include the measurements in a welfare assessment tool.

	Yes	Maybe	No	No answer
Time spent on interaction with human	○	○	○	●
Type of interaction with human (training, mouth to beak feeding, etc.)	○	○	○	●
Frequency/duration of manual restraint	○	○	○	●
Number of people in the household that regularly interact with the parrot	○	○	○	●
Human produces loud noises and/or sudden movements	○	○	○	●
Time spent without presence of a human	○	○	○	●
Number of people in the household	○	○	○	●

48 Human-directed behaviours

49 The animal-based indicators **"response upon contact with caregiver"** , **"withdraw from human interaction"**, **"initiation of contact with humans"** and **"aggressive towards human"** will be combined and included in the assessment tool using the following scoring system:

How does your parrot respond when you (as the caregiver) approach it?
0: It (almost) always shows signs of aggression (e.g. lunging, attempting to bite) or tries to escape and/or avoid contact by withdrawing
1: It remains seated and stays in proximity, as long as I do not initiate physical contact; if I do attempt to initiate physical contact the bird will most of the time show signs of aggression or try to escape and avoid contact.
2: It remains seated and accepts physical contact initiated by me, but does not initiate contact itself and will still regularly display signs of aggression or try to withdraw from my attention.
3: It (almost) always accepts physical contact initiated by me, and will regularly initiate physical contact itself. It may occasionally show signs of aggression nor withdraws from my attention, but this is rare and/or predominantly happens when the interaction is inappropriate (e.g. touching of specific body parts, fast movement of the hands, heavy petting)

Would you change this score?

Choose one of the following answers

○ I agree with the score as it is described.

○ I disagree with the current scores and would change these as indicated in the text box (please fill out the text box)

● No answer

Please enter your comment here:

50 The animal-based indicators **"response upon contact with familiar person"** , **"withdraw from human interaction"**, **"initiation of contact with humans"** and **"aggressive towards human"** will be combined and included in the assessment tool using the following scoring system:

How does your parrot respond when a familiar person (e.g. partner, family member, friend) approaches it?
0: It (almost) always shows signs of aggression (e.g. lunging, attempting to bite) or tries to escape and/or avoid contact by withdrawing
1: It remains seated and stays in proximity, as long as the person does not initiate physical contact; if he/she/they do attempt to initiate physical contact the bird will most of the time show signs of aggression or try to escape and avoid contact.
2: It remains seated and accepts physical contact initiated by the person, but does not initiate contact itself and will still regularly display signs of aggression or try to withdraw from her/his/their attention.
3: It (almost) always accepts physical contact initiated by the person, and will regularly initiate physical contact itself. It may occasionally show signs of aggression or withdraws from her/his/their attention, but this is rare and/or predominantly happens when the interaction is inappropriate (e.g. touching of specific body parts, fast movement of the hands, heavy petting)

Would you change this score?

Choose one of the following answers

○ I agree with the score as it is described.

○ I disagree with the current scores and would change these as indicated in the text box (please fill out the text box)

● No answer

Please enter your comment here:

51 **Human-directed behaviours that did not pass the criteria for being considered among the most important parrot welfare indicators.**

Would you include any of the following measurements in the parrot welfare assessment tool?

YES - The indicator is valid for all parrot species and feasible for owners and I consider it to be one of the most important indicators of parrot welfare. I would include it in the tool.

MAYBE - The indicator is valid for all parrot species but is not feasible for owners and/or is not one of the most important indicators. I would include the indicator only in a different welfare assessment tool that considers more details.

NO - The measurement is not a valid indicator of welfare or it is valid only for some species. I would not include the measurements in a welfare assessment tool.

	Yes	Maybe	No	No answer
Food-related interaction (e.g., begging for food, ac-ceptance of food from the hand, regurgitation of food to humans)	○	○	○	●
Sex-related behaviours (e.g., panting, receptive posture)	○	○	○	●
Contact seeking behaviours in absence of humans (e.g., vocalization, flapping wings)	○	○	○	●
Ruffling of feathers (e.g., nape, crown, beard) during human interaction	○	○	○	●
Response upon contact with unfamiliar person	○	○	○	●
Abnormal behaviours in presence of humans	○	○	○	●
Aggression towards humans linked to a specific loca-tion or perimeter	○	○	○	●

Meeting E: Social needs, social and sex-related behaviours

In this section you are presented with a list of animal-based and environment-based measures that will be discussed in the online meeting "social needs, social and sexual-related behaviours".

Some of these indicators already passed the criteria for being included in the final tool. These indicators will be presented to owners as questions with multiple choice or yes/no answers. The answers to these questions map to a scoring system with 4 or 5 points per question, where 0 represents the worst husbandry, management, body condition or behaviour, and 3 or 4 the best one. For these indicators, please indicate if you would modify the questions and corresponding scoring systems.

All the remaining indicators did not pass the criteria but can still be included in the final tool. Please indicate which of them you would include in the tool and suggest how you would rephrase them as questions for the owners, including the answer options.

53 Social needs

54 The environmet-based indicators **"social housing (alone vs pair vs group)"** and "**level of social contact**" will be combined and included in the assessment tool using the following scoring system:

Please select the option that best describes your parrot's social contact with other birds
0: My parrot lives alone and does not have any type of contact with other parrots
1: My parrot is housed individually, but is able to see other parrots and can vocally interact with them
2: My parrot can physically interact with one or more other parrots at certain times of the day (or week), but is not continuously housed together with other birds
3: My parrot is housed together with one other parrot and can continuously interact with this bird.
4: My parrot is housed together with two or more other parrots and can continuously interact with these birds.

Would you change this score?

Choose one of the following answers

○ I agree with the score as it is described.
○ I disagree with the current scores and would change these as indicated in the text box (please fill out the text box)
● No answer

Please enter your comment here:

55 **Social needs that did not pass the criteria for being considered among the most important parrot welfare indicators.**

Would you include any of the following measurements in the parrot welfare assessment tool?

YES - The indicator is valid for all parrot species and feasible for owners and I consider it to be one of the most important indicators of parrot welfare. I would include it in the tool.

MAYBE - The indicator is valid for all parrot species but is not feasible for owners and/or is not one of the most important indicators. I would include the indicator only in a different welfare assessment tool that considers more details.

NO - The measurement is not a valid indicator of welfare or it is valid only for some species. I would not include the measurements in a welfare assessment tool.

	Yes	Maybe	No	No answer
Type of social companionship (same vs different in terms of species, size, sex, origin, etc.)	○	○	○	●
Opportunities for pair bonding (i.e., living with/without a mate)	○	○	○	●
Frequency/duration of social separation events	○	○	○	●
Partner/cage mate choice (free vs imposed)	○	○	○	●

57 Social behaviours

58 The animal-based indicator **"time spent in vicinity of other parrots"** will be included in the assessment tool using the following scoring system:

How often does your parrot engage in social interaction with other parrots?
0: It rarely engages in social interaction and actively avoids the other birds.
1: It accepts their presence, allows approach by the other birds, and occasionally engages in social interaction.
2: It frequently engages in social interaction and actively approaches and seeks contact with the other birds.
3: It is socially engaged with the other birds for most of the time, actively approaching the other birds to stay in their vicinity.

Would you change this score?

Choose one of the following answers

○ I agree with the score as it is described.
○ I disagree with the current scores and would change these as indicated in the text box (please fill out the text box)
● No answer

Please enter your comment here:

59 **Social behaviours that did not pass the criteria for being considered among the most important parrot welfare indicators.**

Would you include any of the following measurements in the parrot welfare assessment tool?

YES - The indicator is valid for all parrot species and feasible for owners and I consider it to be one of the most important indicators of parrot welfare. I would include it in the tool.

MAYBE - The indicator is valid for all parrot species but is not feasible for owners and/or is not one of the most important indicators. I would include the indicator only in a different welfare assessment tool that considers more details.

NO - The measurement is not a valid indicator of welfare or it is valid only for some species. I would not include the measurements in a welfare assessment tool.

	Yes	Maybe	No	No answer
Frequency and duration of social interactions (e.g. play behaviour, vocal communication, allopreening)	○	○	○	●
Aggressive behaviour toward mates (e.g., chasing, bit-ing, lunging)	○	○	○	●
Aggressive behaviour toward non-mates (e.g., chas-ing, biting, lunging)	○	○	○	●
Aggressive behaviour toward mates chicks	○	○	○	●
Displacement behaviours during social interactions	○	○	○	●

61 ## Sex-related behaviours

62 **None of the sex-related behaviours passed the criteria for being considered among the most important parrot welfare indicators.**

Would you include any of the following measurements in the parrot welfare assessment tool?

YES - The indicator is valid for all parrot species and feasible for owners and I consider it to be one of the most important indicators of parrot welfare. I would include it in the tool.

MAYBE - The indicator is valid for all parrot species but is not feasible for owners and/or is not one of the most important indicators. I would include the indicator only in a different welfare assessment tool that considers more details.

NO - The measurement is not a valid indicator of welfare or it is valid only for some species. I would not include the measurements in a welfare assessment tool.

	Yes	Maybe	No	No answer
Physical proximity between mates	○	○	○	●
Mate allopreening	○	○	○	●
Courtship feeding	○	○	○	●
Nest Defense	○	○	○	●
Copulation	○	○	○	●
Territoriality during breeding season	○	○	○	●
Searching for nesting areas	○	○	○	●
Masturbation	○	○	○	●

Submit

vetmeduni

Resume later

Prototype Parrot Welfare Assessment Tool

Information and Consent

vetmeduni
University of Veterinary Medicine, Vienna

Utrecht University

Dear expert,
Thank you for taking part in this survey!
The aim of this survey is to review the prototype of the welfare assessment tool, developed based on the results of the focus group meetings, in terms of its completeness and clarity.

This survey displays all the welfare indicators (both animal-based and environment-based) that will be included in the assessment tool. The indicators are organized into different welfare dimensions, which are distributed across 8 sections. The information is presented in the same structure in which it will be shown to the owners.
Each question is accompanied by a comment box where you can provide your feedback and suggestions. For questions presented in table format, a comment box is available directly below the table. Please note the following:
•Indicators in green were thoroughly discussed during the meetings. You agreed on their inclusion and phrasing as questions, so they may **only require minor revisions**.
•Indicators in red were also agreed upon for inclusion in the prototype but **require further attention**, as there was insufficient time during the meetings to finalize their phrasing.

At the end of each section, you'll find a comment box where you can **note whether the section feels complete or if you believe something essential is missing**.
In the final section of the survey, titled 'Sections Organization', you can **share your feedback on the overall structure**. You can indicate whether you agree with the current order of the sections, suggest changes to the sequence, or propose moving specific indicators or welfare dimensions to different sections.

You have the **opportunity to save partially finished surveys** by clicking on the top right button saying "Resume later" that you can find in all pages of the survey. To open the partially saved survey, click on the link to the survey that you received by email.

Please note that:

- Participation is voluntary and anonymous.
- You can withdraw at any moment of the study.
- You have to be at least 18 years old to participate.
- The results of this survey will be included in a PhD thesis and published in a scientific journal.
- Your name will not appear in any published documents related to this study, and your statements will remain anonymous.
- Your personal data will be treated in compliance with the European General Data Protection Regulation and will be stored only as long as needed for the purpose of this study.

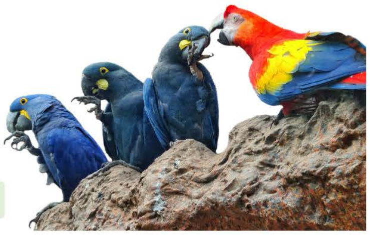

If you have any questions or concerns, please do not hesitate to contact the responsible person of this study:

Andrea Piseddu
Centre for animal Nutrition and Welfare, University of Veterinary Medicine Vienna, Vienna, Veterinaerplatz 1, 1210 Vienna
Andrea.Piseddu@vetmeduni.ac.at

By clicking the button "**Next**" and submitting your responses to the survey you are confirming to have read and understood the above statements and give your informed consent.

There are 89 questions in this survey.

Section 1: General information

Each question is accompanied by a comment box where you can provide your feedback and suggestions. For questions presented in table format, a comment box is available directly below the table. Please note the following:

• Indicators in green were thoroughly discussed during the meetings. You agreed on their inclusion and phrasing as questions, so they may **only require minor revisions**.
• Indicators in red were also agreed upon for inclusion in the prototype but **require further attention**, as there was insufficient time during the meetings to finalize their phrasing.

Name of your parrot

Species of your parrot

Sex

Choose one of the following answers

- Female
- Male
- Unknown

How old is your parrot?

Only numbers may be entered in this field.

How many years have you been living with your parrot?

Only numbers may be entered in this field.

How did you get your parrot from?

Choose one of the following answers

- Pet store
- Breeder
- Shelter or rescue organization
- Private person
- Other:

Health check up

How often does your parrot visit a veterinarian for general health check-up?

The answer options range from the worst conditions at the top to the best conditions at the bottom

Choose one of the following answers

- Never went for a general check-up
- Less than once per year
- Once per year
- Two or more times per year

Please enter your comment here:

Is there any essential information missing from this section, or any non-essential information that could be removed?

If so, please provide your input in the comment box.

Section 2: Body measurements

Each question is accompanied by a comment box where you can provide your feedback and suggestions. For questions presented in table format, a comment box is available

directly below the table. Please note the following:

• **Indicators in green** were thoroughly discussed during the meetings. You agreed on their inclusion and phrasing as questions, so they may **only require minor revisions**.
• **Indicators in red** were also agreed upon for inclusion in the prototype but **require further attention**, as there was insufficient time during the meetings to finalize their phrasing.

Condition of flight and body feathers (minor revisions only)

What is the condition of your parrot's plumage?

The answer options range from the worst conditions at the top to the best conditions at the bottom
Choose one of the following answers

- The plumage is severely damaged or plucked; my bird is (almost) completely devoid of its feathers
- The plumage is moderately damaged or plucked; coverts and/or down feathers are missing in several areas of the body leaving a patchy distribution or coverts are missing but down is still mostly or completely present
- The plumage is mildly damaged or plucked; coverts and/or down feathers are missing in focal areas, but most of the feathers are still intact (though some of these may show signs of damage)
- The plumage is intact, there are no obvious signs of feather damage or plucking

Please enter your comment here:

Number and appearance of droppings (minor revisions only)

Which statement best describes the droppings of your parrot?

The answer options range from the worst conditions at the top to the best conditions at the bottom
Choose one of the following answers

- I don't check droppings.
- My parrot's droppings are scant in number and/or volume, and contain fresh blood or have a tardy black colour, indicative of potential life-threatening illness.
- My parrot's droppings are excessive in number, and/or are discoloured, excessively watery and/or contain undigested food particles, indicative of potential digestive issues or other health concerns.
- My parrot's droppings are mostly within the normal range, though some occasional or slight changes in colour, odour, consistency or number can be observed.
- My parrot's droppings are well-formed and of normal colour, with a clear urate portion and minimal odour. The number of droppings are within my bird's normal elimination pattern, indicating optimal digestive health and hydration.

Please enter your comment here:

Pectoral muscle condition score

Please select one the following score according to the muscle condition of your parrot.
If you don't know how to calculate the score consult this link: https://www.ukpetfood.org/resource/bird-size-o-meter.html

The answer options range from the worst conditions at the top to the best conditions at the bottom. Very thin has equal value of obese, thin has equal value of overweight.
Choose one of the following answers

- I can't calculate the score because my bird does not accept to be handled.
- Very thin: breast bone is very sharp to the touch
- Obese: very hard or not possible to feel the breast bone, very rounded muscle and possible to feel or see fat moving under the skin.
- Thin: breast bone is easily felt and sharp
- Overweight: Pressure is needed to feel the breast bone, well rounded breast muscle and some fat cover
- Ideal: breast bone easily felt but not sharp

Please enter your comment here:

Signs of illness

Do you notice any of these conditions?

	Yes	No	I am not sure
Overgrow of beak and/or nails			
Beak malocclusion			
Redness, swelling or loss of feathers around eyes			
Crusty material in or around nares			
Flakiness on skin or beak			
Unusual or dull feather color			
Presence of horizontal lines across the feathers			
Lameness or shifting of body weight			
Blood loss or injury			
Discharge from nares (nostrils), eyes, or mouth			
Labored breathing or abnormal respiratory sounds			
Enlargement or swelling on the body			
Vomiting or regurgitation			
Fluffed posture and sleeping more than normal			
Inability to perch			
Increase or decrease of body weight >10% compared to last week			
Cloaca prolapse			
Swelling, ulcers, or lesions under the feet			

Comment box for:
Signs of illness

Is there any essential information missing from this section, or any non-essential information that could be removed?

If so, please provide your input in the comment box.

Section 3: Housing and physical activity

Each question is accompanied by a comment box where you can provide your feedback and suggestions. For questions presented in table format, a comment box is available directly below the table. Please note the following:

• **Indicators in green** were thoroughly discussed during the meetings. You agreed on their inclusion and phrasing as questions, so they may **only require minor revisions**.
• **Indicators in red** were also agreed upon for inclusion in the prototype but **require further attention**, as there was insufficient time during the meetings to finalize their phrasing.

phrasing.

Enclosure type

Which type of enclosure (space where the parrot sleeps, eats, drinks, and stays when it can't be supervised) do you provide for your parrot?

Choose one of the following answers

- ○ Indoor cage
- ○ Outdoor cage
- ○ Room exclusively dedicated to the parrot
- ○ Parrot lives indoor and does not have a dedicated enclosure
- ○ Outdoor aviary
- ○ Indoor aviary

Please enter your comment here:

Enclosure size (minor revisions only)

Which statement most accurately describes the size of your parrot's main enclosure (where it resides most of the daytime) and its ability to move around?

The answer options range from the worst conditions at the top to the best conditions at the bottom
Choose one of the following answers

- ○ My parrot is unable to fully extend its wings in any direction, and has limited space to move
- ○ My parrot can fully extend its wings, but only in one direction, and has limited space available to climb and hop around
- ○ My parrot can fully extend its wings in all directions, and has space to climb and hop around
- ○ My parrot has enough space to move, fly, hop and climb around

Please enter your comment here:

Opportunity to spent time outside the enclosure (minor revisions only)

How often does your parrot spend time out of its enclosure (cage, bird-friendly room or aviary where the animal can rest, eat and drink)?

The answer options range from the worst conditions at the top to the best conditions at the bottom
Choose one of the following answers

- ○ Never or less than once a week on average
- ○ Several times a week, but not every day
- ○ Every day, for 3 hours or less
- ○ Every day, for 3 hours or more
- ○ Most or all of its time (e.g. only in the enclosure when sleeping or when there is no human supervision)

Please enter your comment here:

Area where the enclosure is positioned

Where is the indoor cage/aviary of your parrot located?

The answer options range from the worst conditions at the top to the best conditions at the bottom
Choose one of the following answers

- ○ Kitchen
- ○ Bathroom
- ○ Bedroom
- ○ Hallway
- ○ Living room

Please enter your comment here:

Enclosure material (minor revisions only)

What type of material is your parrot's enclosure made from?

The answer options range from the worst conditions at the top to the best conditions at the bottom
Choose one of the following answers

- ○ I don't know
- ○ Plastic-coated wire, zinc, wood or acrylic
- ○ Wrought iron
- ○ Powder-coated metal
- ○ Stainless steel

Please enter your comment here:

Bars orientation (minor revisions only)

Which statement describes most accurately how the bars of your parrot's enclosure are oriented?

The answer options range from the worst conditions at the top to the best conditions at the bottom
Choose one of the following answers

- ○ There are no bars to allow climbing (e.g. solid panels made of glass, acrylic)
- ○ The enclosure has horizontal bars only
- ○ The enclosure has vertical bars only
- ○ The enclosure has grid-patterned bars

Please enter your comment here:

Number of perches

How many perches are available in the parrot's enclosure?

The answer options range from the worst conditions at the top to the best conditions at the bottom
Choose one of the following answers

- ○ There are no perches
- ○ One – two
- ○ Three – five
- ○ More than five

Please enter your comment here:

Perches' characteristics (e.g., diameter, material)

Did you check if the material of the perches is safe and if their diameter is suitable for your parrot's species?

Choose one of the following answers

- ○ Yes
- ○ No

Please enter your comment here:

Hygiene: frequency of cleaning food and water bowls, foraging toys, enclosure

How often do you clean...

The answer options range from the worst conditions on the left to the best conditions on the right

	Once a week or less	2-3 times per week or less	Several times a week, but not every day	Daily or after every use
Enclosure (cage, room, aviary)	○	○	○	○
Food bowls	○	○	○	○
Water bowl	○	○	○	○

Water bowl				
Foraging toys				

Comment box for

Hygene: frequency of food and water bowls, foraging toys, enclosure cleaning

Climate (temperature. humidity)

How often do you check if the following environmental conditions are appropriate for your parrot's species?

The answer options range from the worst conditions on the left to the best conditions on the right

	Once a week or less	2-3 times a week	Several times a week, but not every day	Daily
Environmental temperature				
Environmental humidity				

Comment box for:

Climate (temperature. humidity)

Air quality

How often do you refresh the air in the area where your parrot lives and spends most of its time?

The answer options range from the worst conditions at the top to the best conditions at the bottom

Choose one of the following answers

- Once per week or less
- Several times a week, but not every day
- Once per day / I use an air purifier but is not constantly activated
- More than once per day / I use an air purifier / My parrot lives outdoor

Please enter your comment here:

Access to outdoor spaces (minor revisions only)

How often does your parrot spend time outdoor, if environmental circumstances permit (no risk of predation or mosquitoes' bites, appropriate temperature)?

The answer options range from the worst conditions at the top to the best conditions at the bottom

Choose one of the following answers

- Never
- Less than once a week on average
- At least one a week
- At least once a day

Please enter your comment here:

Exposure to direct sunlight/UV light

How often is your parrot exposed to UV lamp light or direct sunlight without a window barrier in between?

The answer options range from the worst conditions at the top to the best conditions at the bottom

Choose one of the following answers

- Never
- Once per week or less
- Several times a week, but not every day
- Everyday

Please enter your comment here:

Frequency caregiver brings parrot with them

How often do take your parrot with you when you go out?

The answer options range from the worst conditions at the top to the best conditions at the bottom

Choose one of the following answers

- Never
- Less than once per month on average
- Less than once a week on average
- Weekly

Please enter your comment here:

Presence of a retreating area/room to rest, sleep or withdraw (minor revisions only)

Does your parrot have unlimited access to an undisturbed area where it can rest, sleep or retreat from potential stressful situations (presence of unfamiliar people, other animals etc.)?

Choose one of the following answers

- Yes
- No

Please enter your comment here:

Opportunity to spend time in high positions

Does your parrot have the opportunity to perch in high locations that are out of reach of people and other animals?

Choose one of the following answers

- Yes
- No

Please enter your comment here:

Time spent in high positions

How much time does your parrot spend in high locations that are out of reach of people and other animals when you provide the opportunity to do so?

The answer options range from the worst conditions at the top to the best conditions at the bottom

Choose one of the following answers

- All its time and it does not move
- Most of its time, only moving to lower ones when people (including myself) or other pets are not nearby
- All the time when unfamiliar people or animal are in its close proximity, other-wise more or less the same amount of time that it spends in heights that are reachable
- More or less the same amount of time that it spends in heights that are reachable
- Some time but not more of the time spent in heights that are reachable

Please enter your comment here:

Provision of opportunities that allow movement and climbing

How often does your parrot receive enrichment that encourages movement and climbing (e.g., ladders, branches, swings)?

The answer options range from the worst conditions at the top to the best conditions at the bottom

Choose one of the following answers

- Never or at most a few times per month
- Once to a few times per week
- Most days of the week
- Every day

Please enter your comment here:

Ability to fly

Which statement most accurately reflects your parrot's ability to fly?

The answer options range from the worst conditions at the top to the best conditions at the bottom

Choose one of the following answers

- My parrot is not able to fly; it has received a unilateral wing trim or a bilateral wing trim which includes the primaries and secondaries, is deflighted using a surgical technique (e.g. pinioning), is suffering from a chronic condition that permanently affect its ability to fly, or is fully flighted but it has never learned to fly.
- My parrot is only able to fly downwards over a short distance due to a bilateral, transverse wing trim
- My parrot has restricted flying capacity due to a bilateral skinny wing trim
- My parrot is fully flighted

Please enter your comment here:

Opportunity to fly (minor revisions only)

How often does your parrot have the opportunity to fly in a safe space?

The answer options range from the worst conditions at the top to the best conditions at the bottom

Choose one of the following answers

- Never or less than once a week on average
- Several times a week, but not every day
- Every day for less than 4 hours
- Every day for 4 or more hours

Please enter your comment here:

Level of activity

How much time does your parrot spend each day moving, climbing, and flying?

Choose one of the following answers

- I don't know
- 1 hour or less
- Between 1 and 2 hours
- Between 2 and 4 hours
- 4 or more hours

Please enter your comment here:

Is there any essential information missing from this section, or any non-essential information that could be removed?

If so, please provide your input in the comment box.

Section 4: Provision of enrichment and exploration

Each question is accompanied by a comment box where you can provide your feedback and suggestions. For questions presented in table format, a comment box is available directly below the table. Please note the following:

- **Indicators in green** were thoroughly discussed during the meetings. You agreed on their inclusion and phrasing as questions, so they may **only require minor revisions**.
- **Indicators in red** were also agreed upon for inclusion in the prototype but **require further attention**, as there was insufficient time during the meetings to finalize their phrasing.

Provision of foraging enrichment (minor revisions only)

How often do you provide foraging enrichment (i.e. toys, devices or other opportunities that stimulate the parrots' natural behaviour to search, procure and extract food)?

The answer options range from the worst conditions at the top to the best conditions at the bottom

Choose one of the following answers

- Never or at most a few times per month, and limited to treats
- Once to a few times per week, and limited to treats
- Most days of the week, and limited to treats
- Every day, not just for treats but also to provide part of the daily food ration

Please enter your comment here:

Types of foraging enrichment provided (minor revisions only)

Please select which types of foraging enrichment you provide:

The more options are selected the better

Comment only when you choose an answer.

- Multiple food stations
- Scatter feeding
- Foraging tray or box (food mixed with inedible items)
- Larger chunks of food or whole food items
- Commercial puzzle feeders/foraging toys
- DIY (do it yourself) destructible foraging

Provision of cognitive enrichment (minor revisions only)

Please indicate how often you train your bird, provide puzzles, games or other activities that provide mental stimulation

The answer options range from the worst conditions at the top to the best conditions at the bottom

Choose one of the following answers

- Never or at most a few times per month
- Once to a few times per week
- Most days of the week
- Every day

Please enter your comment here:

Types of cognitive enrichment provided (minor revisions only)

Please select which types of cognitive enrichment you provide:

❓ The more options are selected the better
❗ Comment only when you choose an answer.

- ☐ Training
- ☐ Puzzles
- ☐ Games

Opportunities to select items based on preference (e.g., for colour, shape or type of material) (minor revisions only)

Do you give your parrot the opportunity to choose its toys based on colour, shape, or material?

❗ Choose one of the following answers

- ◯ Yes
- ◯ No

Please enter your comment here:

Enrichment replacement

How often do you replace toys/chewable items/ climbing enrichment by introducing new different one/s

❓ The answer options range from the worst conditions at the top to the best conditions at the bottom
❗ Choose one of the following answers

- ◯ Never, I always use the same and I usually replace them only when they are consumed or damaged
- ◯ Less than once per month
- ◯ Few times per month
- ◯ Every week

Please enter your comment here:

Provision of chewable items

How often do you provide chewable items (certified chewable toys, carboard or paper without ink, natural, not toxic and untreated cork and branches) that your parrot can safely chew and destroy with its beak?

❓ The answer options range from the worst conditions at the top to the best conditions at the bottom
❗ Choose one of the following answers

- ◯ Never or at most a few times per month
- ◯ Once to a few times per week
- ◯ Most days of the week
- ◯ Every day

Please enter your comment here:

Interaction with enrichment (minor revisions only)

How often does your parrot interact (e.g. chew, play, manipulate) with the enrichment provided?

❓ The answer options range from the worst conditions at the top to the best conditions at the bottom
❗ Choose one of the following answers

- ◯ (Almost) never
- ◯ Once per week or less
- ◯ Occasionally or intermittently, bouts of intensive play are mixed with periods that the parrot hardly interacts or at most 2-3 days per week
- ◯ Regularly, i.e. most days, but at least several times per week (4+ days)
- ◯ Daily

Please enter your comment here:

Time spent foraging (minor revisions only)

How much time does your parrot spend daily foraging (i.e., searching for, procuring, and extracting food from toys that you provide)?

❓ The answer options range from the worst conditions at the top to the best conditions at the bottom
❗ Choose one of the following answers

- ◯ I don't know
- ◯ It does not forage
- ◯ Up to 1 hour
- ◯ Between 1 and 3 hours
- ◯ Between 3 and 4 hours
- ◯ 4 hours or more

Please enter your comment here:

Response to novel objects (minor revisions only)

How does your parrot generally react towards unfamiliar objects (e.g. new toys, new home decoration) in its proximity?

❓ The answer options range from the worst conditions at the top to the best conditions at the bottom
❗ Choose one of the following answers

- ◯ It shows one or more of these behaviours: excessive or incessant screaming, frantic movements, flying away, falling of the perch. This response happens with any type of unfamiliar object.
- ◯ It withdraws or freezes to most objects; certain objects (e.g. larger toys, larger furniture or electronic household devices) may trigger more freezing or withdrawing responses.
- ◯ It appears interested in the object, and cautiously approach it, after initially being reluctant to do so; certain objects (e.g. larger furniture or electronic household devices) may still trigger freezing or withdrawing responses.
- ◯ It actively approaches the object touching, chewing and manipulating it; certain objects may still trigger freezing or withdrawing responses.

Please enter your comment here:

Mentation/alertness (minor revisions only)

What is your parrot's current mentation status or alertness level?

❓ The answer options range from the worst conditions at the top to the best conditions at the bottom
❗ Choose one of the following answers

- ◯ Lethargic - my parrot shows minimal responsiveness to stimuli, lacks interest in its surroundings, and exhibits signs of lethargy or disinterest in activities.
- ◯ Moderately alert - my parrot responds to some stimuli but may require more encouragement to engage actively. It demonstrates moderate interest in its surroundings and participates in activities with moderate enthusiasm.
- ◯ Alert - my parrot is alert and responsive to stimuli, showing interest in its surroundings and actively engaging in various activities. It readily interacts with toys, responds to social cues, and demonstrates curiosity and eagerness to explore its environment.
- ◯ Highly alert and engaged - my parrot is highly alert, responsive, and mentally engaged. It shows strong interest in its surroundings, readily explores new stimuli, actively seeks out social interactions, and demonstrates problem-solving skills and cognitive flexibility.

Please enter your comment here:

Is there any essential information missing from this section, or any non-essential information that could be removed?

[illegible]

If so, please provide your input in the comment box.

Section 5: Nutrition and maintenance behaviours

Each question is accompanied by a comment box where you can provide your feedback and suggestions. For questions presented in table format, a comment box is available directly below the table. Please note the following:

• Indicators in green were thoroughly discussed during the meetings. You agreed on their inclusion and phrasing as questions, so they may **only require minor revisions**.
• Indicators in red were also agreed upon for inclusion in the prototype but **require further attention**, as there was insufficient time during the meetings to finalize their phrasing.

Composition of the diet

Which of the following foods do you provide?

Best response depends on the number of items and type of answer options selected and greatly varies on the species

	Main component of the diet	Moderate amount	Small/limited quantities	As treat during training activities or as a reward for desired behaviours	Not provided
Fresh fruit	○	○	○	○	○
Fresh vegetables	○	○	○	○	○
Pellet	○	○	○	○	○
Seed mix	○	○	○	○	○
Nuts	○	○	○	○	○
Sprouted seeds	○	○	○	○	○
Eggs	○	○	○	○	○
Legumes	○	○	○	○	○
Grains	○	○	○	○	○
Nectar	○	○	○	○	○
Processed food specifically designed for human consumption (high in sugar or fat content)	○	○	○	○	○

Comment box for:

Composition of the diet

Variety of food eaten

Does your parrot eat all types of food that you provide?
observing the animal while it eats is preferable, but checking the food bowl can also provide valuable insights.

Choose one of the following answers

○ I don't know
○ No
○ Yes

Please enter your comment here:

Availability of fresh and clean water

How often do you change your parrot's water?

The answer options range from the worst conditions at the top to the best conditions at the bottom
Choose one of the following answers

○ Less than once per day
○ Once a day
○ Twice a day
○ More than twice a day or as needed throughout the day

Please enter your comment here:

Diet Apropriateness

Have you checked whether the diet you provide is balanced and appropriate for your parrot's species?

The answer options range from the worst conditions at the top to the best conditions at the bottom
Choose one of the following answers

○ No
○ Yes, I researched by myself online, in books, or magazines
○ Yes, I consulted my veterinarian or a behavioural consultant

Please enter your comment here:

Changes in food and/or water consuption

Does your parrot show a lost of appetite or a decrease/increase of water consumption?
i.e. you observed these changes directly observing the parrot's behaviour or by checking the amount of food or water consumed.

Choose one of the following answers

○ No
○ Yes
○ I don't know

Please enter your comment here:

Time of the day spent sleeping / resting

What time of day has your parrot been sleeping / resting recently?

The more answer options are selected the worse
Comment only when you choose an answer.

☐ I don't know
☐ Morning
☐ Afternoon
☐ Evening
☐ Night

Changes of resting-sleeping patterns

Do you notice any change in the sleeping pattern of your parrot?
e.g. sleeping more than usual or at times of the day when it typically does not sleep.

Choose one of the following answers

- I don't know
- No
- Yes

Please enter your comment here:

Interest in bathing
Does your parrot bathe?

Choose one of the following answers

- I don't know
- No
- Yes

Please enter your comment here:

Opportunity to bathe
Please indicate the bathing opportunities provided to your parrot

None = worst answer, the others are equal
Comment only when you choose an answer.

- None
- Mist
- Shower
- Bath
- Other

Beak maintenance (1)

Choose one of the following answers

Please enter your comment here:

Changes in preening activity (indicator not discussed in the previous meetings)
Have you noticed any changes in your parrot's preening behavior (using its beak to clean, arrange, and maintain its feathers)?

The answer options range from the worst conditions at the top to the best conditions at the bottom
Choose one of the following answers

- I don't know
- Yes, my parrots stopped preening or it is preening very often or excessively during the day
- My parrot rarely preens or does not preen at all
- No, my parrot preens daily but never excessively

Please enter your comment here:

Is there any essential information missing from this section, or any non-essential information that could be removed?
If so, please provide your input in the comment box.

Section 6: Social needs, social and reproductive behaviours

Each question is accompanied by a comment box where you can provide your feedback and suggestions. For questions presented in table format, a comment box is available directly below the table. Please note the following:

- **Indicators in green** were thoroughly discussed during the meetings. You agreed on their inclusion and phrasing as questions, so they may **only require minor revisions**.
- **Indicators in red** were also agreed upon for inclusion in the prototype but **require further attention**, as there was insufficient time during the meetings to finalize their phrasing.

Social housing (alone vs pair vs group) (minor revision only)
Please select the option that best describes your parrot's social contact with other parrots

Answers are not ranked according to the risk of poor welfare, as there is no universally better option
Choose one of the following answers

- My parrot lives alone and does not have any type of contact with other parrots
- My parrot is housed individually, but is able to see other parrots and can vocally interact with them
- My parrot can physically interact with one or more parrots at certain times of the day (or week), but is not continuously housed together with other birds
- My parrot is housed together with one parrot and can continuously interact with this bird
- My parrot is housed together with two or more other parrots and can continuously interact with these birds

Please enter your comment here:

Social behaviours
How frequently do these behaviors directed towards other parrots occur?

The answer options for the first 3 types of behaviour range from the best conditions on the left (never) to the worst condition on the right (always). For the last 2 behaviours it is the opposite.

	I don't know	Never	Rarely	Sometimes	Often	Always
Attacks (biting, lunging) other parrots						
Stalking (i.e. following or watching insistently) another parrot						
Regurgitating food for another parrot						
Preening another parrot						
Vocal interaction with another parrot						

Comment box for:
Social behaviours

Reproductive behaviours

How frequently do these behaviors occur?

The answer options range from the best conditions on the left to the worst conditions on the right (excluding the option i don't know)

	I don't know	Never	Rarely	Sometimes	Often	Always
Rubbing its cloaca (vent area) against objects such as perches, cage bars, toys	○	○	○	○	○	○
Actively seeking or spending time in dark, enclosed, or secluded spaces such us underneath furniture, inside drawers or cabinets, behind cushions, or in boxes or clothing.	○	○	○	○	○	○
Lunging, biting, vocalizing aggressively, or chasing any-one who approaches or gets too close to a specific area (e.g. cage or nest)	○	○	○	○	○	○
Egg laying	○	○	○	○	○	○

Comment box for:

Reproductive behaviours

Is there any essential information missing from this section, or any non-essential information that could be removed?

If so, please provide your input in the comment box.

Section 7: Parrot-Human interactions

Each question is accompanied by a comment box where you can provide your feedback and suggestions. For questions presented in table format, a comment box is available directly below the table. Please note the following:

- **Indicators in green** were thoroughly discussed during the meetings. You agreed on their inclusion and phrasing as questions, so they may **only require minor revisions**.
- **Indicators in red** were also agreed upon for inclusion in the prototype but **require further attention**, as there was insufficient time during the meetings to finalize their phrasing.

Rearing history (minor revisions only)

Please select the option that best describes your parrot's rearing history.

The answer options range from the worst conditions at the top to the best conditions at the bottom

Choose one of the following answers

- ○ Unknown
- ○ Wild-caught
- ○ Hand-reared without siblings
- ○ Hand-reared with siblings or socialized with other parrots as soon as weaned
- ○ Initially parent-reared and later hand-raised
- ○ Parent-reared
- ○ Parent-reared and briefly socialized with humans for short amounts of time each week

Please enter your comment here:

Types of interaction with humans

Please indicate if these interactions take place.

	Never	Rarely	Sometimes	Often	Always
Sitting on the shoulder, lap, or elsewhere on me/the caregiver.	○	○	○	○	○
Caregiver is preened by the parrot	○	○	○	○	○
Crawling underneath my clothes or blankets	○	○	○	○	○
Petting the bird's head, cheeks and/or neck	○	○	○	○	○
Petting the bird's chest	○	○	○	○	○
Petting the bird's back and tail	○	○	○	○	○
Holding and shaking the bird's beak while playing	○	○	○	○	○
Respond verbally to the bird's vocalizations	○	○	○	○	○
Kissing the parrot	○	○	○	○	○
Providing food by mouth or allowing the parrot to eat from the mouth	○	○	○	○	○

Comment box for:

Types of interaction with humans

Human-directed behaviours

How frequently do these behaviors directed toward humans occur?

The answer options for the first 4 types of behaviour range from the best condition on the left (never) to the worst condition on the left (always). For the last 2 behaviours is it the opposite

	I don't know	Never	Rarely	Sometimes	Often	Always
Begging for food (raise its wings, flutter them and bob its head up, and down in a rhythmic pattern)	○	○	○	○	○	○
Regurgitating food	○	○	○	○	○	○
Masturbation (rubbing the cloaca against humans)	○	○	○	○	○	○
Contact calls/vocalizations	○	○	○	○	○	○
Bowing and bobbing the body	○	○	○	○	○	○
Crouching with the head down	○	○	○	○	○	○

Comment box for:

Human-directed behaviours

Response upon contact with caregiver

How does your parrot respond to you as the caregiver?

❓ The answer options range from the worst conditions at the top to the best conditions at the bottom
❗ Choose one of the following answers

- ○ My parrot (almost) always lunges, attempts to bite, chases me or tries to escape and/or avoid contact by withdrawing as soon as I am in sight or approaching it.
- ○ My parrot remains seated when I am approaching it, as long as I do not initiate physical contact. If I attempt to initiate contact, it will mostly lunge, attempt to bite, chase me, or try to escape and avoid contact.
- ○ My parrot normally accepts my presence near the enclosure or stand. It does not actively approach me but will accept physical contact that I initiate though it will also regularly lunge, attempt to bite, chase me, or try to withdraw from my attention when I try to interact.
- ○ My parrot (almost) always accepts my presence near the enclosure or stand. It readily accepts physical contact initiated by me and will also regularly initiate physical contact itself by actively approaching me. I rarely observe lunging, attempting to bite, chasing or withdrawal; these will mostly/only occur when I touch specific body parts, make fast movements with my hands, or pet the parrot heavily.

Please enter your comment here:

Response upon contact with familiar person

How does your parrot respond to a familiar person (e.g. partner, family member, friend)?

❓ The answer options range from the worst conditions at the top to the best conditions at the bottom
❗ Choose one of the following answers

- ○ My parrot (almost) always lunges, attempts to bite, chases them, or tries to escape and/or avoid contact by withdrawing as soon as they are in sight or approaching it.
- ○ My parrot remains seated when they are approaching it, as long as they do not initiate physical contact. If they attempt to initiate contact, it will mostly lunge, attempt to bite, chase them, or try to escape and avoid contact.
- ○ My parrot normally accepts their presence near the enclosure or stand. It does not actively approach them but will accept physical contact that they initiate though it will also regularly lunge, attempt to bite, chase them, or try to withdraw from their attention when they try to interact.
- ○ My parrot (almost) always accepts their presence near the enclosure or stand. It readily accepts physical contact initiated by them and will also regularly initiate physical contact itself by actively approaching them. Lunging, attempting to bite, chasing or withdrawal are rare; these will mostly/only occur when they touch specific body parts, make fast movements with their hands, or pet the parrot heavily.

Please enter your comment here:

Time spent in presence of humans

How many hours per day is your parrot surrounded by you and/or familiar people (e.g. partner, family member)?

❓ The answer options range from the worst conditions at the top to the best conditions at the bottom
❗ Choose one of the following answers

- ○ <1h
- ○ It varies greatly per day
- ○ 2h – 4h
- ○ 4h - 8h
- ○ > 8h

Please enter your comment here:

Frequency and duration of manual restraint

How often do you catch and/or restrain your parrot, i.e. grabbing it with a towel or net, holding its body in your hands (with or without gloves or a towel)?

❓ The answer options range from the worst conditions at the top to the best conditions at the bottom
❗ Choose one of the following answers

- ○ Daily, my parrot never steps voluntarily up on my hand/arm or a perch that is offered.
- ○ On a weekly basis, my parrot frequently does not step up onto my hand/arm or a perch that is offered.
- ○ Rarely and only in specific contexts, such as for a veterinary checkup. I generally do not need to restrain my parrot; if the parrot does not step up voluntarily, I can generally push it so it will step up onto my arm/hand or a perch.
- ○ Rarely, if at all, and only in specific contexts, such as for a veterinary checkup. My parrot voluntarily accepts being handled and readily steps up onto my hand/arm

Please enter your comment here:

Food acceptance

How often does your parrot accept food from your hand when you offer it?

❓ The answer options range from the worst conditions at the top to the best conditions at the bottom
❗ Choose one of the following answers

- ○ Never
- ○ Rarely
- ○ Occasionally
- ○ Most of the times

Please enter your comment here:

Comfort behaviour around humans

Does your parrot eat, drink, rest and clean its feathers...

	I don't know	Yes	No
in presence of a familiar person (e.g. partner, family member, friend)?	○	○	○
in your presence?	○	○	○

Comment box for:

Comfort behaviour around humans

Is there any essential information missing from this section, or any non-essential information that could be removed?

If so, please provide your input in the comment box.

Section 8: Abnormal and fear-related behaviours

Each question is accompanied by a comment box where you can provide your feedback and suggestions. For questions presented in table format, a comment box is available

directly below the table. Please note the following:

• **Indicators in green** were thoroughly discussed during the meetings. You agreed on their inclusion and phrasing as questions, so they may **only require minor revisions**.
• **Indicators in red** were also agreed upon for inclusion in the prototype but **require further attention**, as there was insufficient time during the meetings to finalize their phrasing.

Disruptive vocalization or screams (minor revisons only)

How often does your parrot produce disruptive, loud vocalizations or screams?

The answer options range from the worst conditions at the top to the best conditions at the bottom
Choose one of the following answers

- For the majority of the day, and sometimes incessantly for hours with no apparent reason or cause
- Frequently, and incessant screaming can occur but mostly in specific contexts (e.g. when left alone, presence of unfamiliar people/guests)
- Occasionally, which can still be at several times during the day, but never incessantly and mostly when exposed to specific contexts (e.g. when left alone, presence of unfamiliar people/guests)
- Rarely, but never incessantly, and mostly limited to specific moments of the day (e.g. morning and evening) or in response to specific and uncommon situations (e.g. sudden loud noise)
- Never

Please enter your comment here:

Expression of avoidance and escape behaviours (minor revisions only)

How often does your parrot display any of the following behaviors: tremors or shivering, freezing, hiding, withdrawing, attempting to escape by flying or moving away, possibly falling off the perch, or screeching/high-pitched screams?

The answer options range from the worst conditions at the top to the best conditions at the bottom
Choose one of the following answers

- Most of the time, even when in its daily environment
- Regularly, though predominantly when exposed to changes in its daily environment (e.g. presence or approach by new, unfamiliar people/guests or animals, change of furniture, new house decorations, provision of new toys)
- Occasionally, and mostly when exposed to a novel situation, outside of its daily environment (e.g. outdoor activity, visit of a new place or to the vet)
- Rarely, and mostly in response to specific and uncommon situations (e.g. sudden loud noise)
- Never

Please enter your comment here:

Abnormal, sham and excessive behaviours

How frequently do these behaviors occur?

The answer options range from the best condition on the left (never) to the worst condition on the right (always).

	I don't know	Never	Rarely	Sometimes	Often	Always
Pacing: repetitive walking back and forth along a fixed path						
Route tracing: repeatedly follow the same path or pattern within its enclosure, such as moving along a specific perch, climbing the same section of the cage, or flying in a predictable loop.						
Swaying and rocking: repeatedly shift its body side to side or back and forth in a rhythmic motion						
Tongue rolling and flicking: repetitive quick movement of the tongue or rolling or flicking of the tongue inside or outside the beak.						
Beak clacking: rapid and repetitive clicking or clacking of the beak.						
Beak rubbing: repeatedly rubbing the beak against surfaces such as perches or bars						
Repetitive licking: repeated licking of surfaces such as cage bars, perches, or walls						
Spot pecking: repetitively peck at a specific spot, such as a cage bar, perch, wall, or even an imaginary point						
Sham bathing: mimic bathing motions without the presence of water or an appropriate bathing substrate.						
Sham flying: flap its wings repetitively without actually taking flight.						
Sham chewing and chewing not chewable items: mimics chewing motions or engages with non-chewable objects (e.g., metal bars or plastic) or surfaces						
Toe-nail biting: repeatedly bite or nibble at their own toes or nails						

Comment box for:

Abnormal, sham and excessive behaviours

Is there any essential information missing from this section, or any non-essential information that could be removed?

If so, please provide your input in the comment box.

Sections Organization

In this final section of the survey you can share your feedback on the overall structure of the prototype .

You can indicate whether you agree with the current order of the sections, suggest changes to the sequence, or propose moving specific indicators or welfare dimensions to different sections.

Section order

In the left column, you'll find the current order of the prototype sections. If you'd like to suggest a different arrangement, please reorganize the sections in the order you believe is more appropriate using the right column.

Double-click or drag-and-drop items in the left list to move them to the right - your highest ranking item should be on the top right, moving through to your lowest ranking item.
Please select at most 8 answers

Current order

- General information
- Body measurements
- Housing and physical activity
- Provision of enrichment and exploration
- Nutrition and maintenance behaviours
- Social needs, social and reproductive behaviours

Your choices

Parrot-Human interactions

Abnormal and fear-related behaviours

Reallocation of Indicators or Welfare Dimensions

Are there any indicators or welfare dimensions (e.g., exploration, housing, maintenance behaviors) in the sections that you think should be moved to a different section? If so, please specify the indicator(s) and/or welfare dimension(s) in the comment box and indicate the section where they should be relocated.

Sections of the prototype

1:General information - 2: Body measurements - 3: Housing and physical activity 4: Provision of enrichment and exloration - 5: Nutrition and maintenance behaviour - 6: Social needs, social and reproductive behaviours - 7: Parrot-human intteractions - 8: Abnormal and fear-related behaviours

E.g. welfare dimension physical activity should be relocated to section 4: Provision of enrichment and exploration

Submit

vetmeduni

Resume later

Prototype Parrot Welfare Assessment Tool: refined

Information and Consent

vetmeduni University of Veterinary Medicine, Vienna | Utrecht University

Dear expert,
Thank you for taking part in this survey!
The aim of this survey is to review the refined prototype of the welfare assessment tool, developed based on the results of the focus group meetings, in terms of its completeness and clarity.

The indicators are organized into different welfare dimensions, distributed across 8 sections, with the section names and order presented according to the suggestions you provided in the previous meetings. The information is presented in the same structure in which it will be shown to the owners.
Each question is accompanied by a comment box where you can provide your feedback and suggestions. For questions presented in table format, a comment box is available directly below the table.

Please note the following:
•**Indicators in green** were thoroughly discussed during the meetings, and their inclusion and phrasing as questions were agreed upon. No further attention is required.
•Indicators in orange were also thoroughly discussed, with only minor refinements and rewording suggested. They may require **minimal revision**.
•**Indicators in red** were also agreed upon for inclusion in the prototype but **require further attention**, as according to your feedback, they needed modifications in phrasing and format.

You have the **opportunity to save partially finished surveys** by clicking on the top right button saying "Resume later" that you can find in all pages of the survey. To open the partially saved survey, click on the link to the survey that you received by email.

Please note that:

- Participation is voluntary and anonymous.
- You can withdraw at any moment of the study.
- You have to be at least 18 years old to participate.
- The results of this survey will be included in a PhD thesis and published in a scientific journal.
- Your name will not appear in any published documents related to this study, and your statements will remain anonymous.
- Your personal data will be treated in compliance with the European General Data Protection Regulation and will be stored only as long as needed for the purpose of this study.

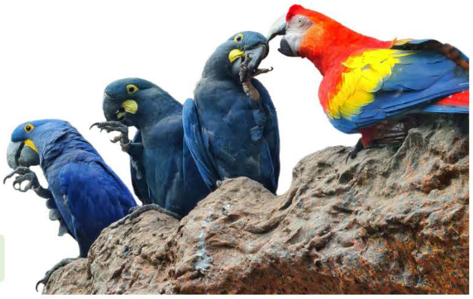

If you have any questions or concerns, please do not hesitate to contact the responsible person of this study:

Andrea Piseddu
Centre for animal Nutrition and Welfare, University of Veterinary Medicine Vienna, Vienna, Veterinaerplatz 1, 1210 Vienna
Andrea.Piseddu@vetmeduni.ac.at

By clicking the button "**Next**" and submitting your responses to the survey you are confirming to have read and understood the above statements and give your informed consent.

There are 88 questions in this survey.

Section 1: General information

Each question is accompanied by a comment box where you can provide your feedback and suggestions. For questions presented in table format, a comment box is available directly below the table. Please note the following:

- **Indicators in green** were thoroughly discussed during the meetings, and their inclusion and phrasing as questions were agreed upon. No further attention is required.
- **Indicators in orange** were also thoroughly discussed, with only minor refinements and rewording suggested. **They may require minimal revision**.
- **Indicators in red** were also agreed upon for inclusion in the prototype but **require further attention**, as according to your feedback, they needed modifications in phrasing and format.

Name of your parrot

Species of your parrot

Sex

Choose one of the following answers

- Female
- Male
- Unknown

How old is your parrot?

How long have you been living with your parrot?

Where did you get your parrot from?

Choose one of the following answers

- Pet store
- Breeder
- Organization that helps re-home parrots
- Private person
- Other:

Rearing history

Please select the option that best describes your parrot's rearing history.

Choose one of the following answers

- Parent-reared and briefly socialized with humans for short amounts of time each week
- Parent-reared
- Initially parent-reared and later hand-raised
- Hand-reared with siblings or socialized with other parrots as soon as weaned
- Hand-reared without siblings
- Wild-caught
- Unknown

Health check up

How often do you take your parrot to a veterinarian for general health check-up?

Choose one of the following answers

- ◯ Two or more times per year
- ◯ Once per year
- ◯ Less than once per year
- ◯ Never went for a general check-up

Please enter your comment here:

Is the veterinarian who examines your parrot specialized in avian medicine?

Choose one of the following answers

- ◯ I don't know
- ◯ Yes
- ◯ No

Please enter your comment here:

How often do you have the opportunity to observe your parrot's behavior?

Choose one of the following answers

- ◯ Several times (4+) throughout the day
- ◯ 2-3 times per day
- ◯ Once a day
- ◯ A few times per week
- ◯ Once a week or less

Please enter your comment here:

Section 2: Physical health

Each question is accompanied by a comment box where you can provide your feedback and suggestions. For questions presented in table format, a comment box is available directly below the table. Please note the following:

- **Indicators in green** were thoroughly discussed during the meetings, and their inclusion and phrasing as questions were agreed upon. No further attention is required.
- **Indicators in orange** were also thoroughly discussed, with only minor refinements and rewording suggested. **They may require minimal revision**.
- **Indicators in red** were also agreed upon for inclusion in the prototype but **require further attention**, as according to your feedback, they needed modifications in phrasing and format.

Condition of flight and body feathers

What is the condition of your parrot's plumage?

Choose one of the following answers

- ◯ The plumage is intact, there are no obvious signs of feather damage or plucking
- ◯ The plumage is mildly damaged or plucked; coverts and/or down feathers are missing in focal areas, but most of the feathers are still intact (though some of these may show signs of damage)
- ◯ The plumage is moderately damaged or plucked; coverts and/or down feathers are missing in several areas of the body leaving a patchy distribution or coverts are missing but down is still mostly or completely present
- ◯ The plumage is severely damaged or plucked; my bird is (almost) completely devoid of its feathers

Skin damage (pop up question for Condition of flight and body feathers)

Do you observe skin damage?

Choose one of the following answers

- ◯ Yes
- ◯ No

Please enter your comment here:

Number and appearance of droppings

Which statement best describes the droppings of your parrot?

Choose one of the following answers

- ◯ My parrot's droppings are well-formed and of normal colour, with a chalky white urate portion and minimal odour. The number of droppings are within my bird's normal elimination pattern.
- ◯ My parrot's droppings are mostly within the normal range, though some occasional or slight changes in colour, odour, consistency or number can be observed.
- ◯ My parrot's droppings are excessive in number, and/or are discoloured, excessively watery and/or contain undigested food particles.
- ◯ My parrot's droppings are scant in number and/or volume, and contain fresh blood or have a tarry black colour.
- ◯ I don't check droppings.

Please enter your comment here:

Pectoral muscle condition score

Please select one the following score according to the muscle condition of your parrot.

HOW TO CHECK YOUR BIRDS SHAPE

- Getting hands on is key. Not all birds are used to being handled but it is difficult to judge if your bird is the right weight by sight. You will need to gently feel your bird, using restraint if necessary.
- Use bare hands and not gloves to handle birds as then you can judge the tightness of grip. If you need to protect yourself use a cloth or towel.
- Small birds can be held in one hand with the neck between the first and second finger and the bird's back against the palm so that the wings and body are gently restrained in the closed hand.
- Larger parrots may take two people, one to hold the bird and the other to assess its body condition. A towel or cloth is used over the open hand to grasp the bird firmly behind its head and neck. The towel is then wrapped around the wings and body to prevent flapping. Gently stroking the top of the head and talking to the bird gently will help to calm it.
- Gently run your fingertips down the centre of the front of the bird in the midline over the breast area. You should be able to feel a bony ridge (known as the keel or breast bone). This should be easy to feel but not too prominent.
- Next, run your fingers at right angles to the keel across the breast muscles. If these feel shrunken so that the keel sticks out prominently your bird is too thin. If the breast muscles are just rounded but you can still feel the keel your bird is in good condition. If you cannot feel the keel and the muscles are very rounded or you can feel or see fat moving underneath the skin your bird is overweight.
- The breast muscle can also vary in size depending on how much exercise your bird gets so if it flies a lot it will have larger firmer breast muscles than a bird who does not fly. However, the same criteria still apply in assessing body condition prominence of the bony keel and presence of fat underneath the skin.

For more information about the score please consult this link: https://www.ukpetfood.org/spotlight-on-obesity/how-to-identify-if-a-pet-needs-help/bird-size-o-meter-new.html

Choose one of the following answers

- ◯ I can't calculate the score because my bird does not accept to be handled.
- ◯ 1

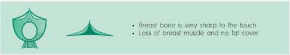

- ◯ 2

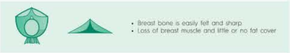

- ◯ 3

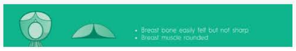

Please enter your comment here:

4

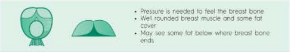

5

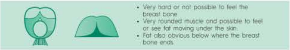

Signs of illness

Do you notice any of these conditions?

	Yes	No	I am not sure
Overgrown beak and/or nails 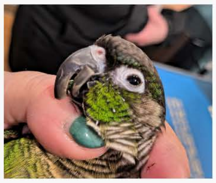			
Upper and lower beak not properly aligned 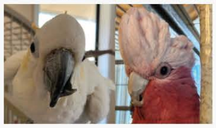			
Redness, swelling or loss of feathers around eyes			
Crusty material in or around nares			
Flakiness on skin or beak			
**Feather abnormalities** 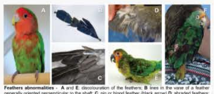 **Feathers abnormalities** - **A** and **E**: discolouration of the feathers; **B** lines in the vane of a feather generally oriented perpendicular to the shaft; **C**: pin or blood feather (black arrow) **D**: abraded feathers; **F**: feather discolouration and depigmentation, overall dull- and ragged looking plumage. From: Plumage disorders in psittacine birds - part 1: feather abnormalities. van Zeeland, Y.R.A.; Schoemaker, N.J. (2014).			
Lameness or shifting of body weight			
Blood loss or injury			
Discharge from nares (nostrils), eyes, or mouth			
Labored breathing or abnormal respiratory sounds			
Enlargement or swelling on the body			
Vomiting or regurgitation			
Fluffed posture and sleeping more than normal			
Inability to perch			
>10% increase or decrease of body weight within a week			
Swelling, ulcers, or lesions under the feet			
Cloaca protrudes outside the body (prolapse)			

Comment box for: **Signs of illness**

Vet diagnosis (follow up question for Signs of illness)

Were the condition(s) listed above diagnosed by a veterinarian?

Choose one of the following answers

- Yes, all conditions
- Only some conditions
- No

Please enter your comment here:

Treatments for (follow up questions for Signs of Illness)

Is your parrot currently receiving medication prescribed by a veterinarian for any of the conditions listed above?

Choose one of the following answers

- Yes, my parrot is receiving treatments prescribed by the veterinarian
- No, but my parrot is receiving over-the-counter medications
- No, my parrot is not receiving any medication

Please enter your comment here:

Section 3: Housing and physical activity

Each question is accompanied by a comment box where you can provide your feedback and suggestions. For questions presented in table format, a comment box is available directly below the table. Please note the following:

• **Indicators in green** were thoroughly discussed during the meetings, and their inclusion and phrasing as questions were agreed upon. No further attention is required.
• **Indicators in orange** were also thoroughly discussed, with only minor refinements and rewording suggested. **They may require minimal revision**.
• **Indicators in red** were also agreed upon for inclusion in the prototype but **require further attention**, as according to your feedback, they needed modifications in phrasing and format.

Enclosure type

Where is your parrot's enclosure located?

If your parrot has multiple enclosures, please specify all locations

Enclosure: space where the parrot sleeps, eats, drinks, and stays when it can't be supervised

	Main enclosure (space where the parrot stays most of time)	Secondary enclosure (additional space used occasionally)	Not provided
Living room			
Hallway			
Kitchen			
Bedroom			
Garage			
Basement			
Parrot lives indoor and does not have a dedicated enclosure			
Room exclusively dedicated to the parrot			
Outdoor enclosure			

Comment box for **Enclosure type**

Enclosure size

Which statement most accurately describes the size of your parrot's main enclosure (where it resides most of the daytime) and its ability to move around?

Choose one of the following answers

- My parrot has ample space to move, hop, climb and fly around
- My parrot has at least 1 wingspan of space in all directions to move, climb, and hop
- My parrot can fully extend its wings, but only in one direction, with limited space for movement, climbing, and hopping
- My parrot is unable to fully extend its wings in any direction, with limited space to move

Please enter your comment here:

Enclosure material

What type of material is your parrot's enclosure made from?

Check all that apply

- I don't know
- Stainless steel
- Powder-coated metal
- Wrought iron
- Plastic-coated wire, zinc, wood or acrylic
- Other:

Bars orientation

Please select the statement(s) that most accurately describe the bar orientation of your parrot's enclosure(s).

Check all that apply

- The enclosure(s) has grid-patterned bars
- The enclosure(s) has vertical bars
- The enclosure(s) has horizontal bars
- There are no bars to allow climbing (e.g. solid panels made of glass, acrylic)

Number of perches

How many perches are available in the parrot's enclosure(s)?

Choose one of the following answers

- > 5
- 3 – 5
- 1 – 2
- There are no perches

Perches' material

Did you check if the material of the perches is safe?

Choose one of the following answers

- Yes
- No

Please enter your comment here:

Perches' diameter

What sizes of perch diameters do you provide for your parrot?

Choose one of the following answers

- A wide variety of sizes ranging from very thin to very thick
- A few sizes (e.g., one small, one medium, and one large)
- Only one size

Please enter your comment here:

Provision of opportunities that allow movement and climbing

Please indicate how often your parrot receives the following types of enrichment that promote movement and climbing.

	Every day	4–6 days per week	1–3 days per week	Few times per month or less	Never
Ropes					
Swings					
Ladders					
Boings					

Comment box for: **Provision of opportunities that allow movement and climbing**

Opportunity to spent time outside the enclosure

How often does your parrot spend time out of its enclosure ?

Choose one of the following answers

- Most or all of its time (e.g. only in the enclosure when sleeping or when there is no human supervision)
- Every day, for 3 hours or more
- Every day, for 3 hours or less
- Several times a week, but not every day
- Never or less than once a week on average

Presence of a retreating area/room to rest, sleep or withdraw

Does your parrot have unlimited access to an undisturbed area where it can rest, sleep or retreat from potential stressful situations (presence of unfamiliar people, other animals etc.)?

Choose one of the following answers

- Yes
- No

Hygiene: frequency of cleaning food and water bowls, foraging toys, enclosure

How often do you clean the...

	Everyday or after every use	Several times a week, but not every day	Once a week or less
Enclosure (cage, room, aviary)			
Food bowl			
Water bowl			
Foraging toys			

Climate (temperature. humidity)

Do you regularly check the humidity and temperature to ensure they are appropriate for your parrot's species?

Choose one of the following answers

- Yes, both
- Only temperature
- Only humidity
- No

Please enter your comment here:

Air quality

How often do you refresh the air in the area where your parrot lives and spends most of its time?

Choose one of the following answers

- More than once per day / I use an air purifier / My parrot lives outdoor
- Once per day / I use an air purifier but is not constantly activated
- Several times a week, but not every day
- Once per week or less

Access to outdoor spaces

How often does your parrot spend time outdoor, if environmental circumstances permit (no risk of predation or mosquitoes' bites, appropriate temperature)?

Choose one of the following answers

- At least once a day
- At least once a week
- Less than once a week on average
- Never

Exposure to direct sunlight/UV light

How often do you expose your parrot to a UVA and UVB lamp or direct sunlight without a window in between?

Choose one of the following answers

- Everyday
- Several times a week, but not every day
- Once per week or less
- Never

Please enter your comment here:

Opportunity to spend time in high positions

Does your parrot have the opportunity to perch in high locations that are out of reach of people and other animals?

Choose one of the following answers

- Yes, both inside and outside of the enclosure
- Only inside the enclosure
- Only outside the enclosure
- No

Please enter your comment here:

Ability to fly

How would you describe your parrot's flight ability?

If you are unsure of your parrot's flying ability, please select the answer "I don't know." Avoid attempting to assess it, as it could lead to injury or stress for your bird.

Choose one of the following answers

- I don't know
- My parrot can fly in all directions, including downward, horizontally and upward, with full flight capability
- My parrot can fly downward and horizontally but has limited ability to gain altitude
- My parrot is able to fly downward and horizontally but cannot gain altitude
- My parrot can only fly downward
- My parrot is unable to fly in any direction.

Please enter your comment here:

Wing trim (pop up question for ability to fly)

Has your parrot received a wing trim?

Please note that wing trimming is illegal in some countries

Choose one of the following answers

- No
- Yes, my bird has received a bilateral skinny wing trim
- [illegible]

Please enter your comment here:

○ Yes, my bird has received a bilateral, transverse wing trim

○ Yes, my bird has received a bilateral wing trim which includes the primaries and secondaries

○ Yes, my bird has received a unilateral wing trim

○ Yes, my bird has been permanently deflighted through a surgical procedure (e.g., pinioning)

Opportunity to fly

How often does your parrot have the opportunity to fly in a safe space?

Choose one of the following answers

○ Every day for 4 or more hours

○ Every day for less than 4 hours

○ Several times a week, but not every day

○ Never or less than once a week on average

Level of activity

How much time does your parrot spend each day moving, climbing, and flying?

Choose one of the following answers

○ I don't know

○ 1 hour or less

○ Between 1 and 2 hours

○ Between 2 and 4 hours

○ 4 or more hours

Section 4: Provision of enrichment and exploration

Each question is accompanied by a comment box where you can provide your feedback and suggestions. For questions presented in table format, a comment box is available directly below the table. Please note the following:

- **Indicators in green** were thoroughly discussed during the meetings, and their inclusion and phrasing as questions were agreed upon. No further attention is required.
- **Indicators in orange** were also thoroughly discussed, with only minor refinements and rewording suggested. **They may require minimal revision**.
- **Indicators in red** were also agreed upon for inclusion in the prototype but **require further attention**, as according to your feedback, they needed modifications in phrasing and format.

Provision of foraging and cognitive enrichment

How often do you provide the following types of enrichment?

	Every day	4–6 days per week	1–3 days per week	Few times per month or less	Never
Certified chewable toys, cardboard or paper without ink, natural, not toxic and untreated cork and branches that can be safely chewed and destroyed	○	○	○	○	○
Puzzles	○	○	○	○	○
Problem solving games	○	○	○	○	○
Multiple food stations	○	○	○	○	○
Scatter feeding (e.g. foraging mat, spreading food out in various locations)	○	○	○	○	○
Foraging tray or box (food mixed with inedible items)	○	○	○	○	○
Larger chunks of food or whole food items	○	○	○	○	○
Commercial puzzle feeders/foraging toys	○	○	○	○	○
DIY (do it yourself) destructible foraging	○	○	○	○	○
Interactive toys that make sounds and/or move	○	○	○	○	○
Auditory enrichment (e.g. radio, music)	○	○	○	○	○
Visual enrichment (e.g. tv or tablets)	○	○	○	○	○

Comment box for: **Provision of foraging and cognitive enrichment**

Interaction with enrichment

How does your parrot interact with the enrichment provided?

Types of interaction: manipulation with beak and/or feet (e.g., chewing, grasping, exploring) or reactions to sound and visual cues.

	I don't know	Frequently and repeatedly uses with the enrichment, exploring and manipulating it extensively with the beak and/or feet, showing continued interest until the item is destroyed (in case of destructible items) or food/contents is removed (if applicable)	Occasionally uses or interacts with the enrichment; while the bird shows some interest in the enrichment at times, it ignores the enrichment at other times	Seldom uses or interacts with the enrichment; interactions are generally for brief periods only, and rarely more than once a day	Does not use or interact with the enrichment at all, and completely ignores it
Certified chewable toys, carboard or paper without ink, natural, not toxic and untreated cork and branches that can be safely chewed and destroyed	○	○	○	○	○
Puzzles	○	○	○	○	○
Problem solving games	○	○	○	○	○
Multiple food stations	○	○	○	○	○
Scatter feeding (e.g. foraging mat, spreading food out in various locations)	○	○	○	○	○
Foraging tray or box (food mixed with inedible items)	○	○	○	○	○
	**I don't know**	**Frequently and repeatedly uses with the enrichment, exploring and manipulating it extensively with the beak and/or feet, showing continued interest until the item is destroyed (in case of destructible items) or food/contents is removed (if applicable)**	**Occasionally uses or interacts with the enrichment; while the bird shows some interest in the enrichment at times, it ignores the enrichment at other times**	**Seldom uses or interacts with the enrichment; interactions are generally for brief periods only, and rarely more than once a day**	**Does not use or interact with the enrichment at all, and completely ignores it**
Larger chunks of food or whole food items	○	○	○	○	○
Commercial puzzle feeders/foraging toys	○	○	○	○	○
DIY (do it yourself) destructible foraging	○	○	○	○	○

[illegible] foraging					
Interactive toys that make sounds and/or move	○	○	○	○	○
Auditory enrichment (e.g. radio, music)	○	○	○	○	○
Visual enrichment (e.g. tv or tablets)	○	○	○	○	○

Comment box: **Interaction with enrichment**

Provision of foraging enrichment in relation to the daily food ration

How is foraging enrichment provided for your parrot?

Foraging enrichment includes toys, devices, or other opportunities that encourage natural behaviors such as searching, procuring, and extracting food.

Choose one of the following answers

- For treats and more than half of the daily food ration
- For treats and about half of the daily food ration
- For treats and a small portion of of the daily food ration
- Only for treats

Please enter your comment here:

Time spent foraging

How much time does your parrot spend daily foraging (i.e., searching for, procuring, and extracting food from toys that you provide)?

Choose one of the following answers

- I don't know
- 4 hours or more
- Between 3 and 4 hours
- Between 1 and 3 hours
- Up to 1 hour
- My parrot does not forage

Opportunities to select items based on preference (e.g., for colour, shape or type of material) (minor revisions only)

Do you select toys based on your parrot's preference for shape, size and colours?

Choose one of the following answers

- Yes
- No

Please enter your comment here:

Enrichment replacement

How often do you replace toys/chewable items/ climbing enrichment by introducing new different one/s?

Choose one of the following answers

- Every day
- 4–6 days per week
- 1–3 days per week
- Few times per month or less
- Never

Please enter your comment here:

Response to novel objects

How does your parrot generally react towards unfamiliar objects (e.g. new toys, new home decoration) in its proximity?

Choose one of the following answers

- My bird actively approaches the object touching, chewing and manipulating it; certain objects may still trigger freezing or withdrawing responses.
- My bird appears interested in the object, and cautiously approach it, after initially being reluctant to do so; certain objects (e.g. larger furniture or electronic household devices) may still trigger freezing or withdrawing responses.
- My bird withdraws or freezes to most objects; certain objects (e.g. larger toys, larger furniture or electronic household devices) may trigger more freezing or withdrawing responses.
- My bird shows one or more of these behaviours: excessive or incessant screaming, frantic movements, flying away, falling of the perch. This response happens with any type of unfamiliar object.

Alertness

What is your parrot's current alertness level?

Choose one of the following answers

- My parrot is very active and curious. It eagerly explores new objects, interacts frequently with people and toys, and responds quickly to its surroundings
- My parrot responds to sounds, movements, and interactions. It engages with toys, interacts with people, and explores its environment with moderate enthusiasm
- My parrot reacts occasionally but needs encouragement to engage. It shows some interest in its surroundings and sometimes participates in activities
- My parrot is mostly inactive or lethargic, shows little interest in its surroundings and does not respond much to things happening around it

Please enter your comment here:

Section 5: Nutrition and maintenance behaviours

Each question is accompanied by a comment box where you can provide your feedback and suggestions. For questions presented in table format, a comment box is available directly below the table. Please note the following:

- **Indicators in green** were thoroughly discussed during the meetings, and their inclusion and phrasing as questions were agreed upon. No further attention is required.
- **Indicators in orange** were also thoroughly discussed, with only minor refinements and rewording suggested. **They may require minimal revision**.
- **Indicators in red** were also agreed upon for inclusion in the prototype but **require further attention**, as according to your feedback, they needed modifications in phrasing and format.

Composition of the diet

Which of the following foods do you provide?

	Main component of the diet	Moderate amount	Small/limited quantities	As treat during training activities or as a reward for desired behaviours	Not provided
Fresh vegetables	○	○	○	○	○
Pellet and other formulated food	○	○	○	○	○

Fresh fruit	○	○	○	○	○
Sprouted seeds	○	○	○	○	○
Eggs	○	○	○	○	○
Legumes	○	○	○	○	○
Grains	○	○	○	○	○
Seed mix	○	○	○	○	○
Nuts	○	○	○	○	○
Other animal-based proteins (e.g. chicken bones)	○	○	○	○	○
Tofu	○	○	○	○	○
Other plant-based proteins	○	○	○	○	○
Nectar	○	○	○	○	○
Processed food specifically designed for human consumption	○	○	○	○	○

Comment box for: **Composition of the diet**

Food Selectivity

Does your parrot eat all types of food that you provide?
Observing the animal while it eats is preferable, but checking the food bowl can also provide valuable insights.

Choose one of the following answers

- ○ I don't know
- ○ Yes
- ○ No

Diet Apropriateness

Have you checked whether the diet you provide is balanced and appropriate for your parrot's species?

Choose one of the following answers

- ○ Yes, I consulted my veterinarian or a behavioural consultant
- ○ Yes, I researched by myself online, in books, or magazines
- ○ No

Availability of fresh and clean water

How often do you change your parrot's water?

Choose one of the following answers

- ○ More than twice a day or as needed throughout the day
- ○ Twice a day
- ○ Once a day
- ○ Less than once per day

Changes in food and/or water consumption

Have you observed a change in appetite or water intake by your parrot in the last 2 weeks?
i.e. you observed these changes directly observing the parrot's behaviour or by checking the amount of food or water consumed.

Choose one of the following answers

- ○ I don't know
- ○ No
- ○ Yes

Daytime spent sleeping / resting

What daytime does your parrot sleep / rest?

Check all that apply

- ☐ I don't know
- ☐ Morning
- ☐ Afternoon
- ☐ Evening
- ☐ Night

Changes of resting-sleeping patterns

Have you noticed any changes in the sleeping pattern of your parrot in the last two weeks?
e.g. sleeping more than usual or at times of the day when it typically does not sleep.

Choose one of the following answers

- ○ I don't know
- ○ No
- ○ Yes

Opportunity to bathe

Please indicate how often you provide the following bathing opportunities for your parrot

	Every day	4–6 days per week	1–3 days per week	Few times per month or less	Never
Mist	○	○	○	○	○
Shower	○	○	○	○	○
Shallow dish or tray	○	○	○	○	○
Other	○	○	○	○	○

Comment box for: **Opportunity to bathe**

Interest in bathing

How does your parrot bathe?

	During every bath	During most baths	During some baths	Rarely	Never
My parrot bathes on its own	○	○	○	○	○
My parrot bathes on its own but needs encouragement to do so	○	○	○	○	○
My parrot tries to avoid bathing, but I make sure it gets bathed anyway	○	○	○	○	○

Comment box for: **Interest in bathing**

Beak maintenance

Does your parrot engage in self-maintenance behaviors for its beak?

e.g. rubbing it against hard objects, grinding it by making a rhythmic motion with the upper and lower mandibles, or using bars or other objects to clean the inside of its beak

Choose one of the following answers

- ○ I don't know
- ○ Yes
- ○ No

Please enter your comment here:

Changes in preening activity

Have you noticed any changes in your parrot's self-preening behavior (such as using its beak to clean, arrange, or maintain its feathers) over the past month?

Choose one of the following answers

- ○ I don't know
- ○ No
- ○ Yes

Please enter your comment here:

Section 6: Social and reproductive behaviours

Each question is accompanied by a comment box where you can provide your feedback and suggestions. For questions presented in table format, a comment box is available directly below the table. Please note the following:

- **Indicators in green** were thoroughly discussed during the meetings, and their inclusion and phrasing as questions were agreed upon. No further attention is required.
- **Indicators in orange** were also thoroughly discussed, with only minor refinements and rewording suggested. **They may require minimal revision**.
- **Indicators in red** were also agreed upon for inclusion in the prototype but **require further attention**, as according to your feedback, they needed modifications in phrasing and format.

Social housing (alone vs pair vs group)

Please select the option that best describes your parrot's social contact with other parrot.

Choose one of the following answers

- ○ My parrot is housed together with two or more other parrots and can continuously interact with these birds
- ○ My parrot is housed together with one parrot and can continuously interact with this bird
- ○ My parrot can physically interact with one or more parrots at certain times of the day (or week), but is not continuously housed together with other birds
- ○ My parrot is housed individually, but is able to see other parrots and can vocally interact with them
- ○ My parrot lives alone and does not have any type of contact with other parrots

Social behaviours

How frequently do these social interactions occur?

	On every encounter	On most encounters	On some encounters	Rarely	Never
Staying right beside another parrot	○	○	○	○	○
Feeding alongside another parrot	○	○	○	○	○
Preening another parrot	○	○	○	○	○
Being preened by another parrot	○	○	○	○	○
Vocal interaction with another parrot (contact calls)	○	○	○	○	○
Mating with another parrot	○	○	○	○	○
Stalking (i.e. following or watching insistently) another parrot	○	○	○	○	○
Stalked (i.e. followed or watched insistently) by another parrot	○	○	○	○	○
Regurgitating food for other parrot	○	○	○	○	○
Having food regurgitated by another parrot	○	○	○	○	○
Attacks (biting, lunging) another parrot	○	○	○	○	○
Being attacked (bitten or lunged at) by another parrot	○	○	○	○	○

Comment box for:

Social behaviours

Reproductive behaviours

How frequently do you observe these behaviors?

	Every day	4–6 days per week	1–3 days per week	Few times per month or less	Never
Rubbing its cloaca (vent area) against objects such as perches, cage bars, toys	○	○	○	○	○
Actively seeking or spending time in dark, enclosed, or secluded spaces such us underneath furniture, inside drawers or cabinets, behind cushions, or in boxes or clothing.	○	○	○	○	○
Lunging, biting, vocalizing aggressively, or chasing any-one who approaches or gets too close to a specific area (e.g. cage or nest)	○	○	○	○	○
Egg laying	○	○	○	○	○
Regurgitating food and directing it towards its own body parts (e.g. its foot)	○	○	○	○	○

Comment box for:

Reproductive behaviours

Section 7: Parrot-Human interactions

Each question is accompanied by a comment box where you can provide your feedback and suggestions. For questions presented in table format, a comment box is available directly below the table. Please note the following:

- **Indicators in green** were thoroughly discussed during the meetings, and their inclusion and phrasing as questions were agreed upon. No further attention is required.
- **Indicators in orange** were also thoroughly discussed, with only minor refinements and rewording suggested. **They may require minimal revision**.
- **Indicators in red** were also agreed upon for inclusion in the prototype but **require further attention**, as according to your feedback, they needed modifications in phrasing and format.

Time spent in presence of humans

How many hours per day is your parrot surrounded by you and/or familiar people (e.g. partner, family member)?

Choose one of the following answers

- It varies greatly per day
- <1h
- 2h – 4h
- 4h - 8h
- > 8h
- All time, including night

Please enter your comment here:

Types of interaction with humans (table 1)

How often do you allow your parrot to engage in the following behaviors?

	Every day	4–6 days per week	1–3 days per week	Few times per month or less	Never
Sitting on your shoulder, lap, or another part of your body					
Gently nibbling or grooming your hair, beard, eyelashes, or skin with its beak (preening)					
Crawling under your clothes or blankets					

Types of interaction with humans (table 2)

How often do you interact with your parrot in the following manners?

	Every day	4–6 days per week	1–3 days per week	Few times per month or less	Never
Petting the bird's head, cheeks and/or neck					
Petting the bird under the wings					
Petting the bird's chest					
Petting the bird's back and tail					
Holding and shaking the bird's beak while playing					
Gently responding to the bird's vocalizations					
Talking to my bird					
Yelling at my bird					
	Every day	4–6 days per week	1–3 days per week	Few times per month or less	Never
Playing music for my bird					
Bringing my bird with me when I go out					
Training my bird					
Kissing my bird					
Providing food by mouth or allowing the bird to eat from the mouth					
Pressing on my parrot's chest to encourage it to step up onto my hand, arm, or an offered perch					
Holding my bird's body in my hands (with or without gloves or a towel)					
Grabbing my bird with a net or a towel					

Comment box for: **Types of interaction with humans**

Parrot response to training (Pop-up question if the owner indicates they train their bird)

How does your parrot respond during training session?

Choose one of the following answers

- Very focused and responds quickly to commands
- Mostly focused, but gets distracted sometimes
- Responds sometimes, but often gets distracted
- Rarely focused, responds only occasionally
- Does not respond or shows no interest in training

Please enter your comment here:

Behaviours directed towards humans

How frequently does your parrot display the behavior below towards you or other household members?

	Every day	4–6 days per week	1–3 days per week	Few times per month or less	Never
Offering the head/neck to be petted					
Contact calls/vocalizations					
Crouching with the head down					
Bowing and bobbing the body					
Voluntarily steps up onto your hand, arm, or an offered perch without encouragement					
Begging for food (raise its wings, flutter them and bob its head up, and down in a rhythmic pattern)					
Regurgitating food					
Masturbation (rubbing the cloaca against humans)					

Comment box for: **Behaviours directed towards humans**

Response upon contact with caregiver (original version)

How does your parrot respond to you as the caregiver?

Choose one of the following answers

- My parrot (almost) always accepts my presence near the enclosure or stand. It readily accepts physical contact initiated by me and will also regularly initiate physical contact itself by actively approaching me. I rarely observe lunging, attempting to bite, chasing or withdrawal; these will mostly/only occur when I touch specific body parts, make fast movements with my hands, or pet the parrot heavily.
- My parrot normally accepts my presence near the enclosure or stand. It does not actively approach me but will accept physical contact that I initiate though it will also regularly lunge, attempt to bite, chase me, or try to withdraw from my attention when I try to interact.
- My parrot remains seated when I am approaching it, as long as I do not initiate physical contact. If I attempt to initiate contact, it will mostly lunge, attempt to bite, chase me, or try to escape and avoid contact.
- My parrot (almost) always lunges, attempts to bite, chases me or tries to escape and/or avoid contact by withdrawing as soon as I am in sight or approaching it.

Please enter your comment here:

Response upon contact with caregiver (new version)

How does your parrot respond to you as the caregiver?

	Every time	Most of the times	Sometimes	Rarely	Never	I don't know
My parrot accepts my presence near the enclosure or stand	○	○	○	○	○	○
My parrot accepts physical contact initiated by me	○	○	○	○	○	○
My parrot initiates physical contact itself by actively approaching me	○	○	○	○	○	○
My parrot remains seated when I am approaching it	○	○	○	○	○	○
My parrot tries to lunge, attempt to bite or chase me when I am approaching it	○	○	○	○	○	○
My parrot tries to escape and avoid contact when I am approaching it	○	○	○	○	○	○

Response upon contact with familiar person (original version)

How does your parrot respond to a familiar person (e.g. partner, family member, friend)?

Choose one of the following answers

- My parrot (almost) always accepts their presence near the enclosure or stand. It readily accepts physical contact initiated by them and will also regularly initiate physical contact itself by actively approaching them. Lunging, attempting to bite, chasing or withdrawal are rare; these will mostly/only occur when they touch specific body parts, make fast movements with their hands, or pet the parrot heavily.
- My parrot normally accepts their presence near the enclosure or stand. It does not actively approach them but will accept physical contact that they initiate though it will also regularly lunge, attempt to bite, chase them, or try to withdraw from their attention when they try to interact.
- My parrot remains seated when they are approaching it, as long as they do not initiate physical contact. If they attempt to initiate contact, it will mostly lunge, attempt to bite, chase them, or try to escape and avoid contact.
- My parrot (almost) always lunges, attempts to bite, chases them, or tries to escape and/or avoid contact by withdrawing as soon as they are in sight or approaching it.

Please enter your comment here:

Response upon contact with familiar person (new version)

How does your parrot respond to a familiar person (e.g. partner, family member, friend)?

	Every time	Most of the times	Sometimes	Rarely	Never	I don't know
My parrot accepts their presence near the enclosure or stand	○	○	○	○	○	○
My parrot accepts physical contact initiated by them	○	○	○	○	○	○
My parrot initiates physical contact itself by actively approaching them	○	○	○	○	○	○
My parrot remains seated when they are approaching it	○	○	○	○	○	○
My parrot tries to lunge, attempt to bite or chase them when they are approaching it	○	○	○	○	○	○
My parrot tries to escape and avoid contact when they are approaching it	○	○	○	○	○	○

Response to caregiver and familiar person

Which versions do you prefer?

Choose one of the following answers

- Original versions
- New versions

Please enter your comment here:

Comfort behaviour around humans

Does your parrot feel comfortable to...

	eat, drink, rest and clean its feathers?			spend time in locations where it can be easily reached by a human?		
	I don't know	Yes	No	I don't know	Yes	No
in your presence	○	○	○	○	○	○
in the presence of all household members	○	○	○	○	○	○
in the presence of a familiar person (e.g. partner, family members, friend)	○	○	○	○	○	○
in the presence of a stranger (e.g., a repair technician, delivery person)	○	○	○	○	○	○

Comment box for: **Comfort behaviour around humans**

Section 8: Abnormal and fear-related behaviours

Each question is accompanied by a comment box where you can provide your feedback and suggestions. For questions presented in table format, a comment box is available directly below the table. Please note the following:

• **Indicators in green** were thoroughly discussed during the meetings, and their inclusion and phrasing as questions were agreed upon. No further attention is required.
• **Indicators in orange** were also thoroughly discussed, with only minor refinements and rewording suggested. **They may require minimal revision**.
• **Indicators in red** were also agreed upon for inclusion in the prototype but **require further attention**, as according to your feedback, they needed modifications in phrasing and format.

Disruptive vocalization or screams

How often does your parrot produce disruptive, loud vocalizations or screams?

The answer options range from the worst conditions at the top to the best conditions at the bottom

Choose one of the following answers

- For the majority of the day, and sometimes incessantly for hours with no apparent reason or cause
- Frequently, and incessant screaming can occur but mostly in specific contexts (e.g. when left alone, presence of unfamiliar people/guests)
- Occasionally, which can still be at several times during the day, but never incessantly and mostly when exposed to specific contexts (e.g. when left alone, presence of unfamiliar people/guests)
- Rarely, but never incessantly, and mostly limited to specific moments of the day (e.g. morning and evening) or in response to specific and uncommon situations (e.g. sudden loud noise)
- Never

Expression of avoidance and escape behaviours

When does your parrot display any of the following behaviors?

The answer options range from the worst conditions at the top to the best conditions at the bottom

	Tremors or shivering, freezing, hiding, withdrawing	Attempting to escape by flying or moving away, possibly falling off the perch, screeching/high-pitched screams
I don't know	○	○
Never	○	○
Mostly in response to specific or uncommon situations (e.g. visit to the vet, sudden loud noise)	○	○
Mostly when exposed to a certain situation, outside of its daily environment (e.g. outdoor activity, visit of a new place)	○	○
Predominantly when exposed to changes in its daily environment (e.g. presence or approach by new, unfamiliar people/guests or animals, change of furniture, new house decorations, provision of new toys)	○	○
Most of the time, even in its daily environment	○	○

Comment box for: **Expression of avoidance and escape behaviours**

Abnormal, sham and excessive behaviours

Does your parrot exhibit any of the following behaviors?

The answer options range from the best condition on the left (never) to the worst condition on the right (always).

	Yes	No	I am not sure
Pacing: repetitive walking back and forth along a fixed path	○	○	○
Route tracing: repeatedly follow the same path or pattern within its enclosure, such as moving along a specific perch, climbing the same section of the cage, or flying in a predictable loop	○	○	○
Swaying and rocking: repeatedly shift its body side to side or back and forth in a rhythmic motion	○	○	○
Tongue rolling and flicking: repetitive quick movement of the tongue or rolling or flicking of the tongue inside or outside the beak	○	○	○
Beak clacking: rapid, repetitive, excessive or compulsive clicking or clacking of the beak	○	○	○
Beak rubbing: repeatedly rubbing the beak against surfaces such as perches or bars	○	○	○
Repetitive licking: repeated licking of surfaces such as cage bars, perches, or walls	○	○	○
	**Yes**	**No**	**I am not sure**
Spot pecking: repetitively peck at a specific spot, such as a cage bar, perch, wall, or even an imaginary point	○	○	○
Sham bathing: mimic bathing motions without the presence of water or an appropriate bathing substrate	○	○	○
Sham flying: repetitively twitches its wings without taking flight (distinct from learned wing flapping)	○	○	○
Sham chewing and chewing not chewable items: mimics chewing motions or engages with non-chewable objects (e.g., metal bars or plastic) or surfaces	○	○	○
Toe-nail biting: repeatedly bite or nibble at their own toes or nails	○	○	○
Feeding objects (e.g. mirrors, toys)	○	○	○

Comment box for:

Abnormal, sham and excessive behaviours

I simplified the scale to include only "Yes," "No," and "I don't know" because I believe it can be challenging for owners to accurately assess how frequently they observe abnormal behaviors. I think asking whether they observe these behaviors is sufficient. In the feedback, we can explain that while frequency is important, this aspect should be addressed and analyzed with a specialist for a more accurate evaluation. Let me know what you think about this change.

Submit

vetmeduni
Veterinärmedizinische Universität Wien

Load unfinished survey Resume later

This survey is currently not active. You will not be able to save your responses.

Prototype Parrot Welfare Assessment Tool: second refinement

There are 71 questions in this survey.

Section 1: General information

This section does not include any questions that were modified based on previous comments or suggestions, as all items were already finalized. If you would still like to share feed-back, please feel free to leave a comment directly in the Word document.

Name of your parrot

Species of your parrot

Sex

Choose one of the following answers

- Female
- Male
- Unknown

How old is your parrot?

How long have you been living with your parrot?

Where did you get your parrot from?

Choose one of the following answers

- Pet store
- Breeder
- Organization that helps re-home parrots
- Private person

Other:

Rearing history

Please select the option that best describes your parrot's rearing history.

Choose one of the following answers

- Parent-reared and briefly socialized with humans for short amounts of time each week
- Parent-reared
- Initially parent-reared and later hand-raised
- Hand-reared with siblings or socialized with other parrots as soon as weaned
- Hand-reared without siblings
- Wild-caught
- Unknown

Health check up

How often do you take your parrot to a veterinarian for general health check-up?

Choose one of the following answers

- Two or more times per year
- Once per year
- Less than once per year
- Never went for a general check-up

Is the veterinarian who examines your parrot specialized in avian medicine?

Choose one of the following answers

- I don't know
- Yes
- No

How often do you have the opportunity to observe your parrot's behavior?

Choose one of the following answers

- Several times (4+) throughout the day
- 2-3 times per day
- Once a day
- A few times per week
- Once a week or less

Section 2: Physical health

Please note the following:

- **Indicators in green** were thoroughly discussed during the meetings, and their inclusion and phrasing as questions were agreed upon. No further attention is required.
- [illegible]

• Indicators in orange were discussed during our last meeting and have since been modified based on that discussion.
• Indicators in purple were revised by the authors independently to improve the clarity and quality of the questions or corresponding answer options.The modifications made are mentioned in the text and highlighted in purple.

If you have any comments or suggestions, please add them directly in the relevant section of the Word document you received by email.

Condition of flight and body feathers

What is the condition of your parrot's plumage?

Choose one of the following answers

- The plumage is intact, there are no obvious signs of feather damage or plucking
- The plumage is mildly damaged or plucked; coverts and/or down feathers are missing in focal areas, but most of the feathers are still intact (though some of these may show signs of damage)
- The plumage is moderately damaged or plucked; coverts and/or down feathers are missing in several areas of the body leaving a patchy distribution or coverts are missing but down is still mostly or completely present
- The plumage is severely damaged or plucked; my parrot is (almost) completely devoid of its feathers

Number and appearance of droppings

Which statement best describes the droppings of your parrot?

Choose one of the following answers

- My parrot's droppings are well-formed and of normal colour, with a chalky white urate portion and minimal odour. The number of droppings are within my parrot's normal elimination pattern.
- My parrot's droppings are mostly within the normal range, though some occasional or slight changes in colour, odour, consistency or number can be observed.
- My parrot's droppings are excessive in number, and/or are discoloured, excessively watery and/or contain undigested food particles.
- My parrot's droppings are scant in number and/or volume, and contain fresh blood or have a tarry black colour.
- I don't check droppings.

Pectoral muscle condition score

Please select one the following score according to the muscle condition of your parrot.

HOW TO CHECK YOUR BIRDS SHAPE

• Getting hands on is key. Not all birds are used to being handled but it is difficult to judge if your bird is the right weight by sight. You will need to gently feel your bird, using restraint if necessary.

• Use bare hands and not gloves to handle birds as then you can judge the tightness of grip. If you need to protect yourself use a cloth or towel.

• Small birds can be held in one hand with the neck between the first and second finger and the bird's back against the palm so that the wings and body are gently restrained in the closed hand.

• Larger parrots may take two people, one to hold the bird and the other to assess its body condition. A towel or cloth is used over the open hand to grasp the bird firmly behind its head and neck. The towel is then wrapped around the wings and body to prevent flapping. Gently stroking the top of the head and talking to the bird gently will help to calm it.

• Gently run your fingertips down the centre of the front of the bird in the midline over the breast area. You should be able to feel a bony ridge (known as the keel or breast bone). This should be easy to feel but not too prominent.

• Next, run your fingers at right angles to the keel across the breast muscles. If these feel shrunken so that the keel sticks out prominently your bird is too thin. If the breast muscles are just rounded but you can still feel the keel your bird is in good condition. If you cannot feel the keel and the muscles are very rounded or you can feel or see fat moving underneath the skin your bird is overweight.

• The breast muscle can also vary in size depending on how much exercise your bird gets so if it flies a lot it will have larger firmer breast muscles than a bird who does not fly. However, the same criteria still apply in assessing body condition prominence of the bony keel and presence of fat underneath the skin.

For more information about the score please consult this link: https://www.ukpetfood.org/spotlight-on-obesity/how-to-identify-if-a-pet-needs-help/bird-size-o-meter-new.html

Choose one of the following answers

- I can't calculate the score because my parrot doesn't accept handling, and I don't feel comfortable insisting, it's too stressful and I'm afraid of hurting or scaring them.
- **1**

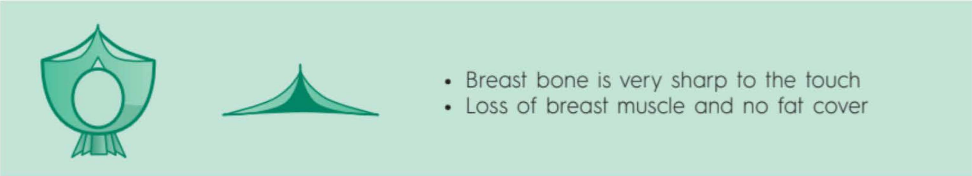

- **2**

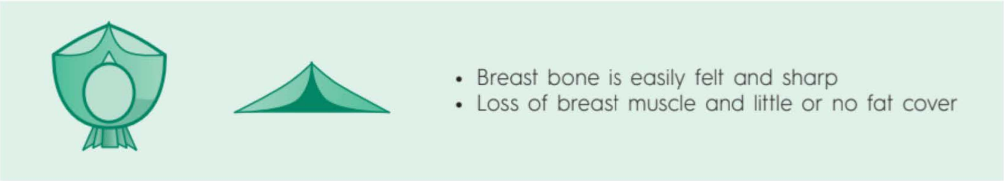

3

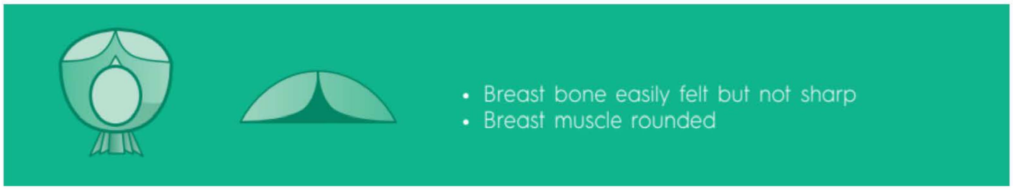

4

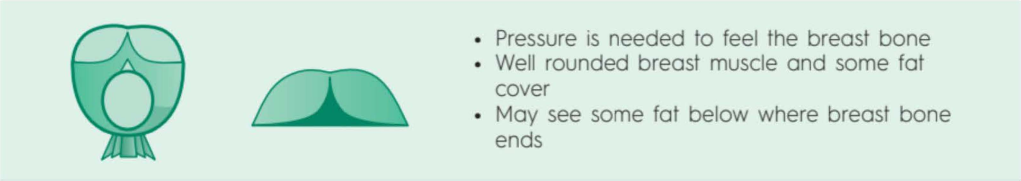

5

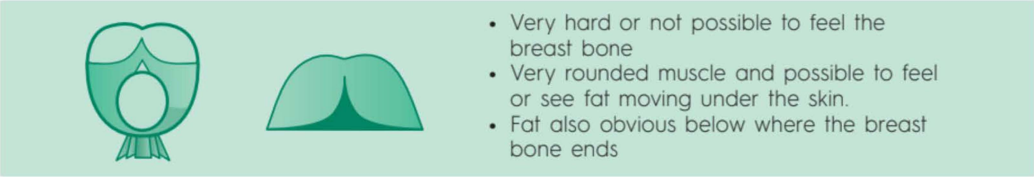

Presence of diagnosed diseases

Has your parrot been diagnosed with any disease by a veterinarian?

If yes, please indicate the disease(s) and any medication they are currently taking in the comment box

Choose one of the following answers

- No
- Yes

Please enter your comment here:

Signs of illness

(**Auhtors' note:** We have added new signs of illness, modified existing ones, and combined others)

Do you notice any of these conditions?

	Yes	No	I am not sure
Redness, swelling or loss of feathers around eyes			
Discharge from eyes, nares (nostrils), or mouth			
Crusty material in or around nares or flakiness on the skin or beak			
Overgrown beak and/or nails			

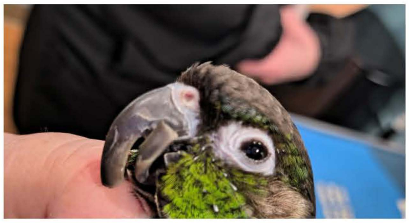

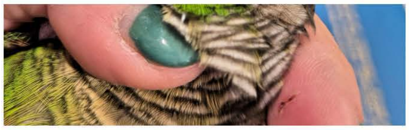			
Upper and lower beak not properly aligned<br>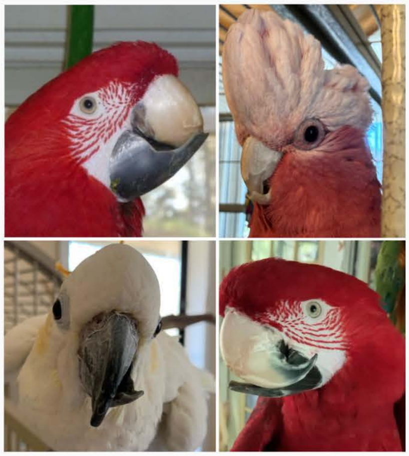			
Vomiting or regurgitation			
Signs of labored breathing, such as tail bobbing, open-mouth breathing, or abnormal respiratory sounds			
**Feather abnormalities**<br>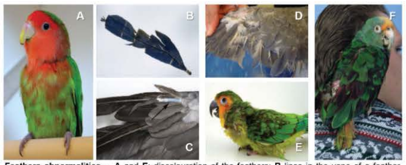<br><br>**Feathers abnormalities** - **A** and **E**: discolouration of the feathers; **B** lines in the vane of a feather generally oriented perpendicular to the shaft; **C**: pin or blood feather (black arrow) **D**: abraded feathers; **F**: feather discolouration and depigmentation, overall dull- and ragged looking plumage. From: Plumage disorders in psittacine birds - part 1: feather abnormalities. van Zeeland, Y.R.A.; Schoemaker, N.J. (2014).			
Signs of self-mutilation			
Blood loss or injury			
Fluffed posture, droopy wings, and sleeping more than usual			
Enlargement or swelling on the body			
>10% increase or decrease of body weight within a week			
Inability to perch or sitting at the bottom of the cage			
Repeated straining, often with tail bobbing and fluffed posture			
Cloaca protrudes outside the body (prolapse)			
Lameness or shifting of body weight			
Uncontrolled or irregular movement of the head, wings, or feet			
Swelling, ulcers, or lesions under the feet			

Vet diagnosis (follow up question for Signs of illness)

Were the condition(s) listed above diagnosed by a veterinarian?

Choose one of the following answers

- Yes, all conditions
- Only some conditions
- No

Treatments for (follow up questions for Signs of Illness)

Is your parrot currently receiving medication prescribed by a veterinarian for any of the conditions listed above?

Choose one of the following answers

- Yes, my parrot is receiving treatments prescribed by the veterinarian
- No, but my parrot is receiving over-the-counter medications
- No, my parrot is not receiving any medication

Section 3: Housing and physical activity

Please note the following:

• **Indicators in green** were thoroughly discussed during the meetings, and their inclusion and phrasing as questions were agreed upon. No further attention is required.
• **Indicators in orange** were discussed during our last meeting and have since been modified based on that discussion.

If you have any comments or suggestions, please add them directly in the relevant section of the Word document you received by email.

Enclosure type

Where is your parrot's enclosure located?

If your parrot has multiple enclosures, please specify all locations

Enclosure: space where the parrot sleeps, eats, drinks, and stays when it can't be supervised

	Main enclosure (space where the parrot stays most of time)	Secondary enclosure (additional space used occasionally)	Not applicable
Living room			
Hallway			
Kitchen			
Bedroom			
Garage			
Basement			
Parrot lives indoor and does not have a dedicated enclosure			
Room exclusively dedicated to the parrot			
Outdoor enclosure			
Other			

Enclosure size

Which statement most accurately describes the size of your parrot's main enclosure (where it resides most of the daytime) and its ability to move around?

For information on species wingspan measurements and recommended minimum cage sizes, please consult the following link: https://naturalinspirationsparrotcages.com/p/wingspan-info

Choose one of the following answers

- My parrot has ample space to move, climb, hop and fly
- My parrot has at least 2 wingspan of space in all directions to move, climb, and hop
- My parrot is unable to fully extend its wings in one or more directions, with limited space to move

Enclosure material

What type of material is your parrot's enclosure made from?

Check all that apply

- [ ] Stainless steel
- [ ] Powder-coated metal
- [ ] Wrought iron
- [ ] Plastic-coated wire, zinc, wood or acrylic
- [ ] I don't know
- [ ] Other:

Bars orientation

Please select the statement(s) that most accurately describe the bar orientation of your parrot's enclosure(s).

Check all that apply

- [ ] The enclosure(s) has grid-patterned bars
- [ ] The enclosure(s) has vertical bars
- [ ] The enclosure(s) has horizontal bars
- [ ] There are no bars to allow climbing (e.g. solid panels made of glass, acrylic)

Number of perches

How many perches are available in the parrot's enclosure(s)?

Choose one of the following answers

- ( ) > 5
- ( ) 3 – 5
- ( ) 1 – 2
- ( ) There are no perches

Perches' material

Have you checked whether the material of the perches provided is safe for parrots?

Choose one of the following answers

- ( ) Yes, I consulted my veterinarian or a behavioural consultant
- ( ) Yes, I researched myself online, in books, or magazines
- ( ) No

Perches' diameter

Do you offer differences in...

	Yes	No
perches' sizes	( )	( )
perches' material	( )	( )

Provision of opportunities that allow movement and climbing

Please indicate if your parrot receives the following types of enrichment that promote movement and climbing.

	Inside the enclosure			Outside the enclosure	
	Yes	No		Yes	No
Ropes	○	○		○	○
Swings	○	○		○	○
Ladders	○	○		○	○
Boings	○	○		○	○

Opportunity to spent time outside the enclosure

How often does your parrot spend time out of its enclosure ?

Choose one of the following answers

- Most or all of its time (e.g. only in the enclosure when sleeping or when there is no human supervision)
- Every day, for 3 hours or more
- Every day, for 3 hours or less
- Several times a week, but not every day
- Never or less than once a week on average

Presence of a retreating area/room to rest, sleep or withdraw

Does your parrot have unlimited access to an undisturbed area where it can rest, sleep or retreat from potential stressful situations (noise, presence of unfamiliar people, other animals etc.)?

Choose one of the following answers

- Yes
- No

Hygiene: frequency of cleaning food and water bowls, foraging toys, enclosure

How often do you clean the...

	Everyday or after every use	Several times a week, but not every day	Once a week or less
Enclosure (cage, room, aviary)	○	○	○
Food bowl	○	○	○
Water bowl	○	○	○
Foraging toys	○	○	○

Climate (temperature and humidity)

Do you regularly check the humidity and temperature to ensure they are appropriate for your parrot's species?

Choose one of the following answers

- Yes, both
- Only temperature
- Only humidity
- No

Air quality

How often do you refresh the air in the area where your parrot lives and spends most of its time?

Choose one of the following answers

- More than once per day / I use an air purifier / My parrot lives outdoor
- Once per day / I use an air purifier but is not constantly activated
- Several times a week, but not every day
- Once per week or less

Access to outdoor spaces

How often does your parrot spend time outdoor, if environmental circumstances permit (no risk of predation or mosquitoes' bites, appropriate temperature)?

Choose one of the following answers

- At least once a day
- At least once a week
- Less than once a week on average
- Never

Exposure to direct sunlight/UV light

How often do you expose your parrot to a UVA and UVB lamp or direct sunlight without a window in between?

Choose one of the following answers

- Everyday
- Several times a week, but not every day
- Once per week or less
- Never

Opportunity to spend time in high positions

Does your parrot have the opportunity to perch in high locations that are out of reach of people and other animals?

Choose one of the following answers

- Yes, both inside and outside of the enclosure
- Only inside the enclosure
- Only outside the enclosure
- No

Ability to fly

How would you describe your parrot's flight ability?

If you are unsure of your parrot's flying ability, please select the answer "I don't know." Avoid attempting to assess it, as it could lead to injury or stress for your parrot.

Choose one of the following answers

- My parrot flies in all directions and maintains height during flight
- My parrot flies in a downward trend and horizontally but cannot gain altitude
- My parrot attempts flying but is unable to do so and falls to the ground
- My parrot does not fly or does not attempt to fly
- I don't know

Wing trim (pop up question for ability to fly) - Answer options will be accompanied by illustrations

Has your parrot received a wing trim?

Please note that wing trimming is illegal in some countries

Choose one of the following answers

- No
- My parrot has received a bilateral skinny wing trim
- My parrot has received a bilateral, transverse wing trim
- My parrot has received a bilateral wing trim which includes the primaries and secondaries
- My parrot has received a unilateral wing trim
- My parrot has been permanently deflighted through a surgical procedure (e.g., pinioning)

Opportunity to fly

How often does your parrot have the opportunity to fly in a safe space?

Choose one of the following answers

- Every day for 4 or more hours
- Every day for less than 4 hours
- Several times a week, but not every day
- Never or less than once a week on average

Level of activity

How much time does your parrot spend each day moving, climbing, and flying?

Choose one of the following answers

- I don't know
- 1 hour or less
- Between 1 and 2 hours
- Between 2 and 4 hours
- 4 or more hours

Section 4: Provision of enrichment and exploration

Please note the following:

- **Indicators in green** were thoroughly discussed during the meetings, and their inclusion and phrasing as questions were agreed upon. No further attention is required.
- **Indicators in orange** were discussed during our last meeting and have since been modified based on that discussion.
- **Indicators in purple** were revised by the authors independently to improve the clarity and quality of the questions or corresponding answer options.The modifications made are mentioned in the text and highlighted in purple.

If you have any comments or suggestions, please add them directly in the relevant section of the Word document you received by email.

Provision of foraging and cognitive enrichment

How often do you provide the following types of enrichment?

	Every day	Weekly	Few times per month or less	Never
Certified chewable toys, cardboard or paper without ink, natural, not toxic and untreated cork and branches that can be safely chewed and destroyed				

Puzzles and problem solving games	○	○	○	○
Multiple food stations	○	○	○	○
Scatter feeding (e.g. foraging mat, spreading food out in various locations) and foraging tray or box (food mixed with inedible items)	○	○	○	○
Larger chunks of food or whole food items (with or without skewers)	○	○	○	○
Non destructible puzzle feeders/foraging toys	○	○	○	○
Destructible foraging toys	○	○	○	○
Interactive toys that make sounds and/or move	○	○	○	○
Auditory enrichment (e.g. radio, music)	○	○	○	○
Visual enrichment (e.g. tv or tablets)	○	○	○	○

Interaction with enrichment

How does your parrot interact with the enrichment provided?

Types of interaction: manipulation with beak and/or feet (e.g., chewing, grasping, exploring) or reactions to sound and visual cues.

	Frequently and repeatedly uses with the enrichment, exploring and manipulating it extensively, showing continued interest until the item is destroyed (in case of destructible items) or food/contents is removed (if applicable)	Shows brief interest in the enrichment and ignores it at other times	Does not use or interact with the enrichment at all, and completely ignores it	I don't know
Certified chewable toys, carboard or paper without ink, natural, not toxic and untreated cork and branches that can be safely chewed and destroyed	○	○	○	○
Puzzles and problem solving games	○	○	○	○
Multiple food stations	○	○	○	○
Scatter feeding (e.g. foraging mat, spreading food out in various locations) and foraging tray or box (food mixed with inedible items)	○	○	○	○
Larger chunks of food or whole food items (with or without skewers)	○	○	○	○
Non destructible puzzle feeders/foraging toys	○	○	○	○
	**Frequently and repeatedly uses with the enrichment, exploring and manipulating it extensively, showing continued interest until the item is destroyed (in case of destructible items) or food/contents is removed (if applicable)**	**Shows brief interest in the enrichment and ignores it at other times**	**Does not use or interact with the enrichment at all, and completely ignores it**	**I don't know**
Destructible foraging toys	○	○	○	○
Interactive toys that make sounds and/or move	○	○	○	○
Auditory enrichment (e.g. radio, music)	○	○	○	○
Visual enrichment (e.g. tv or tablets)	○	○	○	○

Provision of foraging enrichment in relation to the daily food ration

How is foraging enrichment provided for your parrot?

Foraging enrichment includes toys, devices, or other opportunities that encourage natural behaviors such as searching, procuring, and extracting food.

❶ Choose one of the following answers

○ For treats and more than half of the daily food ration

- For treats and about half of the daily food ration
- For treats and a small portion of of the daily food ration
- Only for treats

Time spent foraging

(**Authors' note:** we revised the answer options to make them more reliable and easier for owners to select)

How much time does your parrot spend daily foraging (i.e., searching for, procuring, and extracting food from toys that you provide)?

Choose one of the following answers

- 4 hours or more
- Between 4 and ~~3~~ 2 hours
- Between ~~4~~ 2 and ~~3~~1 hours
- ~~Less than~~ Up to 1 hour
- My parrot does not forage
- I don't know

Opportunities to select items based on preference (e.g., for colour, shape or type of material)

Do you select toys based on your parrot's preference for shape, size and colours?

Choose one of the following answers

- Yes
- No

Enrichment replacement

How often do you replace toys/chewable items/ climbing enrichment by introducing new different one/s?

Choose one of the following answers

- Every day
- Weekly
- Few times per month or less
- Never

Response to novel objects

(**Authors' note:** we removed " after initially being reluctant to do so" to simplify the answer options)

How does your parrot generally react towards unfamiliar objects (e.g. new toys, new home decoration) in its proximity?

Choose one of the following answers

- My parrot actively approaches the object touching, chewing and manipulating it; certain objects may still trigger freezing or withdrawing responses.
- My parrot appears interested in the object, and cautiously approach it~~, after initially being reluctant to do so~~; certain objects (e.g. larger furniture or electronic household devices) may still trigger freezing or withdrawing responses.
- My parrot withdraws or freezes to most objects; certain objects (e.g. larger toys, larger furniture or electronic household devices) may trigger more freezing or withdrawing responses.
- My parrot shows one or more of these behaviours: excessive or incessant screaming, frantic movements, flying away, falling of the perch. This response happens with any type of unfamiliar object.

Alertness

What is your parrot's current alertness level?

Choose one of the following answers

- My parrot is very active and curious. It eagerly explores new objects, interacts frequently with people and toys, and responds quickly to its surroundings
- My parrot responds to sounds, movements, and interactions. It engages with toys, interacts with people, and explores its environment with moderate enthusiasm
- My parrot reacts occasionally but needs encouragement to engage. It shows some interest in its surroundings and sometimes participates in activities
- My parrot is mostly inactive or lethargic, shows little interest in its surroundings and does not respond much to things happening around it

Section 5: Nutrition and maintenance behaviours

Please note the following:

- **Indicators in green** were thoroughly discussed during the meetings, and their inclusion and phrasing as questions were agreed upon. No further attention is required.
- **Indicators in orange** were discussed during our last meeting and have since been modified based on that discussion.

If you have any comments or suggestions, please add them directly in the relevant section of the Word document you received by email.

Composition of the diet

Which of the following foods do you provide?

	Main component of the diet	Moderate to small quantities	As treat, training reward or vehicle for medications	Not provided
Pellet and other formulated food	○	○	○	○
Fresh vegetables	○	○	○	○
Fresh fruit	○	○	○	○
Sprouted seeds	○	○	○	○
Grains	○	○	○	○
Legumes	○	○	○	○
Tofu and other plant-based proteins	○	○	○	○
Seed mix	○	○	○	○
Nuts	○	○	○	○
Eggs	○	○	○	○
Dairy products	○	○	○	○
Other animal-based proteins	○	○	○	○
Nectar	○	○	○	○
Processed food specifically designed for human consumption	○	○	○	○

Food Selectivity

Does your parrot eat all types of food that you provide?
Observing the animal while it eats is preferable, but checking the food bowl can also provide valuable insights.

Choose one of the following answers

- Yes
- No
- I don't know

Diet Apropriateness

Have you checked whether the diet you provide is balanced and appropriate for your parrot's species?

Choose one of the following answers

- Yes, I consulted my veterinarian or a behavioural consultant
- Yes, I researched by myself online, in books, or magazines
- No

Availability of fresh and clean water

How often do you change your parrot's water?

Choose one of the following answers

- More than twice a day or as needed throughout the day
- Twice a day
- Once a day
- Less than once per day

Changes in food and/or water consumption

Have you observed a change in appetite or water intake by your parrot in the last 2 weeks?
i.e. you observed these changes directly observing the parrot's behaviour or by checking the amount of food or water consumed.

Choose one of the following answers

- No
- Yes
- I don't know

Daytime spent sleeping / resting

What daytime does your parrot sleep / rest?

Check all that apply

- Morning
- Afternoon
- Evening
- Night
- I don't know

Changes of resting-sleeping patterns

Have you noticed any changes in the sleeping pattern of your parrot in the last two weeks?
e.g. sleeping more than usual or at times of the day when it typically does not sleep.

Choose one of the following answers

- No
- Yes
- I don't know

Opportunity to bathe

Please indicate how often you provide the following bathing opportunities for your parrot

	Every day	Weekly	Few times per month or less	Never
Mist	○	○	○	○
Shower	○	○	○	○
Shallow dish or tray	○	○	○	○
Other	○	○	○	○

Interest in bathing

How does your parrot bathe when you provide the opportunity to do so?

Choose one of the following answers

- My parrot always bathes on its own
- My parrot bathes on its own but sometimes needs encouragement to do so
- My parrot always needs encouragement to bathe
- My parrot doesn't bathe, and I don't try to change that
- My parrot tries to avoid bathing, but I make sure it gets bathed anyway
- I don't know

Beak maintenance

Does your parrot engage in self-maintenance behaviors for its beak?

e.g. rubbing it against hard objects, grinding it by making a rhythmic motion with the upper and lower mandibles, or using bars or other objects to clean the inside of its beak

Choose one of the following answers

- Yes
- No
- I don't know

Changes in preening activity

Have you noticed any changes in your parrot's self-preening behavior (such as using its beak to clean, arrange, or maintain its feathers) over the past month?

Choose one of the following answers

- No
- Yes
- I don't know

Section 6: Social and reproductive behaviours

Please note the following:

• **Indicators in green** were thoroughly discussed during the meetings, and their inclusion and phrasing as questions were agreed upon. No further attention is required.
• **Indicators in red** received comments from our previous round of surveys but have not yet been discussed. You can find the comments, our answers to them, and potential solutions in the text.

If you have any comments or suggestions, please add them directly in the relevant section of the Word document you received by email.

Social housing (alone vs pair vs group)

Please select the option that best describes your parrot's social contact with other parrot.

Choose one of the following answers

- My parrot is housed together with two or more other parrots and can continuously interact with them
- My parrot is housed together with one parrot and they can continuously interact
- My parrot can physically interact with one or more parrots at certain times of the day (or week), but is not continuously housed together with other parrots
- My parrot is housed individually, but is able to see other parrots and can vocally interact with them
- My parrot lives alone and does not have any type of contact with other parrots

Social behaviours

(**Authors' note:** to simplify the table, the number of answer options was reduced from five (On every encounter, most encounter, some encounters, rarely, never) to three)

(**Comment 1:** Add "Regurgitates food towards a person in the household" > **Response:** this answer option is already included in the table "behaviours directed towards humans")

(**Comment 2:** Add "my parrots are housed together and copulate" > **Response:** this answer option is already included in the table as "Mating with another parrot")

How frequently do these social interactions occur?

	On all or most encounters	On some encounters	Rarely to never
Staying right beside another parrot			
Feeding alongside another parrot			
Preening another parrot			
Being preened by another parrot			
Vocal interaction with another parrot (contact calls)			
Mating with another parrot			
Stalking (i.e. following or watching insistently) another parrot			
Stalked (i.e. followed or watched insistently) by another parrot			
Regurgitating food for other parrot			
Having food regurgitated by another parrot			
Attacks (biting, lunging) another parrot			
Being attacked (bitten or lunged at) by another parrot			

Reproductive behaviours

(**Comment:** Add "Regurgitates food on a special toy or item in the cage" > **Response:** added to the table)

How frequently do you observe these behaviors?

	Every day	Weekly	Few times per month or less	Never
Rubbing its cloaca (vent area) against objects such as perches, cage bars, toys				
Actively seeking or spending time in dark, enclosed, or secluded spaces such us underneath furniture, inside drawers or cabinets, behind cushions, or in boxes or clothing.				
Lunging, biting, vocalizing aggressively, or chasing anyone who approaches or gets too close to a specific area (e.g. cage or nest)				
Egg laying				
Regurgitating food and directing it towards its own body parts (e.g. its foot)				
Regurgitating food on a special toy or item in the cage				

Section 7: Parrot-Human interactions

Section 7: Parrot-Human Interactions

Please note the following:

- **Indicators in green** were thoroughly discussed during the meetings, and their inclusion and phrasing as questions were agreed upon. No further attention is required.
- **Indicators in orange** were discussed during our last meeting and have since been modified based on that discussion.
- **Indicators in purple** were revised by the authors independently to improve the clarity and quality of the questions or corresponding answer options.The modifications made are mentioned in the text and highlighted in purple.
- **Indicators in red** received comments from our previous round of surveys but have not yet been discussed. You can find the comments, our answers to them, and potential solutions in the text.

If you have any comments or suggestions, please add them directly in the relevant section of the Word document you received by email.

Time spent in presence of humans

How many hours per day is your parrot surrounded by you and/or familiar people (e.g. partner, family member)?

Choose one of the following answers

- ○ It varies greatly per day
- ○ <1h
- ○ 2h – 4h
- ○ 4h - 8h
- ○ > 8h
- ○ All time, including night

Types of interaction with humans (table 1)

How often do you allow your parrot to engage in the following behaviors?

	Every day	Weekly	Few times per month or less	Never
Sitting on shoulder, lap, or another part of the body	○	○	○	○
Gently nibbling or grooming hair, beard, eyelashes, or skin with its beak (preening)	○	○	○	○
Crawling under clothes or blankets	○	○	○	○

Types of interaction with humans (table 2)

How often do the following interactions take place with the parrot?

	Every day	Weekly	Few times per month or less	Never
Petting the parrot's head, cheeks and/or neck	○	○	○	○
Petting the parrot under the wings	○	○	○	○
Petting the parrot's chest	○	○	○	○
Petting the parrot's back and tail	○	○	○	○
Holding and shaking the parrot's beak while playing	○	○	○	○
Gently responding to the parrot's vocalizations	○	○	○	○
Talking to the parrot	○	○	○	○
Yelling at the parrot	○	○	○	○
	Every day	Weekly	Few times per month or less	Never
Playing music for my parrot	○	○	○	○
Taking the parrot along when going out	○	○	○	○
Training the parrot	○	○	○	○
Kissing the parrot	○	○	○	○
Providing food by mouth or allowing the parrot to eat from	○	○	○	○

[illegible] the mouth				
Pressing on parrot's chest to encourage it to step up onto hands, arms, or an offered perch				
Holding the parrot's body in the hands (with or without gloves or a towel)				
Grabbing the parrot with a net or a towel				

Parrot response to training (Pop-up question if the owner indicates they train their parrot)

(**Comment:** Maybe change "commands" to "instructions" >**Solution:** remove "to commands" from the first answer option)

How does your parrot respond during training session?

Choose one of the following answers

- Very focused and responds quickly ~~to commands~~
- Mostly focused, but gets distracted sometimes
- Responds sometimes, but often gets distracted
- Rarely focused, responds only occasionally
- Does not respond or shows no interest in training

Behaviours directed towards humans

(**Authors' note 1:** we revised the question and added a pop-up prompt to ask how many people the parrot displays the behavior described in the table)

(**Authors' note 2:** we added "Moving toward a person and biting" to the table)

How frequently does your parrot display the behavior below towards humans ~~you or other household members~~?

	Every day	Weekly	Few times per month or less	Never
Offering the head/neck to be petted				
Contact calls/vocalizations				
Crouching with the head down				
Bowing and bobbing the body				
Voluntarily steps up onto your hand, arm, or an offered perch without encouragement				
Begging for food (raise its wings, flutter them and bob its head up, and down in a rhythmic pattern)				
Regurgitating food				
Masturbation (rubbing the cloaca against humans)				
Moving toward a person and biting				

Human selectivity (pop-up question for behaviours directed towards humans)

To how many people does your parrot display each of the behaviour(s) selected above?

	To everyone	To few people	Only to me
Offering the head/neck to be petted			
Contact calls/vocalizations			
Crouching with the head down			
Bowing and bobbing the body			
Voluntarily steps up onto your hand, arm, or an offered perch without encouragement			
Begging for food (raise its wings, flutter them and bob its head up, and down in a rhythmic pattern)			
Regurgitating food			
Masturbation (rubbing the cloaca against humans)			
Moving toward a person and biting			

Response upon contact with caregiver

(**Authors' note 1:** to simplify the table, the number of answer options was reduced from six (Every time, most of the times, sometimes, rarely, never) to four)

(**Authors' note 2:**we modified the answer option "My parrot remains seated when I am approaching it" to indicate a fearful freezing response)

How does your parrot respond to you as the caregiver?

	Every or most times	Sometimes	Rarely to never	I don't know
My parrot accepts my presence near the enclosure or stand				
My parrot accepts physical contact initiated by me				
My parrot initiates physical contact itself by actively approaching me				
My parrot ~~remains seated~~ stays completely still and does not react when I am approaching it				
My parrot tries to lunge, attempt to bite or chase me when I am approaching it				
My parrot tries to escape and avoid contact when I am approaching it				

Response upon contact with familiar person

(**Authors' note 1:** to simplify the table, the number of answer options was reduced from six (Every time, most of the times, sometimes, rarely, never) to four)

(**Authors' note 2:** we modified the answer option "My parrot remains seated when I am approaching it" to indicate a fearful freezing response)

How does your parrot respond to a familiar person (e.g. partner, family member, friend)?

	Every or most times	Sometimes	Rarely to never	I don't know
My parrot accepts their presence near the enclosure or stand				
My parrot accepts physical contact initiated by them				
My parrot initiates physical contact itself by actively approaching them				
My parrot ~~remains seated~~ stays completely still and does not react when they are approaching it				
My parrot tries to lunge, attempt to bite or chase them when they are approaching it				
My parrot tries to escape and avoid contact when they are approaching it				

Comfort behaviour around humans

(**Comment 1:** Define comfort behavior > **Solution:** delete "feel comfortable to" from the question)

(**Comment 2:** Delete "in the presence of all household members" and reword third answer for "in the presence of other familiar persons (e.g. partner, family members, friend)" > **Solution:** we decided to keep "household member" as it better captures situations where parrot owners live with flatmates who are not family members. Additionally, we replaced "familiar person" and "stranger"" with "people who come by regularly" and "people who come by infrequently", respectively, to provide a more objective and context-neutral description)

Does your parrot ~~feel comfortable to~~...

	eat, drink, rest and clean its feathers...				spend time in locations where it can be easily reached by a human...		
	Yes	No	I don't know		Yes	No	I don't know
in your presence?							
in the presence of all household members?							
in presence of ~~a familiar person (e.g. partner, family members, friend)~~ people that come by regurarly?							
in presence of ~~in the presence of a stranger (e.g., a repair technician, delivery person)~~ people that come by infrequently?							

Section 8: Abnormal and fear-related behaviours

Please note the following:

• **Indicators in green** were thoroughly discussed during the meetings, and their inclusion and phrasing as questions were agreed upon. No further attention is required.
• **Indicators in purple** were revised by the authors independently to improve the clarity and quality of the questions or corresponding answer options.The modifications made are mentioned in the text and highlighted in purple.
• **Indicators in red** received comments from our previous round of surveys but have not yet been discussed. You can find the comments, our answers to them, and potential solutions in the text.

If you have any comments or suggestions, please add them directly in the relevant section of the Word document you received by email.

Disruptive vocalization or screams

(**Authors' note:** we removed "but never incessantly" and modify "which can still be at several times during the day" with " throughout the day" to simplify the answer options)

How often does your parrot produce disruptive, loud vocalizations or screams?

The answer options range from the worst conditions at the top to the best conditions at the bottom

Choose one of the following answers

- Never
- Rarely~~, but never incessantly,~~ and mostly limited to specific moments of the day (e.g. morning and evening) or in response to specific and uncommon situations (e.g. sudden loud noise)
- Occasionally ~~which can still be at several times during~~ throughout the day~~, but never incessantly~~ and mostly when exposed to specific contexts (e.g. when left alone, presence of unfamiliar people/guests)
- Frequently, and incessant screaming can occur but mostly in specific contexts (e.g. when left alone, presence of unfamiliar people/guests)
- For the majority of the day, and sometimes incessantly for hours with no apparent reason or cause

Expression of avoidance and escape behaviours

When does your parrot display any of the following behaviors?

The answer options range from the worst conditions at the top to the best conditions at the bottom

	Tremors or shivering, freezing, hiding, withdrawing	Attempting to escape by flying or moving away, possibly falling off the perch, screeching/high-pitched screams
I don't know		
Never		
Mostly in response to specific or uncommon situations (e.g. visit to the vet, sudden loud noise)		
Mostly when exposed to a certain situation, outside of its daily environment (e.g. outdoor activity, visit of a new place)		
Predominantly when exposed to changes in its daily environment (e.g. presence or approach by new, unfamiliar people/guests or animals, change of furniture, new house decorations, provision of new toys)		
Most of the time, even in its daily environment		

Abnormal, sham and excessive behaviours

(**Comment:** Sham flying: I still think this is normal behavior for birds that cannot fly and wish to be picked up and transported. It's not a negative thing, it's a positive way to communicate instead of screaming! > **Solution:** we decided to remove sham flying from the current version, as the description alone could be easily misinterpreted by owners. It will only be included if it can be accompanied by a video clearly illustrating the behaviour)

Does your parrot exhibit any of the following behaviors?

The answer options range from the best condition on the left (never) to the worst condition on the right (always).

	Yes	No	I am not sure
Pacing: repetitive walking back and forth along a fixed path			
Route tracing: repeatedly follow the same path or pattern within its enclosure, such as moving along a specific perch, climbing the same section of the cage, or flying in a predictable loop			
Swaying and rocking: repeatedly shift its body side to side			

or back and forth in a rhythmic motion			
Tongue rolling and flicking: repetitive quick movement of the tongue or rolling or flicking of the tongue inside or outside the beak	◯	◯	◯
Beak clacking: rapid, repetitive, excessive or compulsive clicking or clacking of the beak	◯	◯	◯
Beak rubbing: repeatedly rubbing the beak against surfaces such as perches or bars	◯	◯	◯
Repetitive licking: repeated licking of surfaces such as cage bars, perches, or walls	◯	◯	◯
	**Yes**	**No**	**I am not sure**
Spot pecking: repetitively peck at a specific spot, such as a cage bar, perch, wall, or even an imaginary point	◯	◯	◯
Sham bathing: mimic bathing motions without the presence of water or an appropriate bathing substrate	◯	◯	◯
~~Sham flying: repetitively twitches its wings without taking flight (distinct from learned wing flapping)~~	◯	◯	◯
Sham chewing and chewing not chewable items: mimics chewing motions or engages with non-chewable objects (e.g., metal bars or plastic) or surfaces	◯	◯	◯
Toe-nail biting: repeatedly bite or nibble at their own toes or nails	◯	◯	◯
Feeding objects (e.g. mirrors, toys)	◯	◯	◯

Submit

Datenschutzerklärung

Prototype Parrot Welfare Assessment Tool: second refinement

Dear Expert, thank for participating in our project!

In this document, you can provide your comments and suggestions regarding the indicators included in the prototype of the welfare assessment tool, which you received by email in PDF format. The prototype includes:

- **Green indicators**: they were thoroughly discussed during the meetings, and their inclusion and phrasing as questions were agreed upon. No further attention is required.
- **Orange indicators**: they were discussed during our last meeting and have since been modified based on that discussion.
- **Purple indicators**: they were revised by the authors independently to improve the clarity and quality of the questions or corresponding answer options. The modifications and the explanations for them (authors' notes) are provided in the question box of each indicator in the prototype document.
- **Red indicators**: they received comments from our previous round of surveys but have not yet been discussed. You can find your comments, our responses, or potential solutions in the question box of each indicator within the prototype document.

This document is divided into 8 sections (page 1-8), each corresponding to a section of the prototype. Please review the PDF file of the prototype, and when you have a comment, provide it in this document. Please write your comment in the corresponding section of this document where that specific indicator is located (section 1, section 2, etc.), based on its colour (green, orange, purple, red). Once you have provided your comments, please send the document back to me by email (andrea.piseddu@vetmeduni.ac.at).

Please note that:

- Participation is voluntary and anonymous.
- You can withdraw at any moment of the study.
- You have to be at least 18 years old to participate.
- The results of this survey will be included in a PhD thesis and published in a scientific journal.
- Your name will not appear in any published documents related to this study, and your statements will remain anonymous.
- Your personal data will be treated in compliance with the European General Data Protection Regulation and will be stored only as long as needed for the purpose of this study.
- The prototype we sent you via email is a confidential document and should not be shared with anyone.

Before starting to fill out this document please tick the box below:

- **I confirm to have read and understood the above statements and I give my informed consent.** ☐

If you have any questions or concerns, please do not hesitate to contact the responsible person of this study:

Andrea Piseddu
Institute of Animal Welfare Science, University of Veterinary Medicine Vienna, Vienna, Veterinaerplatz 1, 1210 Vienna, Austria
Andrea.Piseddu@vetmeduni.ac.at

Section 1: General information

Green indicators (modified based on previous comments or suggestions, which have not received any further feedback and are now finalized)

Do you have any comments on the indicators in green in this section? If so, please provide the name of the indicator along with your corresponding comment (Indicator name: comment).

Comments:

-

Section 2: Physical health

Green indicators (modified based on previous comments or suggestions, which have not received any further feedback and are now finalized)

Do you have any comments on the indicators in green in this section? If so, please provide the name of the indicator along with your corresponding comment (Indicator name: comment).

Comments:

-

Orange Indicators (modified based on the discussions from our previous meeting)

Are you satisfied with the updated versions of the indicators in orange?

If yes, no action is required. If not, please provide the name(s) of the indicator(s) you are not satisfied with, along with your corresponding comment (Indicator name: comment).

Comments:

-

Purple indicators (modified by the authors to improve quality and content)

Are you satisfied with the improved versions of the indicators in purple?
If yes, no action is required. If not, please provide the name(s) of the indicator(s) you are not satisfied with, along with your corresponding comment (Indicator name: comment).

Comments:

-

Section 3: Housing and physical activity

Green indicators (modified based on previous comments or suggestions, which have not received any further feedback and are now finalized)

Do you have any comments on the indicators in green in this section? If so, please provide the name of the indicator along with your corresponding comment (Indicator name: comment).

Comments:

-

Orange Indicators (modified based on the discussions from our previous meeting)

Are you satisfied with the updated versions of the indicators in orange?

If yes, no action is required. If not, please provide the name(s) of the indicator(s) you are not satisfied with, along with your corresponding comment (Indicator name: comment).

Comments:

-

Section 4: Provision of enrichment and exploration

Green indicators (modified based on previous comments or suggestions, which have not received any further feedback and are now finalized)

Do you have any comments on the indicators in green in this section? If so, please provide the name of the indicator along with your corresponding comment (Indicator name: comment).

Comments:

-

Orange Indicators (modified based on the discussions from our previous meeting)

Are you satisfied with the updated versions of the indicators in orange?

If yes, no action is required. If not, please provide the name(s) of the indicator(s) you are not satisfied with, along with your corresponding comment (Indicator name: comment).

Comments:

-

Purple indicators (modified by the authors to improve quality and content)

Are you satisfied with the improved versions of the indicators in purple?
If yes, no action is required. If not, please provide the name(s) of the indicator(s) you are not satisfied with, along with your corresponding comment (Indicator name: comment).

Comments:

-

Section 5: Nutrition and maintenance behaviours

Green indicators (modified based on previous comments or suggestions, which have not received any further feedback and are now finalized)

Do you have any comments on the indicators in green in this section? If so, please provide the name of the indicator along with your corresponding comment (Indicator name: comment).

Comments:

-

Orange Indicators (modified based on the discussions from our previous meeting)

Are you satisfied with the updated versions of the indicators in orange?

If yes, no action is required. If not, please provide the name(s) of the indicator(s) you are not satisfied with, along with your corresponding comment (Indicator name: comment).

Comments:

-

Section 6: Social and reproductive behaviours

Green indicators (modified based on previous comments or suggestions, which have not received any further feedback and are now finalized)

Do you have any comments on the indicators in green in this section? If so, please provide the name of the indicator along with your corresponding comment (Indicator name: comment).

Comments:

-

Red indicators (received comments from our previous round of surveys but comments have not yet been discussed)

Are you satisfied with how we have addressed the comments related to the indicators in red and the modifications made based on those comments?
If yes, no action is required. If not, please provide the name(s) of the indicator(s) you are not satisfied with, along with your corresponding comment (Indicator name: comment).

Comments:

-

Section 7: Parrot-human interactions

Green indicators (modified based on previous comments or suggestions, which have not received any further feedback and are now finalized)

Do you have any comments on the indicators in green in this section? If so, please provide the name of the indicator along with your corresponding comment (Indicator name: comment).

Comments:

-

Orange Indicators (modified based on the discussions from our previous meeting)

Are you satisfied with the updated versions of the indicators in orange?

If yes, no action is required. If not, please provide the name(s) of the indicator(s) you are not satisfied with, along with your corresponding comment (Indicator name: comment).

Comments:

-

Purple indicators (modified by the authors to improve quality and content)

Are you satisfied with the improved versions of the indicators in purple?
If yes, no action is required. If not, please provide the name(s) of the indicator(s) you are not satisfied with, along with your corresponding comment (Indicator name: comment).

Comments:

-

Red indicators (received comments from our previous round of surveys but comments have not yet been discussed)

Are you satisfied with how we have addressed the comments related to the indicators in red and the modifications made based on those comments?
If yes, no action is required. If not, please provide the name(s) of the indicator(s) you are not satisfied with, along with your corresponding comment (Indicator name: comment).

Comments:

-

Section 8: Abnormal and fear-related behaviours

Green indicators (modified based on previous comments or suggestions, which have not received any further feedback and are now finalized)

Do you have any comments on the indicators in green in this section? If so, please provide the name of the indicator along with your corresponding comment (Indicator name: comment).

Comments:

-

Purple indicators (modified by the authors to improve quality and content)

Are you satisfied with the improved versions of the indicators in purple?
If yes, no action is required. If not, please provide the name(s) of the indicator(s) you are not satisfied with, along with your corresponding comment (Indicator name: comment).

Comments:

-

Red indicators (received comments from our previous round of surveys but comments have not yet been discussed)

Are you satisfied with how we have addressed the comments related to the indicators in red and the modifications made based on those comments?
If yes, no action is required. If not, please provide the name(s) of the indicator(s) you are not satisfied with, along with your corresponding comment (Indicator name: comment).

Comments:

-

vetmeduni Veterinärmedizinische Universität Wien   vetmeduni Veterinärmedizinische Universität Wien

Load unfinished survey   Resume later

Parrot Welfare Assessment Tool: Owners Review

Information and Consent

vetmeduni University of Veterinary Medicine, Vienna   Utrecht University

Dear owner,
Thank you for taking part in this survey!
The aim of this survey is to test the prototype of a parrot welfare assessment tool for parrot owners, developed based on the results of 10 online focus group meetings with parrot experts.
The tool consists of several questions addressing different aspects of parrot welfare divided into eight sections.

Each question is accompanied by an emoji-based feedback system designed to help you interpret the results. In this version, the feedback appears alongside each question to support your evaluation. However, in the final version of the tool, feedback will be shown only at the end of the assessment, not during the answering process. At the beginning of each section, you will find a legend explaining how to interpret the emojis used in the feedback system.
Some questions are also accompanied by illustrative images to clarify their meaning. While some of these images are already included, others are currently missing. We are still in the process of identifying suitable visuals to add later.

Your task is to test the welfare assessment tool by answering the questions included in the eight sections. If you have more than one parrot, please complete the assessment with just one individual in mind. At the end of each section, you will be asked to provide feedback on the **clarity** and **practicality** of the questions. At the end of the assessment, there is a final section where you can provide an overall feedback on the tool. **Please complete this survey by 15.06.2025.**

You have the **opportunity to save partially finished surveys** by clicking on the "Resume later" button located at the top right of every page. After clicking this button, you will be asked to create a username and a password and to provide your email address. These credentials will allow you to return to your partially completed survey. Once you complete this step, you will receive a new link by email. **To resume the survey, please use only the new link** provided in that email and log in with the username and password you created.

Please note that:

- Participation is voluntary and anonymous.
- You can withdraw at any moment of the study.
- You have to be at least 18 years old to participate.
- The results of this survey will be included in a PhD thesis and published in a scientific journal.
- Your name will not appear in any published documents related to this study, and your statements will remain anonymous.
- Your personal data will be treated in compliance with the European General Data Protection Regulation and will be stored only as long as needed for the purpose of this study.

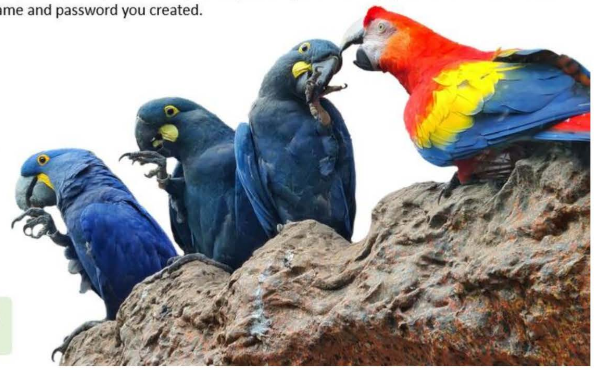

If you have any questions or concerns, please do not hesitate to contact the responsible person of this study:

Andrea Piseddu
Centre for animal Nutrition and Welfare, University of Veterinary Medicine Vienna, Vienna, Veterinaerplatz 1, 1210 Vienna
Andrea.Piseddu@vetmeduni.ac.at

By clicking the button "**Next**" and submitting your responses to the survey you are confirming to have read and understood the above statements and give your informed consent.

This survey is anonymous.

The record of your survey responses does not contain any identifying information about you, unless a specific survey question explicitly asked for it.

If you used an identifying access code to access this survey, please rest assured that this code will not be stored together with your responses. It is managed in a separate database and will only be updated to indicate whether you did (or did not) complete this survey. There is no way of matching identification access codes with survey responses.

Welcome to the Parrot Welfare Assessment Tool

This tool is designed to help caregivers evaluate the welfare of companion parrots (order *Psittaciformes*). It includes 77 questions divided into 8 sections, covering your parrot's physical condition, behaviour, and the care and management practices that may influence its welfare.

When answering the questions, base your responses on what has happened in the past month, unless a different time frame is specified. To answer some questions, it is important to be in front of your parrot, as direct observation may be necessary. The tool can be repeated monthly to help you monitor your parrot's welfare over time and detect any changes that may require attention.

Please note that this tool:

- is intended to provide a general overview of your parrot's welfare. It does not offer specific recommendations or diagnoses. Every parrot is unique, and interpreting welfare concerns can be complex.
- is not designed to assess the welfare of chicks or parrots kept primarily for breeding purposes.

If the results of the assessment highlight any welfare concerns or areas that could be improved, you may need to consider making some changes in your parrot's environment or daily care. Before taking action, it's important to consult with a qualified expert, such as a veterinarian experienced in avian care or a certified parrot behaviour consultant. Their support is essential to ensure that any changes you make are appropriate, effective, and tailored to your parrot's individual needs.

Thank you for taking the time to reflect on your parrot's well-being. Your commitment is an important step toward to promote a better quality of life for your feathered companion.

Section 1: General information

How to interpret your answers:

😍: Great - 🙂: Good - 😕: Room for improvement - 😟: Welfare concern - 😞: Serious welfare concern

Please consult a behavioural consultant or veterinarian to review your assessment results and, if needed, develop an effective plan to improve your parrot's welfare.

Name of your parrot

Species of your parrot

Sex

Choose one of the following answers

- Female
- Male
- Unknown

How old is your parrot?

How long have you been living with your parrot?

Rearing history

Please select the option that best describes your parrot's rearing history.

Choose one of the following answers

- Parent-reared and briefly socialized with humans for short amounts of time each week
- Parent-reared
- Initially parent-reared and later hand-raised
- Hand-reared with siblings or socialized with other parrots as soon as weaned
- Hand-reared without siblings
- Wild-caught
- Unknown

Where did you get your parrot from?

Choose one of the following answers

- Pet store
- Breeder
- Organization that helps re-home parrots
- Private person
- Other:

Health check up

How often do you take your parrot to a veterinarian for general health check-up?

Choose one of the following answers

- 😃 - Two or more times per year
- 😕 - Once per year
- 🙁 - Less than once per year
- 😞 - Never went for a general check-up

How often do you have the opportunity to observe your parrot's behavior?

Choose one of the following answers

- 😍 - Several times (4+) throughout the day
- 😃 - 2-3 times per day
- 😕 - Once a day
- 🙁 - A few times per week
- 😞 - Once a week or less

Is the veterinarian who examines your parrot specialized in avian medicine?

Choose one of the following answers

- Yes

Yes

No

I don't know

Was any part of this section unclear or difficult to understand?

If so, please specify in the comment box below.

Was there any behaviour or type of information that you found difficult to observe or collect in order to answer the questions in this section?

If yes, please indicate which ones in the comment box below.

Section 2: Physical health

How to interpret your answers

😍: Great - 😃: Good - 😕: Room for improvement - 😟: Welfare concern -😩: Serious welfare concern

👩‍⚕️📞: Health problems that requires veterinarian intervention

🚨: Life threatening concern, need immediate veterinarian intervention

🔍: You need to know your parrot better. Take time to observe your parrot, either directly or by using a camera.

Please consult a behavioural consultant or veterinarian to review your assessment results and, if needed, develop an effective plan to improve your parrot's welfare.

Condition of flight and body feathers

What is the condition of your parrot's plumage?

Choose one of the following answers

😃 - **Intact with no damage**

😕 - **Mildly damaged or plucked:** coverts and/or down feathers are missing in focal areas, but most of the feathers are still intact (though some of these may show signs of damage)

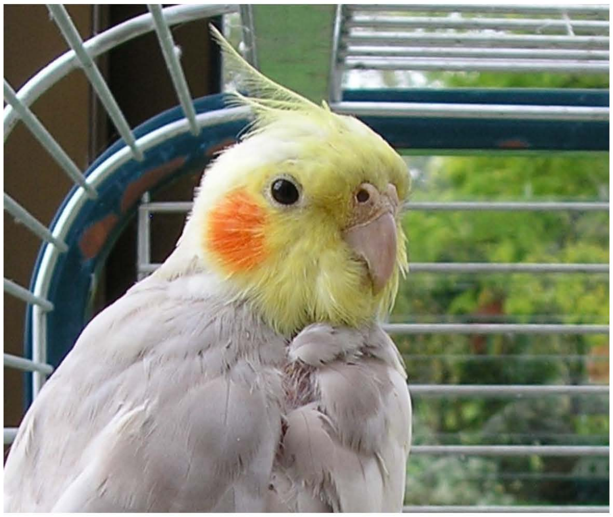

Photo by Jean-Loup Rault

😟 - **Moderately damaged or plucked:** coverts and/or down feathers are missing in several areas of the body leaving a patchy distribution or coverts are missing but down is still mostly or completely present

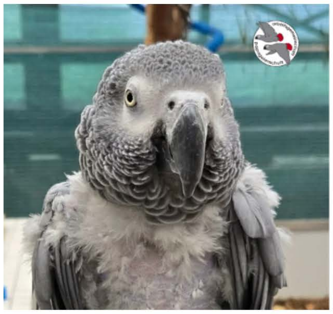

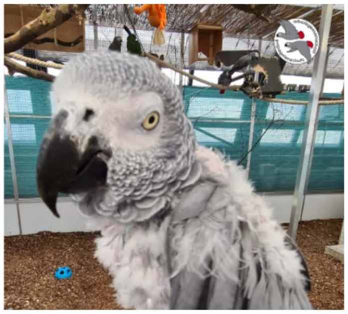

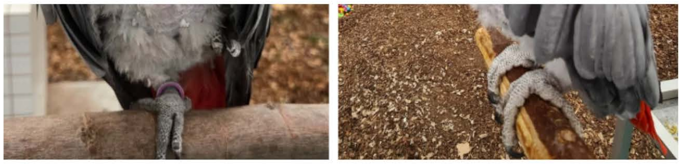

Photos by Andrea Piseddu (with permission from the Parrot Shelter of Vienna)

○ 😞 - **Severely damaged:** large areas of the body lack both coverts and down feathers, resulting in bare skin being clearly visible over multiple regions.

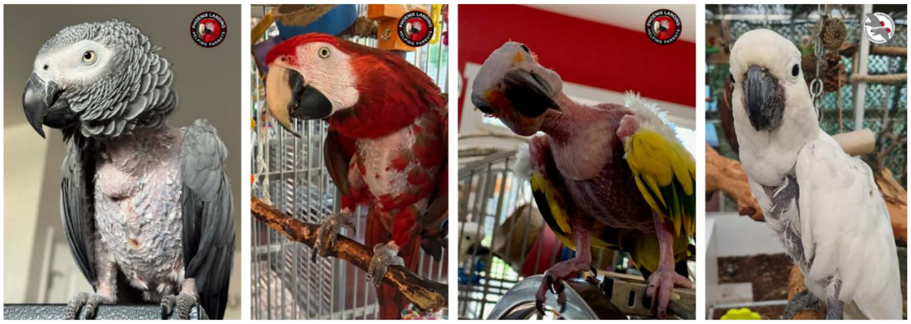

Photos by Ann Brooks (Phoenix Landing Foundation) and Andrea Piseddu (with permission from the Parrot Shelter of Vienna)

Number and appearance of droppings

Which statement best describes the droppings of your parrot?

❗ Choose one of the following answers

○ 😍 - My parrot's droppings are well-formed and of normal colour, with a chalky white urate portion and minimal odour. The number of droppings are within my parrot's normal elimination pattern.

○ 🙂 - My parrot's droppings are mostly within the normal range, though some occasional or slight changes in colour, odour, consistency or number can be observed.

○ 😟 - My parrot's droppings are excessive in number, and/or are discoloured, excessively watery and/or contain undigested food particles.

○ 👩‍⚕️📞 - My parrot's droppings are scant in number and/or volume, and contain fresh blood or have a tarry black colour.

○ 🔍 - I don't check droppings.

Pectoral muscle condition score

Please select the appropriate Bird Size-O-Meter score that best reflects your parrot's current muscle condition.

The Bird Size-O-Meter has been developed by UK Pet Food. For further details, please refer to the following link:https://www.ukpetfood.org/spotlight-on-obesity/how-to-identify-if-a-pet-needs-help/bird-size-o-meter-new.html

HOW TO CHECK YOUR BIRDS SHAPE

• Getting hands on is key. Not all birds are used to being handled but it is difficult to judge if your bird is the right weight by sight. You will need to gently feel your bird, using restraint if necessary.

• Use bare hands and not gloves to handle birds as then you can judge the tightness of grip. If you need to protect yourself use a cloth or towel.

• Small birds can be held in one hand with the neck between the first and second finger and the bird's back against the palm so that the wings and body are gently restrained in the closed hand.

• Larger parrots may take two people, one to hold the bird and the other to assess its body condition. A towel or cloth is used over the open hand to grasp the bird firmly behind its head and neck. The towel is then wrapped around the wings and body to prevent flapping. Gently stroking the top of the head and talking to the bird gently will help to calm it.

• Gently run your fingertips down the centre of the front of the bird in the midline over the breast area. You should be able to feel a bony ridge (known as the keel or breast bone). This should be easy to feel but not too prominent.

• Next, run your fingers at right angles to the keel across the breast muscles. If these feel shrunken so that the keel sticks out prominently your bird is too thin. If the breast muscles are just rounded but you can still feel the keel your bird is in good condition. If you cannot feel the keel and the muscles are very rounded or you can feel or see fat moving underneath the skin your bird is overweight.

• The breast muscle can also vary in size depending on how much exercise your bird gets so if it flies a lot it will have larger firmer breast muscles than a bird who does not fly. However, the same criteria still apply in assessing body condition prominence of the bony keel and presence of fat underneath the skin.

❗ Choose one of the following answers

○ I can't calculate the score because my parrot doesn't accept handling, and I don't feel comfortable insisting, it's too stressful and I'm afraid of hurting or scaring them.

○ 😞 - 1

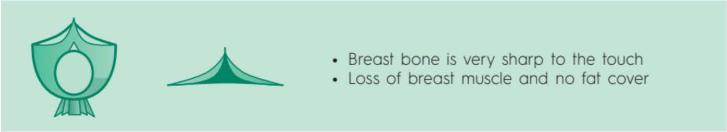

○ 😟 - 2

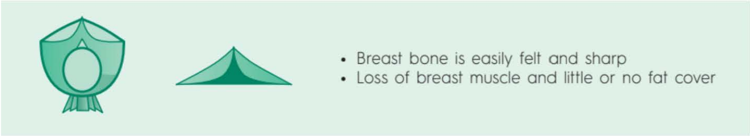

○ 🙂 - 3

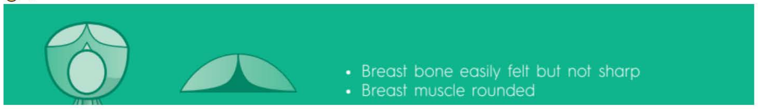

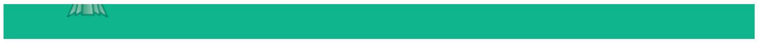

- 4

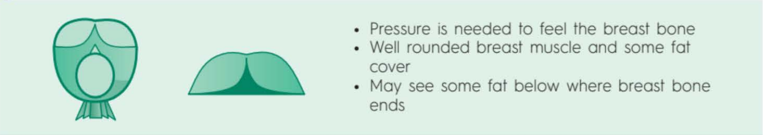

- 5

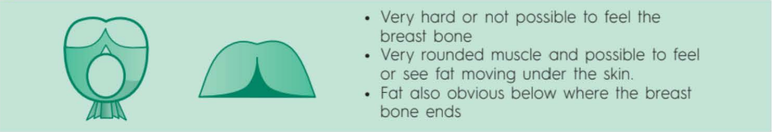

Presence of diagnosed diseases

Has your parrot been diagnosed with any disease by a veterinarian?

If yes, please indicate the disease(s) and any medication they are currently taking in the comment box

Choose one of the following answers

- No
- Yes

Please enter your comment here:

Signs of illness

Do you notice any of these conditions?

Some of the information was sourced from the Association of Avian Veterinarians: http://chrome-extension://efaidnbmnnnibpcajpcglclefindmkaj/https://cdn.ymaws.com/www.aav.org/resource/resmgr/pdf_2019/AAV_Signs-of-Illness-in-Comp.pdf

	- No	- Yes	- I am not sure
Redness, swelling or loss of feathers around eyes			
- Discharge from eyes, nares (nostrils), or mouth			
Crusty material in or around nares or flakiness on the skin or beak			
Overgrown beak and/or nails 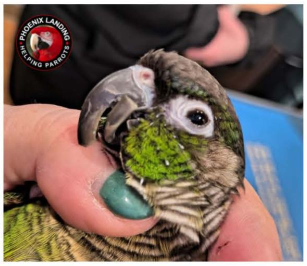 *Photo by Ann Brooks (Phoenix Landing Foundation)*			
Upper and lower beak not properly aligned 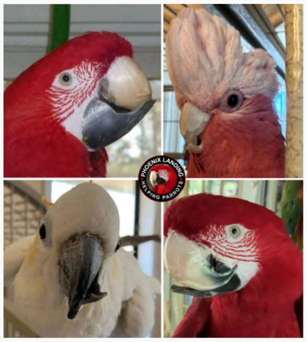 *Photos by Ann Brooks (Phoenix Landing Foundation)*			
- Vomiting or regurgitation			
- Signs of labored breathing, such as tail bobbing, open-mouth breathing, or abnormal respiratory sounds			
**Feather abnormalities** 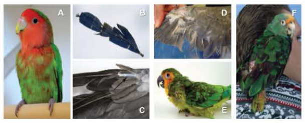			

**Feathers abnormalities -** **A** and **E**: discolouration of the feathers; **B** lines in the vane of a feather generally oriented perpendicular to the shaft; **C**: pin or blood feather (black arrow) **D**: abraded feathers; **F**: feather discolouration and depigmentation, overall dull- and ragged looking plumage. From: Plumage disorders in psittacine birds - part 1: feather abnormalities. van Zeeland, Y.R.A.; Schoemaker, N.J. (2014).			
🚨 - Signs of self-mutilation	○	○	○
🚨 - Blood loss or severe injury	○	○	○
🚨 - Fluffed posture, droopy wings, or sleeping more than usual	○	○	○
🚨 - Enlargement or swelling on the body	○	○	○
>10% increase or decrease of body weight within a week	○	○	○
🚨 - Inability to perch and/or sitting at the bottom of the cage	○	○	○
🚨 - Repeated straining, often with tail bobbing and/or fluffed posture	○	○	○
🚨 - Cloaca protrudes outside the body (prolapse)	○	○	○
Lameness or shifting of body weight	○	○	○
🚨 - Uncontrolled or irregular movement of the head, wings, or feet	○	○	○
Swelling, ulcers, or lesions under the feet	○	○	○

Vet diagnosis (follow up question for Signs of illness)

Were the condition(s) listed above diagnosed by a veterinarian?

Please answer this question only if your parrot exhibits any of the signs of illness listed in the previous table.

❗ Choose one of the following answers

○ Yes, all conditions

○ Only some conditions

○ No

Treatments for (follow up question for Signs of Illness)

Is your parrot currently receiving medication prescribed by a veterinarian for any of the conditions listed above?

Please answer this question only if your parrot exhibits any of the signs of illness listed in the previous table.

❗ Choose one of the following answers

○ 😃 - Yes, my parrot is receiving treatments prescribed by the veterinarian

○ 👩‍⚕️📞 - No, but my parrot is receiving over-the-counter medications

○ 👩‍⚕️📞 - No, my parrot is not receiving any medication

Was any part of this section unclear or difficult to understand?

If so, please specify in the comment box below.

Was there any behaviour or type of information that you found difficult to observe or collect in order to answer the questions in this section?

If yes, please indicate which ones in the comment box below.

Section 3: Housing and physical activity

How to interpret your answers

😍: Great - 😃: Good - 😕: Room for improvement - 😟: Welfare concern -😞: Serious welfare concern

🔍: You need to know your parrot better. Take time to observe your parrot, either directly or by using a camera.

Please consult a behavioural consultant or veterinarian to review your assessment results and, if needed, develop an effective plan to improve your parrot's welfare.

Enclosure type

Where is your parrot's enclosure located?

If your parrot has multiple enclosures, please specify all locations

Enclosure: space where the parrot sleeps, eats, drinks, and stays when it can't be supervised

	Main enclosure (space where the parrot stays most of time)	Secondary enclosure (additional space used occasionally)	Not applicable

Living room			
Hallway			
Kitchen			
Bedroom			
Garage			
Basement			
Parrot lives indoor and does not have a dedicated enclosure			
Room exclusively dedicated to the parrot			
Outdoor enclosure			
Other			

Enclosure size

Which statement most accurately describes the size of your parrot's main enclosure (where it resides most of the daytime) and its ability to move around?

For information on species wingspan measurements and recommended minimum cage sizes, please consult the following link: https://naturalinspirationsparrotcages.com/p/wingspan-info

Choose one of the following answers

- - My parrot has ample space to move, climb, hop and fly
- - My parrot has at least 2 wingspan of space in all directions to move, climb, and hop
- - My parrot is unable to fully extend its wings in one or more directions, with limited space to move

Enclosure material

What type of material is your parrot's enclosure made from?

Check all that apply

- - Stainless steel
- - Powder-coated metal
- - Wrought iron
- -Plastic-coated wire, zinc, wood or acrylic
- - I don't know
- Other: ____

Bars orientation

Please select the statement(s) that most accurately describe the bar orientation of your parrot's enclosure(s).

Check all that apply

- - The enclosure(s) has grid-patterned bars
- - The enclosure(s) has vertical bars
- - The enclosure(s) has horizontal bars
- -There are no bars to allow climbing (e.g. solid panels made of glass, acrylic)

Number of perches

How many perches are available in the parrot's enclosure(s)?

Choose one of the following answers

- - > 5
- - 3 – 5
- - 1 – 2
- - There are no perches

Perches' characteristics

Do you provide perches of...

	- Yes	- No
different sizes?		
different materials?		

Provision of opportunities that allow movement and climbing

Please indicate if your parrot receives the following types of enrichment that promote movement and climbing.

	Inside the enclosure			Outside the enclosure	
	😀 - Yes	😟 - No		😀 - Yes	😟 - No
Ropes	○	○		○	○
Swings	○	○		○	○
Ladders	○	○		○	○
Boings	○	○		○	○
Branches	○	○		○	○

Safety of enrichment material

Have you checked whether the material of the enrichment provided (perches, ropes, branches etc.) is safe for parrots?

Choose one of the following answers

- 😍 - Yes, I consulted my veterinarian or a behavioural consultant
- 😀 - Yes, I researched myself online, in books, or magazines
- 😟 - No

Presence of a retreating area/room to rest, sleep or withdraw

Does your parrot have unlimited access to an undisturbed area where it can rest, sleep or retreat from potential stressful situations (noise, presence of unfamiliar people, other animals etc.)?

Choose one of the following answers

- 😀 - Yes
- 😞 - No

Opportunity to spent time outside the enclosure

How often does your parrot spend time out of its enclosure ?

Choose one of the following answers

- 😍 - Most or all of its time (e.g. only in the enclosure when sleeping or when there is no human supervision)
- 😀 - Every day, for 3 hours or more
- 😕 - Every day, for 3 hours or less
- 😟 - Several times a week, but not every day
- 😞 - Never or less than once a week on average

Hygiene: frequency of cleaning food and water bowls, foraging toys, enclosure

How often do you clean the...

	😀 - Everyday or after every use	😟 - Several times a week, but not every day	😞 - Once a week or less
Enclosure (cage, room, aviary)?	○	○	○
Food bowl(s)?	○	○	○
Water bowl(s)?	○	○	○
Foraging toys?	○	○	○

Climate (temperature and humidity)

Do you regularly check the humidity and temperature to ensure they are appropriate for your parrot's species?

Choose one of the following answers

- 😀 - Yes, both
- 😕 - Only temperature
- 😕 - Only humidity
- 😟 - No

Air quality

How often do you refresh the air in the area where your parrot lives and spends most of its time?

Choose one of the following answers

- 😍 - More than once per day / I use an air purifier / My parrot lives outdoor
- 😀 - Once per day / I use an air purifier but is not constantly activated
- 😟 - Several times a week, but not every day
- 😞 - Once per week or less

Access to outdoor spaces

How often does your parrot spend time outdoor, if environmental circumstances permit (no risk of predation or mosquitoes' bites, appropriate temperature)?

Choose one of the following answers

- 😍 - At least once a day
- 🙂 - At least once a week
- 🙁 - Less than once a week on average
- 😞 - Never

Exposure to direct sunlight/UV light

How often do you expose your parrot to a UVA and UVB lamp or direct sunlight without a window in between?

Choose one of the following answers

- 😍 - Everyday
- 🙂 - Weekly
- 🙁 - Monthly
- 😞 - Never

Opportunity to spend time in high positions

Does your parrot have the opportunity to perch in high locations that are out of reach of people and other animals?

Choose one of the following answers

- 🙂 - Yes, both inside and outside of the enclosure
- 🙁 - Only inside the enclosure
- 🙁 - Only outside the enclosure
- 😞 - No

Ability to fly

How would you describe your parrot's flight ability?

If you are unsure of your parrot's flying ability, please select the answer "I don't know." Avoid attempting to assess it, as it could lead to injury or stress for your parrot.

Choose one of the following answers

- 🙂 - My parrot flies in all directions and maintains height during flight
- 😕 - My parrot flies in a downward trend and horizontally but cannot gain altitude
- 🙁 - My parrot attempts flying but is unable to do so and falls to the ground
- 😞 - My parrot does not fly or does not attempt to fly
- 🔍 - I don't know

Wing trim (follow up question for ability to fly) - Answer options will be accompanied by illustrations

Has your parrot received a wing trim?

Wing trimming is often regarded as unethical and a welfare concern, as it restricts parrots from performing natural behaviours such as flying. As a result, it is prohibited by law in several countries.

Choose one of the following answers

- 🙂 - No
- 🙁 - My parrot has received a bilateral skinny wing trim
- 😞 - My parrot has received a bilateral, transverse wing trim
- 😞 - My parrot has received a bilateral wing trim which includes the primaries and secondaries
- 😞 - My parrot has received a unilateral wing trim
- 😞 - My parrot has been permanently deflighted through a surgical procedure (e.g., pinioning)

Level of activity

How much time does your parrot spend each day moving, climbing, and flying?

Choose one of the following answers

- 😞 - 1 hour or less
- 🙁 - Between 1 and 2 hours
- 🙂 - Between 2 and 4 hours
- 😍 - 4 or more hours
- 🔍 - I don't know

Opportunity to fly

How often does your parrot have the opportunity to fly in a safe space?

Choose one of the following answers

- 😍 - Every day for 4 or more hours
- 😃 - Every day for less than 4 hours
- 😟 - Several times a week, but not every day
- 😣 - Never or less than once a week on average

Was any part of this section unclear or difficult to understand?

If so, please specify in the comment box below.

Was there any behaviour or type of information that you found difficult to observe or collect in order to answer the questions in this section?

If yes, please indicate which ones in the comment box below.

Section 4: Provision of enrichment and exploration

How to interpret your answers

😍: Great - 😃: Good - 🙂: Ok - 😕: Room for improvement - 😟: Welfare concern -😣: Serious welfare concern

🔍: You need to know your parrot better. Take time to observe your parrot, either directly or by using a camera.

Please consult a behavioural consultant or veterinarian to review your assessment results and, if needed, develop an effective plan to improve your parrot's welfare.

Provision of foraging and cognitive enrichment

How often do you provide the following types of enrichment?

	😍 - Every day	😃 - Weekly	😟 - Few times per month or less	😣 - Never
Certified chewable toys, cardboard or paper without ink, natural, not toxic and untreated cork and branches that can be safely chewed and destroyed				
Puzzles and problem solving games 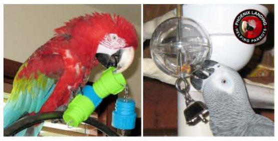 *Photos by Ann Brooks (Phoenix Landing Foundation)*				
Multiple food stations				
Scatter feeding (e.g. foraging mat, spreading food out in various locations) and foraging tray or box (food mixed with inedible items) 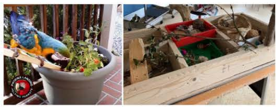 *Photos by Ann Brooks (Phoenix Landing Foundation)*				
Larger chunks of food or whole food items (with or without skewers)				
Non destructible puzzle feeders/foraging toys 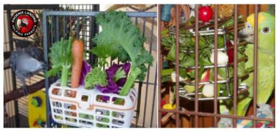 *Photos by Ann Brooks (Phoenix Landing Foundation)*				
Destructible foraging toys 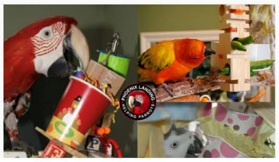				

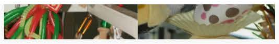 *Photos by Ann Brooks (Phoenix Landing Foundation)*				
Interactive toys that make sounds and/or move				
Auditory enrichment (e.g. radio, music)				
Visual enrichment (e.g. tv or tablets)				

Interaction with enrichment (follow up question for Provision of foraging and cognitive enrichment)

How does your parrot interact with the enrichment provided?

Examples of interaction: manipulation with beak and/or feet (e.g., chewing, grasping, exploring) or reactions to sound and visual cues.

Please answer only for the types of enrichment that you currently provide.

	😍 - Frequently and repeatedly uses with the enrichment, exploring and manipulating it extensively, showing continued interest until the item is destroyed (in case of destructible items) or food/contents is removed (if applicable)	🙂 - Shows brief interest in the enrichment and ignores it at other times	😟 - Does not use or interact with the enrichment at all, and completely ignores it	🔍 - I don't know
Certified chewable toys, carboard or paper without ink, natural, not toxic and untreated cork and branches that can be safely chewed and destroyed				
Puzzles and problem solving games				
Multiple food stations				
Scatter feeding (e.g. foraging mat, spreading food out in various locations) and foraging tray or box (food mixed with inedible items)				
Larger chunks of food or whole food items (with or without skewers)				
Non destructible puzzle feeders/foraging toys				
Destructible foraging toys				
Interactive toys that make sounds and/or move				
Auditory enrichment (e.g. radio, music)				
Visual enrichment (e.g. tv or tablets)				

Time spent foraging

How much time does your parrot spend daily foraging (i.e., searching for, procuring, and extracting food from enrichment that you provide)?

❗ Choose one of the following answers

- 😑 - My parrot does not forage
- 😟 - Less than 1 hour
- 🙂 - Between 1 and 2 hours
- 😀 - Between 2 and 4 hours
- 😍 - 4 hours or more
- 🔍 - I don't know

Provision of foraging enrichment in relation to the daily food ration

How is foraging enrichment provided for your parrot?

Foraging enrichment includes toys, devices, or other opportunities that encourage natural behaviors such as searching, procuring, and extracting food.

❗ Choose one of the following answers

- 😍 - For treats and more than half of the daily food ration
- 😀 - For treats and about half of the daily food ration
- 😕 - For treats and a small portion of of the daily food ration
- 😟 - Only for treats

Opportunities to select items based on preference (e.g., for colour, shape or type of material)

Do you select toys based on your parrot's preference for shape, size and colours?

❗ Choose one of the following answers

- 😍 - Yes
- 😕 - No

Enrichment replacement

How often do you replace toys/chewable items/ climbing enrichment by introducing new different one/s?

How often do you replace toys/chewable items/ climbing enrichment by introducing new different ones?

Choose one of the following answers

- 😍 - Every day
- 😃 - Weekly
- 😟 - Few times per month or less
- 😞 - Never

Response to novel objects

How does your parrot generally react towards unfamiliar objects (e.g. new toys, new home decoration) in its proximity?

Please note that the behavioural response could be linked to your parrot's personality. A negative behavioural response may not change, but you can help prevent it by avoiding the situations that trigger it.

Choose one of the following answers

- 😍 - My parrot actively approaches the object touching, chewing and manipulating it; certain objects may still trigger freezing or withdrawing responses.
- 😃 - My parrot appears interested in the object, and cautiously approach it; certain objects (e.g. larger furniture or electronic household devices) may still trigger freezing or withdrawing responses.
- 😟 - My parrot withdraws or freezes to most objects; certain objects (e.g. larger toys, larger furniture or electronic household devices) may trigger more freezing or withdrawing responses.
- 😞 - My parrot shows one or more of these behaviours: excessive or incessant screaming, frantic movements, flying away, falling of the perch. This response happens with any type of unfamiliar object.

Alertness

What is your parrot's current alertness level?

Choose one of the following answers

- 😍 - My parrot is very active and curious. It eagerly explores new objects, interacts frequently with people and toys, and responds quickly to its surroundings
- 😃 - My parrot responds to sounds, movements, and interactions. It engages with toys, interacts with people, and explores its environment with moderate enthusiasm
- 😟 - My parrot reacts occasionally but needs encouragement to engage. It shows some interest in its surroundings and sometimes participates in activities
- 🚨 - My parrot is mostly inactive or lethargic, shows little interest in its surroundings and does not respond much to things happening around it

Was any part of this section unclear or difficult to understand?

If so, please specify in the comment box below.

Was there any behaviour or type of information that you found difficult to observe or collect in order to answer the questions in this section?

If yes, please indicate which ones in the comment box below.

Section 5: Nutrition and maintenance behaviours

How to interpret the answers

😍: Great - 😃: Good - 😕: Room for improvement - 😟: Welfare concern -😞: Serious welfare concern

🔍: You need to know your parrot better. Take time to observe your parrot, either directly or by using a camera.

Please consult a behavioural consultant or veterinarian to review your assessment results and, if needed, develop an effective plan to improve your parrot's welfare.

Food Selectivity

Does your parrot eat all types of food that you provide?
Observing the animal while it eats is preferable, but checking the food bowl can also provide valuable insights.

Choose one of the following answers

- 😃 - Yes
- 😟 - No
- 🔍 - I don't know

Composition of the diet

Which of the following foods do you provide?

Please note that the appropriate amount of the foods listed depends greatly on your parrot's species, age, and individual needs. This table provides an overview of many different types of foods that can be included in a parrot's diet and may serve as a useful tool to discuss dietary choices with your veterinarian. Always consult a qualified avian vet to determine the most appropriate diet for your parrot.

🚩: Foods that should be offered only in limited amounts

⛔: Foods that should always be avoided

	Main component of the diet	Moderate to small quantities	As treat, training reward or vehicle for medications	Not provided
Pellet and/or other formulated food	○	○	○	○
Fresh vegetables	○	○	○	○
Fresh fruit	○	○	○	○
Sprouted seeds	○	○	○	○
Grains	○	○	○	○
Legumes	○	○	○	○
Tofu and/or other plant-based proteins	○	○	○	○
Seed mix	○	○	○	○
🚩- Nuts	○	○	○	○
Eggs	○	○	○	○
🚩 - Dairy products	○	○	○	○
⛔ - Other animal-based proteins	○	○	○	○
Nectar	○	○	○	○
⛔ - Processed food specifically designed for human consumption	○	○	○	○

Diet apropriateness

Have you checked whether the diet you provide is balanced and appropriate for your parrot's species?

❗ Choose one of the following answers

- ○ 😀 - Yes, I consulted my veterinarian or a behavioural consultant
- ○ 😕 - Yes, I researched by myself online, in books, or magazines
- ○ 😞 - No

Availability of fresh and clean water

How often do you change your parrot's water?

❗ Choose one of the following answers

- ○ 😍 - More than twice a day or as needed throughout the day
- ○ 😀 - Twice a day
- ○ 😕 - Once a day
- ○ 😟 - Less than once per day

Daytime spent sleeping / resting

What daytime does your parrot sleep / rest?

❗ Check all that apply

- ☐ Morning
- ☐ Afternoon
- ☐ Evening
- ☐ Night
- ☐ I don't know

Changes in food and/or water consumption

Have you observed a change in appetite or water intake by your parrot in the last 2 weeks?
i.e. you observed these changes directly observing the parrot's behaviour or by checking the amount of food or water consumed.

❗ Choose one of the following answers

- ○ 😀 - No
- ○ 😞 - Yes
- ○ 🔍 - I don't know

Changes of resting-sleeping patterns

Have you noticed any changes in the sleeping pattern of your parrot in the last two weeks?
e.g. sleeping more than usual or at times of the day when it typically does not sleep.

❶ Choose one of the following answers

- 😃 - No
- 😞 - Yes
- 🔍 - I don't know

Opportunity to bathe
Please indicate how often you provide the following bathing opportunities for your parrot

	Every day	Weekly	Few times per month or less	Never
Mist	○	○	○	○
Shower	○	○	○	○
Shallow dish or tray	○	○	○	○
Other	○	○	○	○

Interest in bathing

How does your parrot bathe when you provide the opportunity to do so?

❶ Choose one of the following answers

- 😍 - My parrot always bathes on its own
- 😃 - My parrot bathes on its own but sometimes needs encouragement to do so
- 😕 - My parrot always needs encouragement to bathe
- 😟 - My parrot doesn't bathe, and I don't try to change that
- 😞 - My parrot tries to avoid bathing, but I make sure it gets bathed anyway
- 🔍 - I don't know

Beak maintenance

Does your parrot engage in self-maintenance behaviors for its beak?

e.g. rubbing it against hard objects, grinding it by making a rhythmic motion with the upper and lower mandibles, or using bars or other objects to clean the inside of its beak

❶ Choose one of the following answers

- 😃 - Yes
- 😞 - No
- 🔍 - I don't know

Changes in preening activity

Have you noticed any changes in your parrot's self-preening behavior (such as using its beak to clean, arrange, or maintain its feathers) over the past month?

❶ Choose one of the following answers

- 😃 - No
- 😞 - Yes
- 🔍 - I don't know

Was any part of this section unclear or difficult to understand?

If so, please specify in the comment box below.

Was there any behaviour or type of information that you found difficult to observe or collect in order to answer the questions in this section?

If yes, please indicate which ones in the comment box below.

Section 6: Social and reproductive behaviours

How to interpret your answers

😍: Great - 😃: Good - 😕: Room for improvement - 😟: Welfare concern -😞: Serious welfare concern

Please consult a behavioural consultant or veterinarian to review your assessment results and, if needed, develop an effective plan to improve your parrot's welfare.

Social housing (alone vs pair vs group)

Please select the option that best describes your parrot's social contact with other parrot.

Please note that parrots are highly social animals, and living in group is important for their welfare. However, introducing new individuals should always be carefully evaluated with an expert to ensure it is suitable and safe for all parrots involved.

Choose one of the following answers

- My parrot is housed together with two or more other parrots and can continuously interact with them
- My parrot is housed together with one parrot and they can continuously interact
- My parrot can physically interact with one or more parrots at certain times of the day (or week), but is not continuously housed together with other parrots
- My parrot is housed individually, but is able to see other parrots and can vocally interact with them
- My parrot lives alone and does not have any type of contact with other parrots

Social behaviours

How frequently do these social interactions occur?

	🙂 - On all or most encounters	😕 - On some encounters	🙁 - Rarely to never
Staying right beside another parrot			
Feeding alongside another parrot			
Preening another parrot			
Being preened by another parrot			
Vocal interaction with another parrot (contact calls)			

	🙂 - Rarely to never	😕 - On some encounters	🙁 - On all or most encounters
Mating with another parrot			
Regurgitating food for other parrot			
Having food regurgitated by another parrot			
Stalking (i.e. following or watching insistently) another parrot			
Stalked (i.e. followed or watched insistently) by another parrot			
Attacks (biting, lunging) another parrot			
Being attacked (bitten or lunged at) by another parrot			

Reproductive behaviours

How frequently do you observe these behaviors?

	🙂 - Never	😕 - Few times per month or less	🙁 - Weekly	😣 - Every day
Rubbing its cloaca (vent area) against objects such as perches, cage bars, toys				
Actively seeking or spending time in dark, enclosed, or secluded spaces such us underneath furniture, inside drawers or cabinets, behind cushions, or in boxes or clothing.				
Lunging, biting, vocalizing aggressively, or chasing any-one who approaches or gets too close to a specific area (e.g. cage or nest)				
Egg laying				
Regurgitating food and directing it towards its own body parts (e.g. its foot)				
Regurgitating food on a special toy or item in the cage				

Was any part of this section unclear or difficult to understand?

If so, please specify in the comment box below.

Was there any behaviour or type of information that you found difficult to observe or collect in order to answer the questions in this section?

If yes, please indicate which ones in the comment box below.

Section 7: Parrot-Human interactions

How to interpret your answers

😍: Great - 😃: Good - 🙂: Ok - 😕: Room for improvement - 😟: Welfare concern -😖: Serious welfare concern

🔍: You need to know your parrot better. Take time to observe your parrot, either directly or by using a camera.

Please consult a behavioural consultant or veterinarian to review your assessment results and, if needed, develop an effective plan to improve your parrot's welfare.

Types of interaction with humans (table 1)

How often do you allow your parrot to engage in the following behaviors?

	😃 - Never	😕 - Few times per month or less	😟 - Weekly	😖 - Every day
Sitting on shoulder, lap, or another part of the body	○	○	○	○
Gently nibbling or grooming hair, beard, eyelashes, or skin with its beak (preening)	○	○	○	○
Crawling under clothes or blankets	○	○	○	○

Time spent in presence of humans

How many hours per day is your parrot surrounded by you and/or familiar people (e.g. partner, family member)?

❶ Choose one of the following answers

- ○ 😖 - <1h
- ○ 😕 - It varies greatly per day
- ○ 🙂 - 2h – 4h
- ○ 😃 - 4h - 8h
- ○ 😍 - > 8h
- ○ 😟 - All time, including night

Types of interaction with humans (table 2)

How often do the following interactions take place with the parrot?

	😍 - Every day	😃 - Weekly	😕 - Few times per month or less	😟 - Never
Petting the parrot's head, cheeks and/or neck	○	○	○	○
Gently responding to the parrot's vocalizations	○	○	○	○
Talking to the parrot	○	○	○	○
Playing music for my parrot	○	○	○	○
Taking the parrot along when going out	○	○	○	○
Training the parrot	○	○	○	○

	😃 - Never	😕 - Few times per month or less	😟 - Weekly	😖 - Every day
Petting the parrot under the wings	○	○	○	○
Petting the parrot's chest	○	○	○	○
Petting the parrot's back and tail	○	○	○	○
Holding and shaking the parrot's beak while playing	○	○	○	○
Yelling at the parrot	○	○	○	○
Kissing the parrot	○	○	○	○
Providing food by mouth or allowing the parrot to eat from the mouth	○	○	○	○
Pressing on parrot's chest to encourage it to step up onto hands, arms, or an offered perch	○	○	○	○
Holding the parrot's body in the hands (with or without gloves or a towel)	○	○	○	○
Grabbing the parrot with a net or a towel	○	○	○	○

Parrot response to training (follow up question if the owner indicates they train their parrot)

How does your parrot respond during training session?

❶ Choose one of the following answers

- ○ 😍 - Very focused and responds quickly
- ○ 😃 - Mostly focused, but gets distracted sometimes
- ○ 🙂 - Responds sometimes, but often gets distracted
- ○ 😕 - Rarely focused, responds only occasionally
- ○ 😟 - Does not respond or shows no interest in training

Behaviours directed towards humans

How frequently does your parrot display the behavior below towards humans?

	😍 - Every day	😀 - Weekly	🙂 - Monthly	😕 - Rarely to never
Offering the head/neck to be petted				
Contact calls/vocalizations				
Crouching with the head down				
Bowing and bobbing the body				
Voluntarily steps up onto your hand, arm, or an offered perch without encouragement				

	😀 - Rarely to never	😕 - Monthly	😟 - Weekly	😣 - Everyday
Begging for food (raise its wings, flutter them and bob its head up, and down in a rhythmic pattern)				
Regurgitating food				
Masturbation (rubbing the cloaca against humans)				
Approaching a person and biting				

Human selectivity (follow up question for Behaviours directed towards humans)

To how many people does your parrot display each of the behaviour(s) selected above?

Please answer this question only if your parrot exhibits any of the behaviors listed in the previous table every day, weekly, or monthly.

	To everyone	To few people	Only to me
Offering the head/neck to be petted			
Contact calls/vocalizations			
Crouching with the head down			
Bowing and bobbing the body			
Voluntarily steps up onto your hand, arm, or an offered perch without encouragement			
Begging for food (raise its wings, flutter them and bob its head up, and down in a rhythmic pattern)			
Regurgitating food			
Masturbation (rubbing the cloaca against humans)			
Moving toward a person and biting			

Response upon contact with caregiver

How does your parrot respond to you as the caregiver?

	😀 - Every or most times	😕 - Sometimes	😣 - Rarely to never	🔍 - I don't know
My parrot accepts my presence near the enclosure or stand				
My parrot accepts physical contact initiated by me				
My parrot initiates physical contact itself by actively approaching me				

	😀 - Rarely to never	😟 - Sometimes	😣 - Every or most times	🔍 - I don't know
My parrot stays completely still and does not react when I am approaching it				
My parrot tries to lunge, attempt to bite or chase me when I am approaching it				
My parrot tries to escape and avoid contact when I am approaching it				

Response upon contact with familiar person

How does your parrot respond to a familiar person (e.g. partner, family member, friend)?

	😍 - Every or most times	😀 - Sometimes	😕 - Rarely to never	🔍 - I don't know
My parrot accepts their presence near the enclosure or stand				
My parrot accepts physical contact initiated by them				
My parrot initiates physical contact itself by actively approaching them				

	😀 - Rarely to never	😕 - Sometimes	😟 - Every or most times	🔍 - I don't know
My parrot stays completely still and does not react when they are approaching it				
My parrot tries to lunge, attempt to bite or chase them when they are approaching it				

My parrot tries to escape and avoid contact when they are approaching it				

Comfort behaviour around humans

Does your parrot eat, drink, rest and/or clean its feathers...

Please note that the behavioural response could be linked to your parrot's personality. A negative behavioural response may not change, but you can help prevent it by avoiding the situations that trigger it.

	- Yes	- No	- I don't know
In your presence?			
in the presence of all household members?			
in presence of people that come by regurarly?			
in presence of people that come by infrequently?			

Does your parrot spend most of its time in high locations out of human reach...

Please note that the behavioural response could be linked to your parrot's personality. A negative behavioural response may not change, but you can help prevent it by avoiding the situations that trigger it.

	- No	- Yes	- I don't know
In your presence?			
in the presence of all household members?			
in presence of people that come by regurarly?			
in presence of people that come by infrequently?			

Was any part of this section unclear or difficult to understand?

If so, please specify in the comment box below.

Was there any behaviour or type of information that you found difficult to observe or collect in order to answer the questions in this section?

If yes, please indicate which ones in the comment box below.

Section 8: Abnormal and fear-related behaviours

How to interpret your answers

: Great - : Good - : Room for improvement - : Welfare concern - : Serious welfare concern

: You need to know your parrot better. Take time to observe your parrot, either directly or by using a camera.

Please consult a behavioural consultant or veterinarian to review your assessment results and, if needed, develop an effective plan to improve your parrot's welfare.

Expression of avoidance and escape behaviours

When does your parrot display any of the following behaviors?

Please note that the behavioural response could be linked to your parrot's personality. A negative behavioural response may not change, but you can help prevent it by avoiding the situations that trigger it.

The answer options range from the worst conditions at the top to the best conditions at the bottom

	Tremors or shivering, freezing, hiding, withdrawing	Attempting to escape by flying or moving away, possibly falling off the perch, screeching/high-pitched screams
- Never		
- Mostly in response to specific or uncommon situations (e.g. visit to the vet, sudden loud noise)		
- Mostly when exposed to a certain situation, outside of its daily environment (e.g. outdoor activity, visit of a new place)		
- Predominantly when exposed to changes in its daily environment (e.g. presence or approach by new, unfamiliar people/guests or animals, change of furniture, new house decorations, provision of new toys)		
- Most of the time, even in its daily environment		
- I don't know		

Disruptive vocalization or screams

How often does your parrot produce disruptive, loud vocalizations or screams?

[illegible]

Choose one of the following answers

- 😍 - Never
- 🙂 - Rarely and mostly limited to specific moments of the day (e.g. morning and evening) or in response to specific and uncommon situations (e.g. sudden loud noise)
- 😕 - Occasionally and mostly when exposed to specific contexts (e.g. when left alone, presence of unfamiliar people/guests)
- 😟 - Frequently, and incessant screaming can occur but mostly in specific contexts (e.g. when left alone, presence of unfamiliar people/guests)
- 😑 - For the majority of the day, and sometimes incessantly for hours with no apparent reason or cause

Abnormal, sham and excessive behaviours

Does your parrot exhibit any of the following behaviors?

Please note that interpreting abnormal behaviours can be challenging. If you observe any of the behaviours in the table, seek expert guidance to evaluate whether they reflect a welfare issue.

	😑 - Yes	🙂 - No	🔍 - I am not sure
Pacing: repetitive walking back and forth along a fixed path	○	○	○
Route tracing: repeatedly follow the same path or pattern within its enclosure, such as moving along a specific perch, climbing the same section of the cage, or flying in a predictable loop	○	○	○
Swaying and rocking: repeatedly shift its body side to side or back and forth in a rhythmic motion	○	○	○
Tongue rolling and flicking: repetitive quick movement of the tongue or rolling or flicking of the tongue inside or out-side the beak	○	○	○
Beak clacking: rapid, repetitive, excessive or compulsive clicking or clacking of the beak	○	○	○
Beak rubbing: repeatedly rubbing the beak against sur-faces such as perches or bars	○	○	○
Repetitive licking: repeated licking of surfaces such as cage bars, perches, or walls	○	○	○
	😑 - Yes	🙂 - No	🔍 - I am not sure
Spot pecking: repetitively peck at a specific spot, such as a cage bar, perch, wall, or even an imaginary point	○	○	○
Sham bathing: mimic bathing motions without the pres-ence of water or an appropriate bathing substrate	○	○	○
Sham chewing and chewing not chewable items: mimics chewing motions or engages with non-chewable objects (e.g., metal bars or plastic) or surfaces	○	○	○
Toe-nail biting: repeatedly bite or nibble at their own toes or nails	○	○	○
Feeding objects (e.g. mirrors, toys)	○	○	○

Was any part of this section unclear or difficult to understand?

If so, please specify in the comment box below.

Was there any behaviour or type of information that you found difficult to observe or collect in order to answer the questions in this section?

If yes, please indicate which ones in the comment box below.

Feedback on the Welfare Assessment Tool

Which country are you currently living in?

How did you find the length of the assessment?

If you have any additional thoughts or suggestions, please share them in the comment box below.

Choose one of the following answers

- It was too long and felt tiring or overwhelming to complete.
- A bit lengthy, but definitely useful and worth completing.
- The length was appropriate and well-balanced.

Please enter your comment here:

It felt a bit short, I expected more questions.

Were there any questions you felt were missing from the tool?

If yes, please specify them in the comment box below.

Do you think you would use this tool regularly to monitor your parrot’s welfare?

If you have any additional thoughts or suggestions, please share them in the comment box below.

Choose one of the following answers

- Yes, definitely, it would be a valuable resource to keep track of my parrot’s welfare.
- Yes, to some extent, I might use it occasionally, depending on the situation.
- Maybe, I would need more time or repeated use to decide.
- Probably not, I don’t think I would find it useful in my daily routine.

Please enter your comment here:

Submit

Parrot Welfare Assessment Tool: Experts Review

Information and Consent

vetmeduni University of Veterinary Medicine, Vienna — Utrecht University

Dear expert,
Thank you for taking part in this survey!
The aim of this survey is to review the prototype of a parrot welfare assessment tool for parrot owners, developed based on the results of 10 online focus group meetings with parrot experts.
The tool consists of several questions addressing different aspects of parrot welfare divided into eight sections.

Each question is accompanied by an emoji-based feedback system designed to help owners interpret the results. In this version, the feedback appears alongside each question to support your evaluation. However, in the final version of the tool, feedback will be shown only at the end of the assessment, not during the answering process.
At the beginning of each section, you will find a legend explaining how to interpret the emojis used in the feedback system.
Some questions are also accompanied by illustrative images to clarify their meaning. While some of these images are already included, others are currently missing. We are still in the process of identifying suitable visuals to add later.

Please note: You are not required to answer the welfare assessment questions yourself. These are the questions that parrot owners will respond to assess their bird's welfare.
Your task is to review the questions and provide feedback on their **clarity** and **validity** at the end of each section. At the end of the assessment, there is a final section where you can provide an overall feedback on the tool. **Please complete this survey by 15.06.2025.**

You have the **opportunity to save partially finished surveys** by clicking on the "Resume later" button located at the top right of every page. After clicking this button, you will be asked to create a username and a password and to provide your email address. These credentials will allow you to return to your partially completed survey. Once you complete this step, you will receive a new link by email. **To resume the survey, please use only the new link** provided in that email and log in with the username and password you created.

Please note that:

- Participation is voluntary and anonymous.
- You can withdraw at any moment of the study.
- You have to be at least 18 years old to participate.
- The results of this survey will be included in a PhD thesis and published in a scientific journal.
- Your name will not appear in any published documents related to this study, and your statements will remain anonymous.
- Your personal data will be treated in compliance with the European General Data Protection Regulation and will be stored only as long as needed for the purpose of this study.

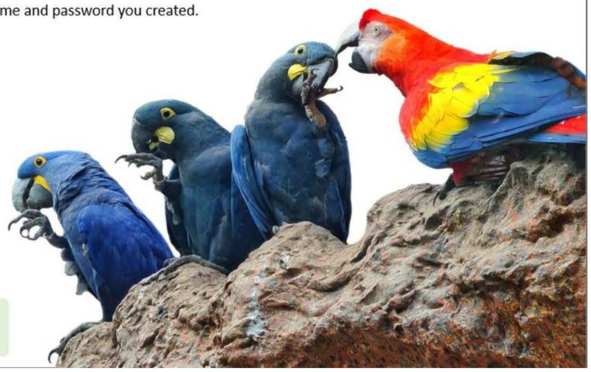

If you have any questions or concerns, please do not hesitate to contact the responsible person of this study:

Andrea Piseddu
Centre for animal Nutrition and Welfare, University of Veterinary Medicine Vienna, Vienna, Veterinaerplatz 1, 1210 Vienna
Andrea.Piseddu@vetmeduni.ac.at

By clicking the button "**Next**" and submitting your responses to the survey you are confirming to have read and understood the above statements and give your informed consent.

This survey is anonymous.

The record of your survey responses does not contain any identifying information about you, unless a specific survey question explicitly asked for it.

If you used an identifying access code to access this survey, please rest assured that this code will not be stored together with your responses. It is managed in a separate database and will only be updated to indicate whether you did (or did not) complete this survey. There is no way of matching identification access codes with survey responses.

Welcome to the Parrot Welfare Assessment Tool

This tool is designed to help caregivers evaluate the welfare of companion parrots (order *Psittaciformes*). It includes 77 questions divided into 8 sections, covering your parrot's physical condition, behaviour, and the care and management practices that may influence its welfare.

When answering the questions, base your responses on what has happened in the past month, unless a different time frame is specified. To answer some questions, it is important to be in front of your parrot, as direct observation may be necessary. The tool can be repeated monthly to help you monitor your parrot's welfare over time and detect any changes that may require attention.

Please note that this tool:

- is intended to provide a general overview of your parrot's welfare. It does not offer specific recommendations or diagnoses. Every parrot is unique, and interpreting welfare concerns can be complex.
- is not designed to assess the welfare of chicks or parrots kept primarily for breeding purposes.

If the results of the assessment highlight any welfare concerns or areas that could be improved, you may need to consider making some changes in your parrot's environment or daily care. Before taking action, it's important to consult with a qualified expert, such as a veterinarian experienced in avian care or a certified parrot behaviour consultant. Their support is essential to ensure that any changes you make are appropriate, effective, and tailored to your parrot's individual needs.

Thank you for taking the time to reflect on your parrot's well-being. Your commitment is an important step toward to promote a better quality of life for your feathered companion.

Section 1: General information

How to interpret your answers:

😍: Great - 😀: Good - 😕: Room for improvement - 😟: Welfare concern - 😞: Serious welfare concern

Please consult a behavioural consultant or veterinarian to review your assessment results and, if needed, develop an effective plan to improve your parrot's welfare.

Name of your parrot

Species of your parrot

Sex

Choose one of the following answers

- ◯ Female
- ◯ Male
- ◯ Unknown

How old is your parrot?

How long have you been living with your parrot?

Where did you get your parrot from?

Choose one of the following answers

- ◯ Pet store
- ◯ Breeder
- ◯ Organization that helps re-home parrots
- ◯ Private person
- ◯ Other:

Rearing history

Please select the option that best describes your parrot's rearing history.

Choose one of the following answers

- ◯ Parent-reared and briefly socialized with humans for short amounts of time each week
- ◯ Parent-reared
- ◯ Initially parent-reared and later hand-raised
- ◯ Hand-reared with siblings or socialized with other parrots as soon as weaned
- ◯ Hand-reared without siblings
- ◯ Wild-caught
- ◯ Unknown

Health check up

How often do you take your parrot to a veterinarian for general health check-up?

Choose one of the following answers

- ◯ - Two or more times per year
- ◯ - Once per year
- ◯ - Less than once per year
- ◯ - Never went for a general check-up

How often do you have the opportunity to observe your parrot's behavior?

Choose one of the following answers

- ◯ - Several times (4+) throughout the day
- ◯ - 2-3 times per day
- ◯ - Once a day
- ◯ - A few times per week
- ◯ - Once a week or less

Is the veterinarian who examines your parrot specialized in avian medicine?

Choose one of the following answers

- ◯ Yes

Yes

No

I don't know

Was any information in this section inaccurate or incorrect?

If so, please specify in the comment box below.

Was any part of this section unclear or difficult to understand?

If so, please specify in the comment box below.

Section 2: Physical health

How to interpret your answers

😍: Great - 😃: Good - 😕: Room for improvement - 😟: Welfare concern -😣: Serious welfare concern

👩‍⚕️📞: Health problems that requires veterinarian intervention

🚨: Life threatening concern, need immediate veterinarian intervention

🔍: You need to know your parrot better. Take time to observe your parrot, either directly or by using a camera.

Please consult a behavioural consultant or veterinarian to review your assessment results and, if needed, develop an effective plan to improve your parrot's welfare.

Condition of flight and body feathers

What is the condition of your parrot's plumage?

Choose one of the following answers

😃 - **Intact with no damage**

😕 - **Mildly damaged or plucked:** coverts and/or down feathers are missing in focal areas, but most of the feathers are still intact (though some of these may show signs of damage)

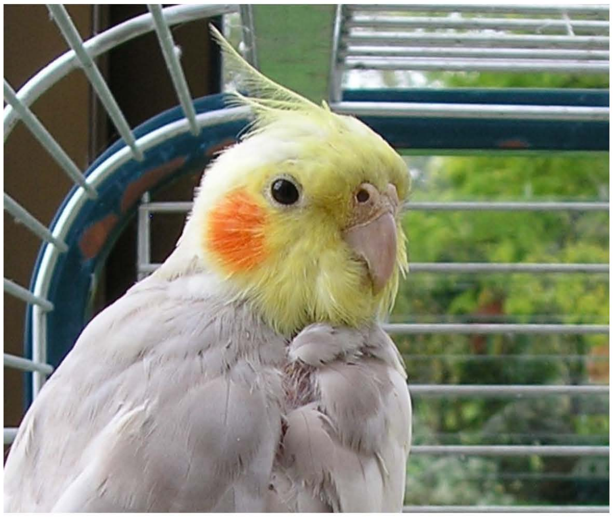

Photo by Jean-Loup Rault

😟 - **Moderately damaged or plucked:** coverts and/or down feathers are missing in several areas of the body leaving a patchy distribution or coverts are missing but down is still mostly or completely present

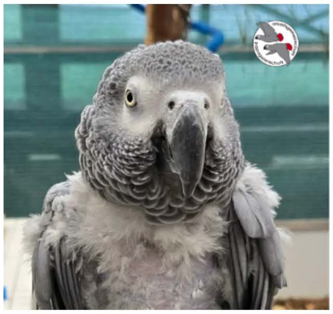

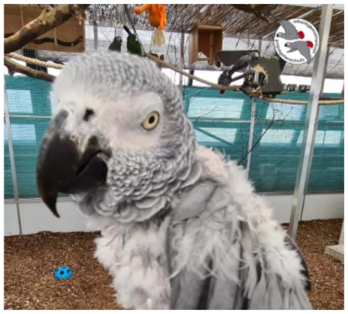

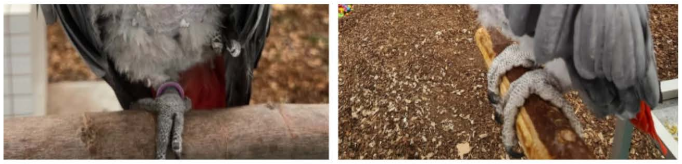

Photos by Andrea Piseddu (with permission from the Parrot Shelter of Vienna)

- 😞 - **Severely damaged:** large areas of the body lack both coverts and down feathers, resulting in bare skin being clearly visible over multiple regions.

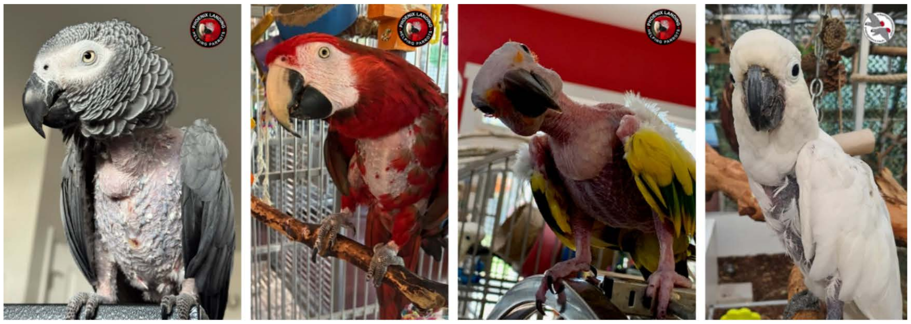

Photos by Ann Brooks (Phoenix Landing Foundation) and Andrea Piseddu (with permission from the Parrot Shelter of Vienna)

Number and appearance of droppings

Which statement best describes the droppings of your parrot?

❗ Choose one of the following answers

- 😍 - My parrot's droppings are well-formed and of normal colour, with a chalky white urate portion and minimal odour. The number of droppings are within my parrot's normal elimination pattern.
- 🙂 - My parrot's droppings are mostly within the normal range, though some occasional or slight changes in colour, odour, consistency or number can be observed.
- 😟 - My parrot's droppings are excessive in number, and/or are discoloured, excessively watery and/or contain undigested food particles.
- 👩‍⚕️📞 - My parrot's droppings are scant in number and/or volume, and contain fresh blood or have a tarry black colour.
- 🔍 - I don't check droppings.

Pectoral muscle condition score

Please select the appropriate Bird Size-O-Meter score that best reflects your parrot's current muscle condition.

The Bird Size-O-Meter has been developed by UK Pet Food. For further details, please refer to the following link:https://www.ukpetfood.org/spotlight-on-obesity/how-to-identify-if-a-pet-needs-help/bird-size-o-meter-new.html

HOW TO CHECK YOUR BIRDS SHAPE

• Getting hands on is key. Not all birds are used to being handled but it is difficult to judge if your bird is the right weight by sight. You will need to gently feel your bird, using restraint if necessary.

• Use bare hands and not gloves to handle birds as then you can judge the tightness of grip. If you need to protect yourself use a cloth or towel.

• Small birds can be held in one hand with the neck between the first and second finger and the bird's back against the palm so that the wings and body are gently restrained in the closed hand.

• Larger parrots may take two people, one to hold the bird and the other to assess its body condition. A towel or cloth is used over the open hand to grasp the bird firmly behind its head and neck. The towel is then wrapped around the wings and body to prevent flapping. Gently stroking the top of the head and talking to the bird gently will help to calm it.

• Gently run your fingertips down the centre of the front of the bird in the midline over the breast area. You should be able to feel a bony ridge (known as the keel or breast bone). This should be easy to feel but not too prominent.

• Next, run your fingers at right angles to the keel across the breast muscles. If these feel shrunken so that the keel sticks out prominently your bird is too thin. If the breast muscles are just rounded but you can still feel the keel your bird is in good condition. If you cannot feel the keel and the muscles are very rounded or you can feel or see fat moving underneath the skin your bird is overweight.

• The breast muscle can also vary in size depending on how much exercise your bird gets so if it flies a lot it will have larger firmer breast muscles than a bird who does not fly. However, the same criteria still apply in assessing body condition prominence of the bony keel and presence of fat underneath the skin.

❗ Choose one of the following answers

- I can't calculate the score because my parrot doesn't accept handling, and I don't feel comfortable insisting, it's too stressful and I'm afraid of hurting or scaring them.
- 😞 - 1

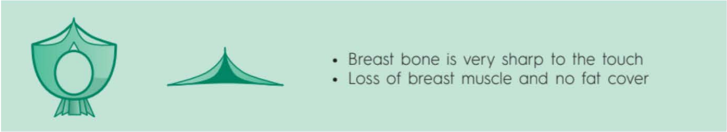

- 😟 - 2

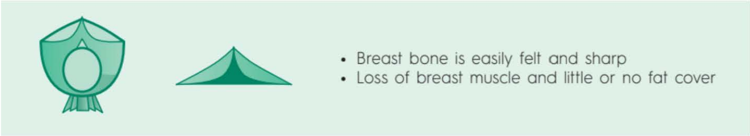

- 🙂 - 3

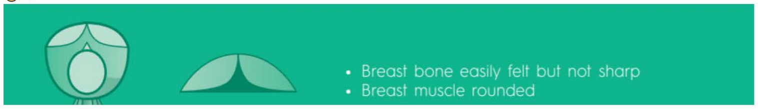

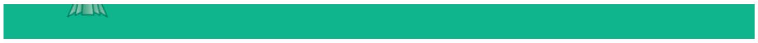

- 4

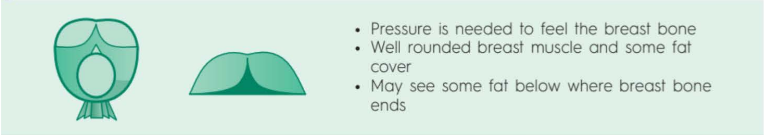

- 5

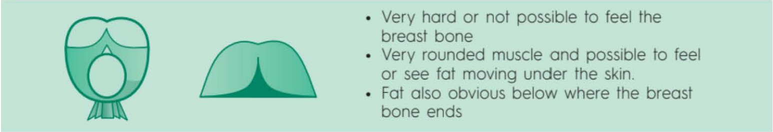

Presence of diagnosed diseases

Has your parrot been diagnosed with any disease by a veterinarian?

If yes, please indicate the disease(s) and any medication they are currently taking in the comment box

Choose one of the following answers

- No
- Yes

Please enter your comment here:

Signs of illness

Do you notice any of these conditions?

Some of the information was sourced from the Association of Avian Veterinarians: http://chrome-extension://efaidnbmnnnibpcajpcglclefindmkaj/https://cdn.ymaws.com/www.aav.org/resource/resmgr/pdf_2019/AAV_Signs-of-Illness-in-Comp.pdf

	- No	- Yes	- I am not sure
Redness, swelling or loss of feathers around eyes			
- Discharge from eyes, nares (nostrils), or mouth			
Crusty material in or around nares or flakiness on the skin or beak			
Overgrown beak and/or nails 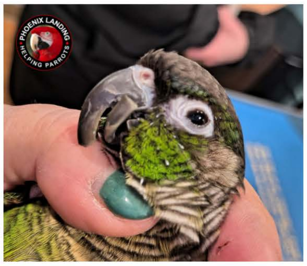 *Photo by Ann Brooks (Phoenix Landing Foundation)*			
Upper and lower beak not properly aligned 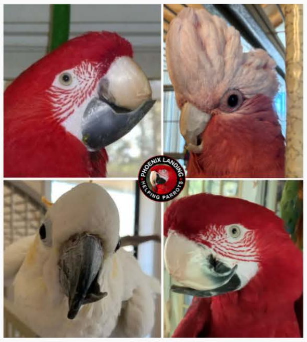 *Photos by Ann Brooks (Phoenix Landing Foundation)*			
- Vomiting or regurgitation			
- Signs of labored breathing, such as tail bobbing, open-mouth breathing, or abnormal respiratory sounds			
**Feather abnormalities** 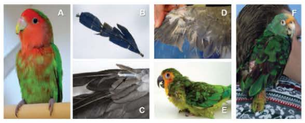			

**Feathers abnormalities** - **A** and **E**: discolouration of the feathers; **B** lines in the vane of a feather generally oriented perpendicular to the shaft; **C**: pin or blood feather (black arrow) **D**: abraded feathers; **F**: feather discolouration and depigmentation, overall dull- and ragged looking plumage. From: Plumage disorders in psittacine birds - part 1: feather abnormalities. van Zeeland, Y.R.A.; Schoemaker, N.J. (2014).			
🚨 - Signs of self-mutilation			
🚨 - Blood loss or severe injury			
🚨 - Fluffed posture, droopy wings, or sleeping more than usual			
🚨 - Enlargement or swelling on the body			
>10% increase or decrease of body weight within a week			
🚨 - Inability to perch and/or sitting at the bottom of the cage			
🚨 - Repeated straining, often with tail bobbing and/or fluffed posture			
🚨 - Cloaca protrudes outside the body (prolapse)			
Lameness or shifting of body weight			
🚨 - Uncontrolled or irregular movement of the head, wings, or feet			
Swelling, ulcers, or lesions under the feet			

Vet diagnosis (follow up question for Signs of illness)

Were the condition(s) listed above diagnosed by a veterinarian?

❗ Choose one of the following answers

- Yes, all conditions
- Only some conditions
- No

Treatments for (follow up question for Signs of Illness)

Is your parrot currently receiving medication prescribed by a veterinarian for any of the conditions listed above?

❗ Choose one of the following answers

- 😃 - Yes, my parrot is receiving treatments prescribed by the veterinarian
- 👩‍⚕️📞 - No, but my parrot is receiving over-the-counter medications
- 👩‍⚕️📞 - No, my parrot is not receiving any medication

Was any information in this section inaccurate or incorrect?

If so, please specify in the comment box below.

Was any part of this section unclear or difficult to understand?

If so, please specify in the comment box below.

Section 3: Housing and physical activity

How to interpret your answers

😍: Great - 😃: Good - 😕: Room for improvement - 😟: Welfare concern -😣: Serious welfare concern

🔍: You need to know your parrot better. Take time to observe your parrot, either directly or by using a camera.

Please consult a behavioural consultant or veterinarian to review your assessment results and, if needed, develop an effective plan to improve your parrot's welfare.

Enclosure type

Where is your parrot's enclosure located?

If your parrot has multiple enclosures, please specify all locations

Enclosure: space where the parrot sleeps, eats, drinks, and stays when it can't be supervised

	Main enclosure (space where the parrot stays most of time)	Secondary enclosure (additional space used occasionally)	Not applicable
Living room			
Hallway			

Kitchen			
Bedroom			
Garage			
Basement			
Parrot lives indoor and does not have a dedicated enclosure			
Room exclusively dedicated to the parrot			
Outdoor enclosure			
Other			

Enclosure size

Which statement most accurately describes the size of your parrot's main enclosure (where it resides most of the daytime) and its ability to move around?

For information on species wingspan measurements and recommended minimum cage sizes, please consult the following link: https://naturalinspirationsparrotcages.com/p/wingspan-info

Choose one of the following answers

- 😃 - My parrot has ample space to move, climb, hop and fly
- 😕 - My parrot has at least 2 wingspan of space in all directions to move, climb, and hop
- 😞 - My parrot is unable to fully extend its wings in one or more directions, with limited space to move

Enclosure material

What type of material is your parrot's enclosure made from?

Check all that apply

- 😍 - Stainless steel
- 😃 - Powder-coated metal
- 😕 - Wrought iron
- 😞 -Plastic-coated wire, zinc, wood or acrylic
- 🔍 - I don't know
- Other:

Bars orientation

Please select the statement(s) that most accurately describe the bar orientation of your parrot's enclosure(s).

Check all that apply

- 😍 - The enclosure(s) has grid-patterned bars
- 😃 - The enclosure(s) has vertical bars
- 😟 - The enclosure(s) has horizontal bars
- 😞 -There are no bars to allow climbing (e.g. solid panels made of glass, acrylic)

Number of perches

How many perches are available in the parrot's enclosure(s)?

Choose one of the following answers

- 😍 - > 5
- 😃 - 3 – 5
- 😟 - 1 – 2
- 😞 - There are no perches

Perches' characteristics

Do you provide perches of...

	😃 - Yes	😟 - No
different sizes?		
different materials?		

Provision of opportunities that allow movement and climbing

Please indicate if your parrot receives the following types of enrichment that promote movement and climbing.

	Inside the enclosure			Outside the enclosure	

	- Yes	- No		- Yes	- No
Ropes					
Swings					
Ladders					
Boings					
Branches					

Safety of enrichment material

Have you checked whether the material of the enrichment provided (perches, ropes, branches etc.) is safe for parrots?

Choose one of the following answers

- Yes, I consulted my veterinarian or a behavioural consultant
- Yes, I researched myself online, in books, or magazines
- No

Presence of a retreating area/room to rest, sleep or withdraw

Does your parrot have unlimited access to an undisturbed area where it can rest, sleep or retreat from potential stressful situations (noise, presence of unfamiliar people, other animals etc.)?

Choose one of the following answers

- Yes
- No

Opportunity to spent time outside the enclosure

How often does your parrot spend time out of its enclosure ?

Choose one of the following answers

- Most or all of its time (e.g. only in the enclosure when sleeping or when there is no human supervision)
- Every day, for 3 hours or more
- Every day, for 3 hours or less
- Several times a week, but not every day
- Never or less than once a week on average

Hygiene: frequency of cleaning food and water bowls, foraging toys, enclosure

How often do you clean the...

	- Everyday or after every use	- Several times a week, but not every day	- Once a week or less
Enclosure (cage, room, aviary)?			
Food bowl(s)?			
Water bowl(s)?			
Foraging toys?			

Climate (temperature and humidity)

Do you regularly check the humidity and temperature to ensure they are appropriate for your parrot's species?

Choose one of the following answers

- Yes, both
- Only temperature
- Only humidity
- No

Air quality

How often do you refresh the air in the area where your parrot lives and spends most of its time?

Choose one of the following answers

- More than once per day / I use an air purifier / My parrot lives outdoor
- Once per day / I use an air purifier but is not constantly activated
- Several times a week, but not every day
- Once per week or less

Access to outdoor spaces

How often does your parrot spend time outdoor, if environmental circumstances permit (no risk of predation or mosquitoes' bites, appropriate temperature)?

Choose one of the following answers

- At least once a day
- At least once a week
- Less than once a week on average
- Never

Exposure to direct sunlight/UV light

How often do you expose your parrot to a UVA and UVB lamp or direct sunlight without a window in between?

Choose one of the following answers

- Everyday
- Weekly
- Monthly
- Never

Opportunity to spend time in high positions

Does your parrot have the opportunity to perch in high locations that are out of reach of people and other animals?

Choose one of the following answers

- Yes, both inside and outside of the enclosure
- Only inside the enclosure
- Only outside the enclosure
- No

Ability to fly

How would you describe your parrot's flight ability?

If you are unsure of your parrot's flying ability, please select the answer "I don't know." Avoid attempting to assess it, as it could lead to injury or stress for your parrot.

Choose one of the following answers

- My parrot flies in all directions and maintains height during flight
- My parrot flies in a downward trend and horizontally but cannot gain altitude
- My parrot attempts flying but is unable to do so and falls to the ground
- My parrot does not fly or does not attempt to fly
- I don't know

Wing trim (follow up question for ability to fly) - Answer options will be accompanied by illustrations

Has your parrot received a wing trim?

Wing trimming is often regarded as unethical and a welfare concern, as it restricts parrots from performing natural behaviours such as flying. As a result, it is prohibited by law in several countries.

Choose one of the following answers

- No
- My parrot has received a bilateral skinny wing trim
- My parrot has received a bilateral, transverse wing trim
- My parrot has received a bilateral wing trim which includes the primaries and secondaries
- My parrot has received a unilateral wing trim
- My parrot has been permanently deflighted through a surgical procedure (e.g., pinioning)

Level of activity

How much time does your parrot spend each day moving, climbing, and flying?

Choose one of the following answers

- 1 hour or less
- Between 1 and 2 hours
- Between 2 and 4 hours
- 4 or more hours
- I don't know

Opportunity to fly

How often does your parrot have the opportunity to fly in a safe space?

Choose one of the following answers

- Every day for 4 or more hours

- [illegible]
- 😃 - Every day for less than 4 hours
- 😟 - Several times a week, but not every day
- 😞 - Never or less than once a week on average

Was any part of this section unclear or difficult to understand?

If so, please specify in the comment box below.

Was any information in this section inaccurate or incorrect?

If so, please specify in the comment box below.

Section 4: Provision of enrichment and exploration

How to interpret your answers

😍: Great - 😃: Good - 🙂: Ok - 😕: Room for improvement - 😟: Welfare concern -😞: Serious welfare concern

🔍: You need to know your parrot better. Take time to observe your parrot, either directly or by using a camera.

Please consult a behavioural consultant or veterinarian to review your assessment results and, if needed, develop an effective plan to improve your parrot's welfare.

Provision of foraging and cognitive enrichment

How often do you provide the following types of enrichment?

	😍 - Every day	😃 - Weekly	😟 - Few times per month or less	😞 - Never
Certified chewable toys, cardboard or paper without ink, natural, not toxic and untreated cork and branches that can be safely chewed and destroyed	○	○	○	○
Puzzles and problem solving games 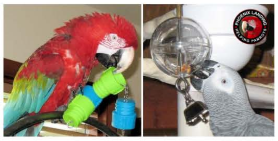 *Photos by Ann Brooks (Phoenix Landing Foundation)*	○	○	○	○
Multiple food stations	○	○	○	○
Scatter feeding (e.g. foraging mat, spreading food out in various locations) and foraging tray or box (food mixed with inedible items) 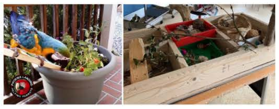 *Photos by Ann Brooks (Phoenix Landing Foundation)*	○	○	○	○
Larger chunks of food or whole food items (with or without skewers)	○	○	○	○
Non destructible puzzle feeders/foraging toys 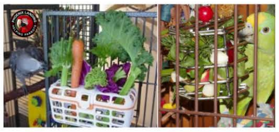 *Photos by Ann Brooks (Phoenix Landing Foundation)*	○	○	○	○
Destructible foraging toys 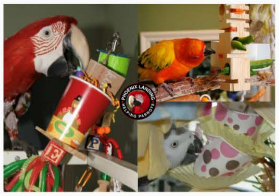 [illegible]	○	○	○	○

Photos by Ann Brooks (Phoenix Landing Foundation)				
Interactive toys that make sounds and/or move	○	○	○	○
Auditory enrichment (e.g. radio, music)	○	○	○	○
Visual enrichment (e.g. tv or tablets)	○	○	○	○

Interaction with enrichment (follow up question for Provision of foraging and cognitive enrichment)

How does your parrot interact with the enrichment provided?

Examples of interaction: manipulation with beak and/or feet (e.g., chewing, grasping, exploring) or reactions to sound and visual cues.

	😍 - Frequently and repeatedly uses with the enrichment, exploring and manipulating it extensively, showing continued interest until the item is destroyed (in case of destructible items) or food/contents is removed (if applicable)	😃 - Shows brief interest in the enrichment and ignores it at other times	☹️ - Does not use or interact with the enrichment at all, and completely ignores it	🔍 - I don't know
Certified chewable toys, carboard or paper without ink, natural, not toxic and untreated cork and branches that can be safely chewed and destroyed	○	○	○	○
Puzzles and problem solving games	○	○	○	○
Multiple food stations	○	○	○	○
Scatter feeding (e.g. foraging mat, spreading food out in various locations) and foraging tray or box (food mixed with inedible items)	○	○	○	○
Larger chunks of food or whole food items (with or without skewers)	○	○	○	○
Non destructible puzzle feeders/foraging toys	○	○	○	○
Destructible foraging toys	○	○	○	○
Interactive toys that make sounds and/or move	○	○	○	○
Auditory enrichment (e.g. radio, music)	○	○	○	○
Visual enrichment (e.g. tv or tablets)	○	○	○	○

Time spent foraging

How much time does your parrot spend daily foraging (i.e., searching for, procuring, and extracting food from enrichment that you provide)?

❗ Choose one of the following answers

○ 😒 - My parrot does not forage

○ ☹️ - Less than 1 hour

○ 🙂 - Between 1 and 2 hours

○ 😃 - Between 2 and 4 hours

○ 😍 - 4 hours or more

○ 🔍 - I don't know

Provision of foraging enrichment in relation to the daily food ration

How is foraging enrichment provided for your parrot?

Foraging enrichment includes toys, devices, or other opportunities that encourage natural behaviors such as searching, procuring, and extracting food.

❗ Choose one of the following answers

○ 😍 - For treats and more than half of the daily food ration

○ 😃 - For treats and about half of the daily food ration

○ 😕 - For treats and a small portion of of the daily food ration

○ ☹️ - Only for treats

Opportunities to select items based on preference (e.g., for colour, shape or type of material)

Do you select toys based on your parrot's preference for shape, size and colours?

❗ Choose one of the following answers

○ 😍 - Yes

○ 😕 - No

Enrichment replacement

How often do you replace toys/chewable items/ climbing enrichment by introducing new different one/s?

❗ Choose one of the following answers

○ 😍 - Every day

- 🤩 - Every day
- 😃 - Weekly
- 😟 - Few times per month or less
- 😞 - Never

Response to novel objects

How does your parrot generally react towards unfamiliar objects (e.g. new toys, new home decoration) in its proximity?

Please note that the behavioural response could be linked to your parrot's personality. A negative behavioural response may not change, but you can help prevent it by avoiding the situations that trigger it.

❗ Choose one of the following answers

- 🤩 - My parrot actively approaches the object touching, chewing and manipulating it; certain objects may still trigger freezing or withdrawing responses.
- 😃 - My parrot appears interested in the object, and cautiously approach it; certain objects (e.g. larger furniture or electronic household devices) may still trigger freezing or withdrawing responses.
- 😟 - My parrot withdraws or freezes to most objects; certain objects (e.g. larger toys, larger furniture or electronic household devices) may trigger more freezing or withdrawing responses.
- 😞 - My parrot shows one or more of these behaviours: excessive or incessant screaming, frantic movements, flying away, falling of the perch. This response happens with any type of unfamiliar object.

Alertness

What is your parrot's current alertness level?

❗ Choose one of the following answers

- 🤩 - My parrot is very active and curious. It eagerly explores new objects, interacts frequently with people and toys, and responds quickly to its surroundings
- 😃 - My parrot responds to sounds, movements, and interactions. It engages with toys, interacts with people, and explores its environment with moderate enthusiasm
- 😟 - My parrot reacts occasionally but needs encouragement to engage. It shows some interest in its surroundings and sometimes participates in activities
- 🚨 - My parrot is mostly inactive or lethargic, shows little interest in its surroundings and does not respond much to things happening around it

Was any part of this section unclear or difficult to understand?

If so, please specify in the comment box below.

Was any information in this section inaccurate or incorrect?

If so, please specify in the comment box below.

Section 5: Nutrition and maintenance behaviours

How to interpret the answers

🤩: Great - 😃: Good - 😕: Room for improvement - 😟: Welfare concern -😞: Serious welfare concern

🔍: You need to know your parrot better. Take time to observe your parrot, either directly or by using a camera.

Please consult a behavioural consultant or veterinarian to review your assessment results and, if needed, develop an effective plan to improve your parrot's welfare.

Food Selectivity

Does your parrot eat all types of food that you provide?
Observing the animal while it eats is preferable, but checking the food bowl can also provide valuable insights.

❗ Choose one of the following answers

- 😃 - Yes
- 😟 - No
- 🔍 - I don't know

Composition of the diet

Which of the following foods do you provide?

Please note that the appropriate amount of the foods listed depends greatly on your parrot's species, age, and individual needs. This table provides an overview of many different types of foods that can be included in a parrot's diet and may serve as a useful tool to discuss dietary choices with your veterinarian. Always consult a qualified avian vet to determine the most appropriate diet for your parrot.

🚩: Foods that should be offered only in limited amounts

⛔: Foods that should always be avoided

	Main component of the diet	Moderate to small quantities	As treat, training reward or vehicle for medications	Not provided
Pellet and/or other formulated food	◯	◯	◯	◯
Fresh vegetables	◯	◯	◯	◯
Fresh fruit	◯	◯	◯	◯
Sprouted seeds	◯	◯	◯	◯
Grains	◯	◯	◯	◯
Legumes	◯	◯	◯	◯
Tofu and/or other plant-based proteins	◯	◯	◯	◯
Seed mix	◯	◯	◯	◯
🚩- Nuts	◯	◯	◯	◯
Eggs	◯	◯	◯	◯
🚩 - Dairy products	◯	◯	◯	◯
⛔ - Other animal-based proteins	◯	◯	◯	◯
Nectar	◯	◯	◯	◯
⛔ - Processed food specifically designed for human consumption	◯	◯	◯	◯

Diet apropriateness

Have you checked whether the diet you provide is balanced and appropriate for your parrot's species?

❗ Choose one of the following answers

- ◯ 😃 - Yes, I consulted my veterinarian or a behavioural consultant
- ◯ 😕 - Yes, I researched by myself online, in books, or magazines
- ◯ 😞 - No

Availability of fresh and clean water

How often do you change your parrot's water?

❗ Choose one of the following answers

- ◯ 😍 - More than twice a day or as needed throughout the day
- ◯ 😃 - Twice a day
- ◯ 😕 - Once a day
- ◯ 😟 - Less than once per day

Daytime spent sleeping / resting

What daytime does your parrot sleep / rest?

❗ Check all that apply

- ☐ Morning
- ☐ Afternoon
- ☐ Evening
- ☐ Night
- ☐ I don't know

Changes in food and/or water consumption

Have you observed a change in appetite or water intake by your parrot in the last 2 weeks?
i.e. you observed these changes directly observing the parrot's behaviour or by checking the amount of food or water consumed.

❗ Choose one of the following answers

- ◯ 😃 - No
- ◯ 😞 - Yes
- ◯ 🔍 - I don't know

Changes of resting-sleeping patterns

Have you noticed any changes in the sleeping pattern of your parrot in the last two weeks?
e.g. sleeping more than usual or at times of the day when it typically does not sleep.

❗ Choose one of the following answers

- ◯ 😃 - No

😞 - Yes

🔍 - I don't know

Opportunity to bathe
Please indicate how often you provide the following bathing opportunities for your parrot

	Every day	Weekly	Few times per month or less	Never
Mist	◯	◯	◯	◯
Shower	◯	◯	◯	◯
Shallow dish or tray	◯	◯	◯	◯
Other	◯	◯	◯	◯

Interest in bathing

How does your parrot bathe when you provide the opportunity to do so?

❶ Choose one of the following answers

😍 - My parrot always bathes on its own

😃 - My parrot bathes on its own but sometimes needs encouragement to do so

😕 - My parrot always needs encouragement to bathe

😟 - My parrot doesn't bathe, and I don't try to change that

😞 - My parrot tries to avoid bathing, but I make sure it gets bathed anyway

🔍 - I don't know

Beak maintenance

Does your parrot engage in self-maintenance behaviors for its beak?

e.g. rubbing it against hard objects, grinding it by making a rhythmic motion with the upper and lower mandibles, or using bars or other objects to clean the inside of its beak

❶ Choose one of the following answers

😃 - Yes

😞 - No

🔍 - I don't know

Changes in preening activity

Have you noticed any changes in your parrot's self-preening behavior (such as using its beak to clean, arrange, or maintain its feathers) over the past month?

❶ Choose one of the following answers

😃 - No

😞 - Yes

🔍 - I don't know

Was any part of this section unclear or difficult to understand?

If so, please specify in the comment box below.

Was any information in this section inaccurate or incorrect?

If so, please specify in the comment box below.

Section 6: Social and reproductive behaviours

How to interpret your answers

😍: Great - 😃: Good - 😕: Room for improvement - 😟: Welfare concern -😞: Serious welfare concern

Please consult a behavioural consultant or veterinarian to review your assessment results and, if needed, develop an effective plan to improve your parrot's welfare.

Social housing (alone vs pair vs group)

Please select the option that best describes your parrot's social contact with other parrot.

Please note that parrots are highly social animals, and living in group is important for their welfare. However, introducing new individuals should always be carefully evaluated with an expert to ensure it is suitable and safe for all parrots involved.

Choose one of the following answers

- My parrot is housed together with two or more other parrots and can continuously interact with them
- My parrot is housed together with one parrot and they can continuously interact
- My parrot can physically interact with one or more parrots at certain times of the day (or week), but is not continuously housed together with other parrots
- My parrot is housed individually, but is able to see other parrots and can vocally interact with them
- My parrot lives alone and does not have any type of contact with other parrots

Social behaviours

How frequently do these social interactions occur?

	- On all or most encounters	- On some encounters	- Rarely to never
Staying right beside another parrot			
Feeding alongside another parrot			
Preening another parrot			
Being preened by another parrot			
Vocal interaction with another parrot (contact calls)			

	- Rarely to never	- On some encounters	- On all or most encounters
Mating with another parrot			
Regurgitating food for other parrot			
Having food regurgitated by another parrot			
Stalking (i.e. following or watching insistently) another parrot			
Stalked (i.e. followed or watched insistently) by another parrot			
Attacks (biting, lunging) another parrot			
Being attacked (bitten or lunged at) by another parrot			

Reproductive behaviours

How frequently do you observe these behaviors?

	- Never	- Few times per month or less	- Weekly	- Every day
Rubbing its cloaca (vent area) against objects such as perches, cage bars, toys				
Actively seeking or spending time in dark, enclosed, or secluded spaces such us underneath furniture, inside drawers or cabinets, behind cushions, or in boxes or clothing.				
Lunging, biting, vocalizing aggressively, or chasing any-one who approaches or gets too close to a specific area (e.g. cage or nest)				
Egg laying				
Regurgitating food and directing it towards its own body parts (e.g. its foot)				
Regurgitating food on a special toy or item in the cage				

Was any part of this section unclear or difficult to understand?

If so, please specify in the comment box below.

Was any information in this section inaccurate or incorrect?

If so, please specify in the comment box below.

Section 7: Parrot-Human interactions

How to interpret your answers

😍: Great - 😃: Good - 🙂: Ok - 😕: Room for improvement - 😟: Welfare concern -😣: Serious welfare concern

🔍: You need to know your parrot better. Take time to observe your parrot, either directly or by using a camera.

Please consult a behavioural consultant or veterinarian to review your assessment results and, if needed, develop an effective plan to improve your parrot's welfare.

Types of interaction with humans (table 1)

How often do you allow your parrot to engage in the following behaviors?

	😃 - Never	😕 - Few times per month or less	😟 - Weekly	😣 - Every day
Sitting on shoulder, lap, or another part of the body				
Gently nibbling or grooming hair, beard, eyelashes, or skin with its beak (preening)				
Crawling under clothes or blankets				

Time spent in presence of humans

How many hours per day is your parrot surrounded by you and/or familiar people (e.g. partner, family member)?

❗ Choose one of the following answers

- ( ) 😣 - <1h
- ( ) 😕 - It varies greatly per day
- ( ) 🙂 - 2h – 4h
- ( ) 😃 - 4h - 8h
- ( ) 😍 - > 8h
- ( ) 😟 - All time, including night

Types of interaction with humans (table 2)

How often do the following interactions take place with the parrot?

	😍 - Every day	😃 - Weekly	😕 - Few times per month or less	😟 - Never
Petting the parrot's head, cheeks and/or neck				
Gently responding to the parrot's vocalizations				
Talking to the parrot				
Playing music for my parrot				
Taking the parrot along when going out				
Training the parrot				

	😃 - Never	😕 - Few times per month or less	😟 - Weekly	😣 - Every day
Petting the parrot under the wings				
Petting the parrot's chest				
Petting the parrot's back and tail				
Holding and shaking the parrot's beak while playing				
Yelling at the parrot				
Kissing the parrot				
Providing food by mouth or allowing the parrot to eat from the mouth				
Pressing on parrot's chest to encourage it to step up onto hands, arms, or an offered perch				
Holding the parrot's body in the hands (with or without gloves or a towel)				
Grabbing the parrot with a net or a towel				

Parrot response to training (follow up question if the owner indicates they train their parrot)

How does your parrot respond during training session?

❗ Choose one of the following answers

- ( ) 😍 - Very focused and responds quickly
- ( ) 😃 - Mostly focused, but gets distracted sometimes
- ( ) 🙂 - Responds sometimes, but often gets distracted
- ( ) 😕 - Rarely focused, responds only occasionally
- ( ) 😟 - Does not respond or shows no interest in training

Behaviours directed towards humans

How frequently does your parrot display the behavior below towards humans?

	- Every day	- Weekly	- Few times per month or less	- Never
Offering the head/neck to be petted	○	○	○	○
Contact calls/vocalizations	○	○	○	○
Crouching with the head down	○	○	○	○
Bowing and bobbing the body	○	○	○	○
Voluntarily steps up onto your hand, arm, or an offered perch without encouragement	○	○	○	○

	- Never	- Monthly	- Weekly	- Everyday
Begging for food (raise its wings, flutter them and bob its head up, and down in a rhythmic pattern)	○	○	○	○
Regurgitating food	○	○	○	○
Masturbation (rubbing the cloaca against humans)	○	○	○	○
Approaching a person and biting	○	○	○	○

Human selectivity (follow up question for Behaviours directed towards humans)

To how many people does your parrot display each of the behaviour(s) selected above?

	To everyone	To few people	Only to me
Offering the head/neck to be petted	○	○	○
Contact calls/vocalizations	○	○	○
Crouching with the head down	○	○	○
Bowing and bobbing the body	○	○	○
Voluntarily steps up onto your hand, arm, or an offered perch without encouragement	○	○	○
Begging for food (raise its wings, flutter them and bob its head up, and down in a rhythmic pattern)	○	○	○
Regurgitating food	○	○	○
Masturbation (rubbing the cloaca against humans)	○	○	○
Moving toward a person and biting	○	○	○

Response upon contact with caregiver

How does your parrot respond to you as the caregiver?

	- Every or most times	- Sometimes	- Rarely to never	- I don't know
My parrot accepts my presence near the enclosure or stand	○	○	○	○
My parrot accepts physical contact initiated by me	○	○	○	○
My parrot initiates physical contact itself by actively approaching me	○	○	○	○

	- Rarely to never	- Sometimes	- Every or most times	- I don't know
My parrot stays completely still and does not react when I am approaching it	○	○	○	○
My parrot tries to lunge, attempt to bite or chase me when I am approaching it	○	○	○	○
My parrot tries to escape and avoid contact when I am approaching it	○	○	○	○

Response upon contact with familiar person

How does your parrot respond to a familiar person (e.g. partner, family member, friend)?

	- Every or most times	- Sometimes	- Rarely to never	- I don't know
My parrot accepts their presence near the enclosure or stand	○	○	○	○
My parrot accepts physical contact initiated by them	○	○	○	○
My parrot initiates physical contact itself by actively approaching them	○	○	○	○

	- Rarely to never	- Sometimes	- Every or most times	- I don't know
My parrot stays completely still and does not react when they are approaching it	○	○	○	○
My parrot tries to lunge, attempt to bite or chase them when they are approaching it	○	○	○	○
My parrot tries to escape and avoid contact when they are approaching it	○	○	○	○

Comfort behaviour around humans

Does your parrot eat, drink, rest and/or clean its feathers...

Please note that the behavioural response could be linked to your parrot's personality. A negative behavioural response may not change, but you can help prevent it by avoiding the situations that trigger it.

	😀 - Yes	😞 - No	🔍 - I don't know
In your presence?	○	○	○
in the presence of all household members?	○	○	○
in presence of people that come by regurarly?	○	○	○
in presence of people that come by infrequently?	○	○	○

Does your parrot spend most of its time in high locations out of human reach...

Please note that the behavioural response could be linked to your parrot's personality. A negative behavioural response may not change, but you can help prevent it by avoiding the situations that trigger it.

	😀 - No	😞 - Yes	🔍 - I don't know
In your presence?	○	○	○
in the presence of all household members?	○	○	○
in presence of people that come by regurarly?	○	○	○
in presence of people that come by infrequently?	○	○	○

Was any part of this section unclear or difficult to understand?

If so, please specify in the comment box below.

Was any information in this section inaccurate or incorrect?

If so, please specify in the comment box below.

Section 8: Abnormal and fear-related behaviours

How to interpret your answers

😍: Great - 😀: Good - 😕: Room for improvement - 😟: Welfare concern - 😞: Serious welfare concern

🔍: You need to know your parrot better. Take time to observe your parrot, either directly or by using a camera.

Please consult a behavioural consultant or veterinarian to review your assessment results and, if needed, develop an effective plan to improve your parrot's welfare.

Expression of avoidance and escape behaviours

When does your parrot display any of the following behaviors?

Please note that the behavioural response could be linked to your parrot's personality. A negative behavioural response may not change, but you can help prevent it by avoiding the situations that trigger it.

❓ The answer options range from the worst conditions at the top to the best conditions at the bottom

	Tremors or shivering, freezing, hiding, withdrawing	Attempting to escape by flying or moving away, possibly falling off the perch, screeching/high-pitched screams
😍 - Never	○	○
😀 - Mostly in response to specific or uncommon situations (e.g. visit to the vet, sudden loud noise)	○	○
😕 - Mostly when exposed to a certain situation, outside of its daily environment (e.g. outdoor activity, visit of a new place)	○	○
😟 - Predominantly when exposed to changes in its daily environment (e.g. presence or approach by new, unfamiliar people/guests or animals, change of furniture, new house decorations, provision of new toys)	○	○
😞 - Most of the time, even in its daily environment	○	○
🔍 - I don't know	○	○

Disruptive vocalization or screams

How often does your parrot produce disruptive, loud vocalizations or screams?

❗ Choose one of the following answers

○ 😍 - Never

○ 😀 - Rarely and mostly limited to specific moments of the day (e.g. morning and evening) or in response to specific and uncommon situations (e.g. sudden loud noise)

- 😕 - Occasionally and mostly when exposed to specific contexts (e.g. when left alone, presence of unfamiliar people/guests)
- 😟 - Frequently, and incessant screaming can occur but mostly in specific contexts (e.g. when left alone, presence of unfamiliar people/guests)
- 😑 - For the majority of the day, and sometimes incessantly for hours with no apparent reason or cause

Abnormal, sham and excessive behaviours

Does your parrot exhibit any of the following behaviors?

Please note that interpreting abnormal behaviours can be challenging. If you observe any of the behaviours in the table, seek expert guidance to evaluate whether they reflect a welfare issue.

	😑 - Yes	🙂 - No	🔍 - I am not sure
Pacing: repetitive walking back and forth along a fixed path	○	○	○
Route tracing: repeatedly follow the same path or pattern within its enclosure, such as moving along a specific perch, climbing the same section of the cage, or flying in a predictable loop	○	○	○
Swaying and rocking: repeatedly shift its body side to side or back and forth in a rhythmic motion	○	○	○
Tongue rolling and flicking: repetitive quick movement of the tongue or rolling or flicking of the tongue inside or outside the beak	○	○	○
Beak clacking: rapid, repetitive, excessive or compulsive clicking or clacking of the beak	○	○	○
Beak rubbing: repeatedly rubbing the beak against surfaces such as perches or bars	○	○	○
Repetitive licking: repeated licking of surfaces such as cage bars, perches, or walls	○	○	○
	😑 - Yes	🙂 - No	🔍 - I am not sure
Spot pecking: repetitively peck at a specific spot, such as a cage bar, perch, wall, or even an imaginary point	○	○	○
Sham bathing: mimic bathing motions without the presence of water or an appropriate bathing substrate	○	○	○
Sham chewing and chewing not chewable items: mimics chewing motions or engages with non-chewable objects (e.g., metal bars or plastic) or surfaces	○	○	○
Toe-nail biting: repeatedly bite or nibble at their own toes or nails	○	○	○
Feeding objects (e.g. mirrors, toys)	○	○	○

Was any part of this section unclear or difficult to understand?

If so, please specify in the comment box below.

Was any information in this section inaccurate or incorrect?

If so, please specify in the comment box below.

Feedback on the Welfare Assessment Tool

Please indicate your area of expertise

Check all that apply

- ☐ Veterinarian
- ☐ Behavioural consultant
- ☐ Other: ______

Which country are you currently living in?

How did you find the length of the assessment?

If you have any additional thoughts or suggestions, please share them in the comment box below.

Choose one of the following answers

- It was too long and felt tiring or overwhelming to complete.
- A bit lengthy, but definitely useful and worth completing.
- The length was appropriate and well-balanced.
- It felt a bit short, I expected more questions.

Please enter your comment here:

Were there any questions you felt were missing from the tool?

If yes, please specify them in the comment box below.

[For professionals who work with parrot owners]

Do you think this tool could facilitate your professional interactions with parrot owners?

If you have any additional thoughts or suggestions, please share them in the comment box below.

Choose one of the following answers

- Yes, definitely, it would be a valuable resource to support and guide owners effectively.
- Yes, to some extent, it could help in certain cases, depending on the owner or situation.
- Not sure, I would need to see how it works in practice before deciding.
- Probably not, I don't see it making a significant difference in my work.

Please enter your comment here:

Submit
