# Supplementary material for: PsittaWel: A welfare assessment tool for companion parrots
Source: Anim Welf. 2026 May 14;35:e34. doi: 10.1017/awf.2026.10089 (PMC13175777; doi:10.1017/awf.2026.10089)
Supplement: Piseddu et al. supplementary material [file S096272862610089Xsup001.zip › Supplementary materials.pdf]

Supplementary materials

PsittaWel: A welfare assessment tool for companion parrots

Andrea Piseddu[1*], Yvonne R. A. van Zeeland[2], Lauren M. Hemsworth[3] and Jean-Loup Rault[1]

[1]Animal Welfare Science Unit, University of Veterinary Medicine Vienna, Veterinaerplatz 1, 1210 Vienna, Austria
[2]Division of Zoological Medicine, Department of Clinical Sciences, Faculty of Veterinary Medicine, Utrecht University, Yalelaan 108, 3584 CM Utrecht, The Netherlands
[3]Animal Welfare Science Centre, Faculty of Science, University of Melbourne, Parkville, Victoria, Australia
*Author for correspondance : andrea.piseddu@vetmeduni.ac.at

Methods

Selection of welfare indicators

Table S1. List of the animal- and environment-based indicators initially selected for their importance and rephrased into questions and inclusion in the welfare assessment tool.

**Animal-based indicator**	
**Category**	**Indicators**
**Maladaptive and fear-related behaviours**	Feather destructive behaviour (chewing, biting, fraying, plucking)
	Expression of escape and fear-related behaviours (combined with tremors, hiding and freezing)
	Excessive vocalization/screaming
**Exploratory behaviours**	Interaction with enrichment
	Response to novel objects
**Locomotor behaviours**	Inability to fly (physical restrictions due to cage size or trimming of feathers)
**Human directed behaviours**	Response upon contact with caregiver (combined with withdrawal from interaction)
	Response upon contact with familiar person (combined with withdrawal from interaction)
**Body Measurement**	Mentation/alertness
	Condition of body feathers (contour/down feathers) (combined with condition of flight feathers)
	Appearance of droppings (e.g., colour, consistency) (combined with number of droppings)
**Maintenance behaviours**	Daily Food Intake
	Amount of time spent sleeping
**Social behaviours**	Time spent in vicinity of other parrots
**Environment-based indicators**	
**Category**	**Indicators**
**Provision of enrichment**	Opportunities to do physical exercise (flying, climbing, etc.)
	Provision of foraging enrichment
	Provision of cognitive enrichment
	Variety of enrichment provided
**Housing**	Time spent out of the cage
	Cage characteristics (e.g., dimension, material, bars orientation)
	Access to outdoor spaces
**Nutrition**	Composition of the diet (quantity of fat, cholesterol, fibre, etc.)
**Parrot-Human Interactions**	Rearing History
**Social needs**	Social housing (alone vs pair vs group) (combined with level of social contacts: only vocal, visual and vocal, physical)

Table S2. List of the animal- and environment-based indicators presented to the panel of experts for potential inclusion in the welfare assessment tool. Indicators marked with an asterisk (*) were grouped together and, if included, intended to be rephrased into a single question.

Category	Animal-based indicator
**Maladaptive and fear-related behaviours**	Whole body stereotypies (head bobbing, rocking)
	Locomotor stereotypies (route-tracing, pacing)
	Sham behaviours (e.g., sham chewing, sham flying, sham bathing)
	Excessive chewing (e.g., wires)
	Toe/nail biting
	Excessive masturbation
	Spot pecking
**Exploratory behaviours**	Response in unfamiliar environments
	Time spent foraging
	Response to novel food items
	Response to electronic devices
**Locomotor behaviours**	*Level of activity (time spent inactive vs active) + walking + climbing + flying
	Swinging
	Time spent in high positions
**Human directed behaviours**	Food-related interaction (e.g., begging for food, acceptance of food from the hand, regurgitation of food to humans)
	Sexual related behaviours (e.g., panting, receptive posture)
	Contact seeking behaviours in absence of humans (e.g., vocalization, flapping wings)
	Ruffling of feathers (e.g., nape, crown, beard) during human interaction
	Initiation of contact with human being
	Response upon contact with unfamiliar person
	Aggression towards humans linked to a specific location or perimeter
	Aggressive towards humans
	Abnormal behaviours in presence of humans
**Body measurements**	Respiration/breathing changes (e.g., frequency, depth)
	Posture (e.g., fluffed appearance, weight bearing)
	Body weight
	Pectoral muscle condition score
	Eye appearance (e.g., half-open, discharge)
	Gait changes (incl. flight)
	Condition of flight feathers (wing and tail)
	Length and frequency of moulting
	Prolapses
	Respiratory effort when disturbed
	Respiratory effort at rest
	Beak appearance (e.g., length, shape, position)
	Cere/nare appearance (e.g., colour, shape, size)
**Maintenance behaviours**	Preening activity (incl. time of day and frequency, duration)
	Daily water consumption
	Time of day (morning, afternoon, evening) spent sleeping/resting
	Interest in bathing

	Beak maintenance
**Reproductive behaviours**	Physical proximity between mates
	Masturbation
	Searching for nesting areas
	Territoriality during breeding season
	Copulation
	Nest defense
	Courtship feeding
	Mate allopreening
**Social behaviours**	Aggressive behaviour toward non-mates (e.g., chasing, biting, lunging)
	Allopreening
	Displacement behaviours during social interactions
	Play behaviour towards cage-mates
	Aggressive behaviour toward mates (e.g., chasing, biting, lunging)
	Aggressive behaviour toward mates chicks
	Frequency and duration of social interaction
**Environment-based indicators**	
**Category**	**Indicators**
**Enrichment**	Amount of enrichment provided
	Area of the room/cage where enrichment is placed
	Opportunities to select items based on preference (e.g., for colour, shape or type of material)
	Provision of auditory enrichment
	Provision of chewable items
	Provision of climbing toys, swings and ladders
	Provision of visual enrichment
	Rotation of enrichment
	Size of enrichment in relation to parrot size
**Housing**	Air quality (e.g. presence of air purifier, exposure to fresh air)
	Artificial light characteristics (e.g. type, intensity)
	Environmental humidity
	Environmental temperature
	Exposure to artificial light at night
	Exposure to direct sunlight/UV light
	Exposure to Noise
	Frequency of cage cleaning
	Perches’ characteristics (e.g., diameter, material)
	Position and height of the perches (e.g., in relation to feeders or human-eye level)
	Position of the cage in the room
	Presence of a nesting area
	Presence of a platform to stand on
	Presence of a retreating area/room to rest, sleep or withdraw
	Room where the cage is positioned (kitchen, living room, bedroom, etc.)
	Size of the room where the cage is positioned
	Size/number of windows in the room
**Nutrition**	Availability of clean fresh water
	Balance between provision of fresh and dried food

	Consumption of human food
	Frequency of cleaning the food bowls
	Frequency of fresh food provision
	Frequency with which food is provided
	Location and number of feeding areas
	Manner/way in which food is offered to the bird (presented in a bowl, via enrichment, etc.)
	Pellet size
	Provision of supplements (multivitamin, calcium, essential fatty acids, etc.)
	Time of day (morning, afternoon, evening) at which food is provided
	Variety of food items provided
	Way in which food is stored
**Parrot-Human Interactions**	Frequency/duration of manual restraint
	Human produces loud noises and/or sudden movements
	Number of people in the household
	Number of people in the household that regularly interact with the parrot
	Time spent on interaction with human
	Time spent without presence of a human
	Type of interaction with human (training, mouth to beak feeding, etc.)
**Social needs**	Frequency/duration of social separation events
	Opportunities for pair bonding (i.e., living with/without a mate)
	Partner/cage mate choice (free vs imposed)
	Type of social companionship (same vs different in terms of species, size, sex, origin, etc.)

Table S3. List of all online surveys and focus group meetings with the experts, including content of each survey, dates and topics discussed in each focus group meeting.

**Online surveys**		
**Survey number**	**Content of the survey**	**Number of experts that participated**
1	Refinement of questions proposed by the investigators, selection of additional welfare indicators to include in the assessment tool and corresponding rephrasing into question, identification of important missing information	7
2	Review of the first draft of the welfare assessment tool, identification of important missing information, re-organization of the indicators across sections	7
3	Review of the refined welfare assessment tool	6
4	Review of the refined welfare assessment tool	2
**Discussions on the first version of welfare assessment tool**		
**Dates of the meetings**	**Topics discussed (questions' refinement, inclusion, exclusion)**	**Attending experts (n)**
21.02.2025	Section re-organization and renaming, physical health and body measurements, parrot-human interactions, human directed behaviours	4
24.02.2025	Nutrition, maintenance behaviours, housing, physical activity	5
17.03.2025	Social needs, social and reproductive behaviours, enrichment, exploratory behaviours, maladaptive and fear-related behaviours	3
**Discussion on the first and second refinements of the welfare assessment tool**		
**Dates of the meetings**	**Topics discussed for final refinement**	**Attending experts (n)**
07.04.2025	Physical health and body measurements, housing and locomotor behaviours, enrichment, exploratory behaviours, nutrition, maintenance behaviours	3
12.05.2025	Inclusion of a timeframe for observation of behaviours, physical health, exploratory behaviours, parrot-human interactions	2

External experts and caregiver survey

Table S4. Questions and corresponding answer options included in the online surveys for external experts that did not participate in the focus group meetings and for parrot caregivers. The final two columns indicate whether each question was included (Yes or No) in the survey for experts, caregivers, or both. Questions marked with an asterisk were included at the end of each of the eight sections of the welfare assessment tool.

Question	Answer options	Experts	Caregivers
Was any part of this section unclear or difficult to understand?*	Comment box	Yes	Yes
Was there any behaviour or type of information that you found difficult to observe or collect in order to answer the questions in this section?*	Comment box	No	Yes
Was any information in this section inaccurate or incorrect?*	Comment box	Yes	No
How did you find the length of the assessment?	-It was too long and felt tiring or overwhelming to complete.<br>-A bit lengthy, but definitely useful and worth completing.<br>-The length was appropriate and well-balanced.<br>-It felt a bit short, I expected more questions.	Yes	Yes
Were there any questions you felt were missing from the tool?	Comment box	Yes	Yes
Do you think you would use this tool regularly to monitor your parrot's welfare?	-Yes, definitely, it would be a valuable resource to keep track of my parrot's welfare.<br>-Yes, to some extent, I might use it occasionally, depending on the situation.<br>-Maybe, I would need more time or repeated use to decide.<br>-Probably not, I don't think I would find it useful in my daily routine.	No	Yes
Do you think this tool could facilitate your professional interactions with parrot caregivers?	-Yes, definitely, it would be a valuable resource to support and guide caregivers effectively.<br>-Yes, to some extent, it could help in certain cases, depending on the caregiver or situation.<br>-Not sure, I would need to see how it works in practice before deciding.<br>-Probably not, I don't see it making a significant difference in my work.	Yes	No

Results

Table S5. List of all genera and species of companion parrots whose welfare was assessed by their caregivers in the online survey, with corresponding number of subjects per species.

Genera	Species	Number of subjects
Agapornis	*Agapornis roseicollis*	1
	Undefined *Agapornis* species	2
Amazona	*Amazona aestiva*	1
	Amazona ochrocephala	1
	Amazona oratrix	1
	Hybrid: *Amazona aestiva* x *Amazona oratrix*	1
	Undefined *Amazona* hybrid	1
Ara	*Ara ararauna*	2
	Ara glaucogularis	1
	Ara macao	1
	Ara maracana	1
Aratinga	*Aratinga jandaya*	2
	Aratinga weddellii	1
Cacatua	*Cacatua alba*	1
	Cacatua goffiniana	3
Cyanoramphus	*Cyanoramphus novaezelandiae*	2
Eclectus	*Eclectus roratus*	5
Eolophus	*Eolophus roseicapilla*	1
Melopsittacus	*Melopsittacus undulatus*	1
Myiopsitta	*Myiopsitta monachus*	3
Nymphicus	*Nymphicus hollandicus*	9
Pionites	Undefined *Pionites* species	1
Pionus	*Pionus chalcopterus*	1
	Undefined *Pionus* species	1
Poicephalus	*Poicephalus meyeri*	1
Polytelis	*Polytelis alexandrae*	2
	Polytelis anthopeplus	2
Psittacula	*Psittacula eupatria*	1
Psittacus	*Psittacus erithacus*	13
	Psittacus timneh	1
Pyrrhura	*Pyrrhura molinae*	1
	Pyrrhura rupicola	1
	Undefined *Pyrrhura* species	1

Table S6. List of all indicators included in each section of the welfare assessment tool, along with any additional indicators they were combined with, the type of question they were rephrased into, and their source, whether derived from the previous study, suggested by expert panel during the focus group meetings, or both. Indicators retained from the original list of those initially considered essential for inclusion are highlighted in bold.

Section	Indicator	Type	Additional indicators combined in the same question	Type of question	Source
General information	**Rearing History**	Environment-based	-	Multiple choice (single answer)	(Piseddu et al. 2025)
	Frequency of health general check-ups	Environment-based	-	Multiple choice (single answer)	Suggested by expert panel
	Daily time dedicated to observe parrot behaviour	Environment-based	-	Multiple choice (single answer)	Suggested by expert panel
	Veterinarian's avian specialization	Environment-based	-	Yes/No question	Suggested by expert panel
Physical health	**Condition of body and flight feather**	Animal-based	-	Multiple choice (single answer)	(Piseddu et al. 2025)
	Condition of head plumage	Animal-based		Multiple choice (single answer)	Suggested by expert panel
	**Number and appearance of droppings**	Animal-based	-	Multiple choice (single answer)	(Piseddu et al. 2025)
	Pectoral muscle condition score	Animal-based	-	Multiple choice (single answer)	(Piseddu et al. 2025)
	Presence of diagnosed disease	Environment-based	-	Yes/No question	Suggested by expert panel
	Signs of illness (see the welfare assessment tool for the list)	Animal-based	-	Table - Yes/No question	(Piseddu et al. 2025) + Suggested by expert panel
	Treatment for diagnosed diseases	Environment-based	-	Multiple choice (multiple answers)	Suggested by expert panel
Housing and physical activity	Enclosure position	Environment-based	-	Table-Multiple choice (single answers)	(Piseddu et al. 2025) + Suggested by expert panel
	**Enclosure size**	Environment-based	-	Multiple choice (single answer)	(Piseddu et al. 2025)
	**Enclosure material**	Environment-based	-	Multiple choice (multiple answers)	(Piseddu et al. 2025)

	**Enclosure bars orientation**	Environment-based	-	Multiple choice (multiple answers)	(Piseddu et al. 2025)
	Number of perches	Environment-based	-	Multiple choice (single answer)	Suggested by expert panel
	Perches' characteristics (diameter, material)	Environment-based	-	Multiple choice (single answer)	(Piseddu et al. 2025)
	**Provision of opportunities that allow movement and climbing (swings, ladders, etc.)**	Environment-based	-	Table - Multiple choice (single answer)	(Piseddu et al. 2025)
	Safety of enrichment's material	Environment-based	-	Multiple choice (single answer)	Suggested by expert panel
	Presence of a retreating area/room to rest, sleep or withdraw	Environment-based	-	Yes/No question	(Piseddu et al. 2025)
	**Opportunity to spent time outside the enclosure**	Environment-based	-	Multiple choice (single answer)	(Piseddu et al. 2025)
	Safety of indoor spaces	Environment-based		Multiple choice (single answer)	Suggested by expert panel
	Frequency of cage, bowls, toys cleaning	Environment-based	-	Table - Multiple choice (single answer)	Previous study + Suggested by expert panel
	Environmental temperature and humidity	Environment-based	-	Multiple choice (single answer)	(Piseddu et al. 2025)
	Air quality (e.g. presence of air purifier, exposure to fresh air)	Environment-based	-	Multiple choice (single answer)	(Piseddu et al. 2025)
	**Access to outdoor spaces**	Environment-based	-	Multiple choice (single answer)	(Piseddu et al. 2025)
	Safety of outdoor spaces	Environment-based		Multiple choice (single answer)	Suggested by expert panel
	Exposure to direct sunlight/UV light	Environment-based	-	Multiple choice (single answer)	(Piseddu et al. 2025)
	Opportunity to spent time in high positions	Environment-based	-	Multiple choice (single answer)	Suggested by expert panel
	**Ability to fly**	Animal-based	-	Multiple choice (single answer)	(Piseddu et al. 2025)

	Wing trim	Environment-based	-	Multiple choice (single answer)	Suggested by expert panel
	Opportunity to fly	Environment-based	-	Multiple choice (single answer)	Suggested by expert panel
	Safety of the space where the parrot is allowed to fly	Environment-based	-	Table- Multiple choice (single answer)	Suggested by expert panel
	Level of activity (time spent inactive vs active)	Animal-based	Walking, climbing, flying	Multiple choice (single answer)	(Piseddu et al. 2025)
Provision of enrichment and exploration	**Provision of foraging and cognitive enrichment** (see the assessment tool for the complete list of enrichment devices)	Environment-based	**Variety of enrichment provided**, provision of chewable devices	Table - Multiple choice (single answer)	(Piseddu et al. 2025)
	**Interaction with enrichment**	Animal-based	-	Table - Multiple choice (single answer)	(Piseddu et al. 2025)
	Time spent foraging	Animal-based	-	Multiple choice (single answer)	(Piseddu et al. 2025)
	Provision of enrichment in relation to the daily food ration	Environment-based	-	Multiple choice (single answer)	(Piseddu et al. 2025)
	Opportunities to select items based on preference (e.g., for colour, shape or type of material)	Environment-based	-	Yes/No question	(Piseddu et al. 2025)
	Enrichment replacement	Environment-based	-	Multiple choice (single answer)	(Piseddu et al. 2025)
	**Response to novel objects**	Animal-based	Response to electronic devices	Multiple choice (single answer)	(Piseddu et al. 2025)
	**Alertness**	Animal-based	-	Multiple choice (single answer)	(Piseddu et al. 2025)
Nutrition and maintenance behaviours	**Composition of the diet** (see the assessment tool for the list of food items included)	Environment-based	Frequency of fresh food provision, balance between provision of fresh and dried food, consumption of human food.	Table - Multiple choice (single answers)	(Piseddu et al. 2025) + Suggested by expert panel
	Food selectivity	Animal-based	-	Yes/No question	Suggested by expert panel
	Diet appropriateness	Environment-based	-	Multiple choice (single answer)	Suggested by expert panel
	Availability of clean fresh water	Environment-based	-	Multiple choice	(Piseddu et al. 2025)

				(single answer)	
	Daytime (morning, afternoon, evening) spent sleeping/resting	Animal-based	-	Multiple choice (multiple answers)	(Piseddu et al. 2025)
	Changes in resting/sleeping patterns	Animal-based	-	Yes/No question	Suggested by expert panel
	Changes in food and water consumptions	Animal-based	-	Yes/No question	Suggested by expert panel
	Opportunity to bathe	Environment-based	-	Table - Multiple choice (single answer)	Suggested by expert panel
	Interest in bathing	Animal-based	-	Multiple choice (single answer)	(Piseddu et al. 2025)
	Beak maintenance	Animal-based	-	Yes/No question	(Piseddu et al. 2025)
	Changes in preening activity	Animal-based	-	Yes/No question	(Piseddu et al. 2025)
Social and reproductive behaviours	**Social housing (alone vs pair vs group)**	Environment-based	Level of social contacts (only vocal, visual and vocal, physical)	Multiple choice (single answer)	(Piseddu et al. 2025)
	**Social behaviours** (see the assessment tool for the list of behaviours)	Animal-based	-	Table - Multiple choice (single answer)	(Piseddu et al. 2025) + Suggested by expert panel
	Reproductive behaviours (see the assessment tool for the list of behaviours)	Animal-based	-	Table - Multiple choice (single answer)	(Piseddu et al. 2025) + Suggested by expert panel
Parrot-human interactions	Time spent in presence of a human	Environment-based	-	Multiple choice (single answer)	(Piseddu et al. 2025)
	Behaviours allowed by humans	Environment-based		Table - Multiple choice (single answer)	Suggested by expert panel
	**Types of interaction with human** (training, mouth to beak feeding, etc.) (See the assessment tool for the list of interactions)	Environment-based	-	Table - Multiple choice (single answer)	(Piseddu et al. 2025) + Suggested by expert panel
	Parrot response to training	Animal-based	-	Multiple choice (single answer)	Suggested by expert panel
	Behaviours directed towards humans (see the assessment tool for the list of behaviours)	Animal-based	-	Table - Multiple choice (single answer)	(Piseddu et al. 2025) + Suggested by expert panel
	Human selectivity	Animal-based	-	Table - Multiple choice (single answer)	Suggested by expert panel

	**Response upon contact with caregiver** (see the assessment tool for the list of behaviours)	Animal-based	Initiation of contact with human being, withdrawal from interaction, freezing	Table - Multiple choice (single answer)	(Piseddu et al. 2025)
	**Response upon contact with familiar person** (see the assessment tool for the list of behaviours)	Animal-based	Initiation of contact with human being, withdrawal from interaction, freezing	Table - Multiple choice (single answer)	(Piseddu et al. 2025)
	Comfort behaviour around humans (maintenance behaviours)	Animal-based		Table - Multiple choice (single answer)	(Piseddu et al. 2025) + Suggested by expert panel
	Comfort behaviour around humans (time spent within reach)	Animal-based	Time spent in high positions	Table - Multiple choice (single answer)	(Piseddu et al. 2025) + Suggested by expert panel
Maladaptive and fear-related behaviours	**Expression of avoidance or escape behaviours** (see the assessment tool for the list of behaviours)	Animal-based	-	Table - Multiple choice (single answer)	(Piseddu et al. 2025)
	**Disruptive vocalizations or screams**	Animal-based	-	Multiple choice (single answer)	(Piseddu et al. 2025)
	Maladaptive, sham and excessive behaviours (see the assessment tool for the list of behaviours)	Animal-based		Yes/No table	(Piseddu et al. 2025) + Suggested by expert panel

Table S7. Overview of the tables included in the welfare assessment tool, showing the corresponding section, table name, the list of welfare indicators included that were sourced from previous studies and the list of welfare indicators included that were suggested by expert panel during the focus group meetings.

Section	Table	List indicators (physical measurements, behaviours, or interactions) sourced from previous study	List of indicators (physical measurements, behaviours or interactions) suggested during the focus group meetings
Physical health	Signs of illness	-	-Redness, swelling or loss of feathers around eyes<br>-Discharge from eyes, nares (nostrils), or mouth<br>-Crusty material in or around nares or flakiness on the skin and/or beak<br>-Overgrown beak and/or nails<br>-Upper and/or lower beak not properly aligned<br>-Vomiting or regurgitation<br>-Signs of laboured breathing, such as tail bobbing,<br>open-mouth breathing, or abnormal respiratory sounds<br>-Feather discolouration and/or depigmentation, overall dull and ragged looking plumage<br>-Stress marks: translucent or black lines in the vane of a feather, generally oriented perpendicular to the shaft<br>-Pin or blood feathers<br>-Abraded feathers<br>-Signs of self-mutilation<br>-Blood loss and/or severe injury<br>-Fluffed posture, droopy wings, and/or sleeping more than usual<br>-Enlargement and/or swelling on the body<br>->10% increase or decrease of body weight within a week<br>-Inability to perch and/or sitting at the bottom of the cage<br>-Lameness and/or shifting of body weight<br>-Uncontrolled and/or uncoordinated movement of the head, wings, and/or feet<br>-Swelling, ulcers, and/or other lesions under the feet<br>-Repeated straining, often with tail bobbing and/or fluffed posture<br>-Cloaca protrudes outside the body (prolapse)
Social and reproductive behaviours	Social behaviours	-Staying right beside another parrot<br>-Preening another parrot<br>-Being preened by another parrot<br>-Vocal interaction with another parrot (contact calls)<br>-Mating with another parrot<br>-Regurgitating food for another parrot<br>-Having food regurgitated by another parrot<br>-Attacks (biting, lunging) another parrot<br>-Being attacked (bitten or lunged at) by another parrot	-Feeding alongside another parrot<br>-Stalking (i.e. following or watching insistently) another parrot<br>-Being stalked (i.e. followed or watched insistently) by another parrot

	Reproductive behaviours	-Rubbing its cloaca (vent area) against objects such as perches, cage bars, toys<br>-Actively seeking or spending time in dark, enclosed, or secluded spaces such us underneath furniture, inside drawers or cabinets, behind cushions, or in boxes or clothing.<br>-Lunging, biting, vocalizing aggressively, or chasing anyone who approaches or gets too close to a specific area (e.g. cage or nest)	-Egg laying<br>-Regurgitating food and directing it towards its own body parts (e.g. its foot)<br>-Regurgitating food on a special toy or item in the cage
Parrot-human interactions	Behaviours allowed by humans		-Sitting on shoulder, lap, or another part of the human body excluding the hands<br>-Gently nibbling or grooming hair, beard, eyelashes, or human skin with its beak (preening)<br>-Crawling under clothes or blankets
	Types of interaction with human	-Training the parrot<br>-Providing food by mouth or allowing the parrot to eat from the mouth<br>-Holding the parrot's body in the hands (with or without gloves or a towel)<br>-Grabbing the parrot with a net or a towel	-Petting the parrot's head, cheeks and/or neck<br>-Gently responding to the parrot's vocalizations<br>-Talking to the parrot<br>-Playing music for my parrot<br>-Taking the parrot along when going out<br>- Petting the parrot under the wings<br>-Petting the parrot's chest<br>-Petting the parrot's back and tail<br>-Holding and shaking the parrot's beak while playing<br>-Yelling at the parrot<br>-Kissing the parrot<br>-Pressing on parrot's chest to encourage it to step up onto hands, arms, or an offered perch
	Human-directed behaviours	-Contact calls/vocalizations<br>-Masturbation (rubbing the cloaca against humans)<br>-Begging for food (raise its wings, flutter them and bob its head up, and down in a rhythmic pattern)<br>-Approaching a person and biting Regurgitating food<br>-Voluntarily steps up onto your hand, arm, or an offered perch without encouragement	-Offering the head/neck to be petted<br>-Crouching with the head down
Maladaptive and fear-related behaviours	Expression of avoidance or escape behaviours	-Tremors or shivering<br>-Freezing<br>-Hiding<br>-Withdrawing<br>-Attempting to escape by flying or moving away, possibly falling off the perch<br>-Screeching/high-pitched screams	
	Maladaptive, sham and excessive behaviours	-Pacing<br>-Route tracing<br>-Swaying and rocking<br>-Tongue rolling and flicking<br>-Beak rubbing<br>-Spot pecking<br>- Sham chewing and chewing not chewable items<br>-Sham bathing<br>-Toe-nail biting	-Beak clacking<br>-Repetitive licking<br>-Feeding objects (e.g. mirrors, toys)

Table S8. Welfare indicators that were among the 24 deemed most important for inclusion in the assessment tool but that were excluded after expert discussions are listed in the table below. The table also reports the reasons for their exclusion, the indicators that replaced them in the tool, and their respective sources.

Indicator excluded	Reason of exclusion	Replacing indicator	Source
Feather destructive behaviour (chewing, biting, fraying, plucking)	Experts agreed that this maladaptive behaviour is difficult to be observed directly, and its occurrence can be detected by assessing the plumage condition	Condition of body and flight feather	(Piseddu et al. 2025)
Daily food intake	Experts agreed that this measurement is impractical to be assessed by caregivers	Changes in food and water consumptions	Suggested by expert panel
Amount of time spent sleeping	Experts agreed that this measurement is impractical to be assessed by caregivers	Changes in resting/sleeping patterns	Suggested by expert panel

Table S9. Summary of the refinements made to the welfare indicators based on feedback received through online surveys from caregivers and external experts, as well as revisions made by the authors.

Section	Indicator	Refinement	Based on feedback from
General information	Rearing History	-Inclusion of definition of rearing<br>- Inclusion of the answer option “captive-bred but rearing history unknown”	Caregivers
	Frequency of health general check-ups	Answer options “Two or more times per year” and “once per year” combined in one single answer option: “once or more times per year”	Caregivers
Physical health	Number and appearance of droppings	Answer options were refined based on the frequency with which normal and abnormal droppings are observed, and accounting for diet as a factor influencing colour, odour, and consistency.	Caregivers
	Pectoral muscle condition score	-Removal of the answer option “I can't calculate the score because my parrot doesn't accept handling, and I don't feel comfortable insisting, it's too stressful and I'm afraid of hurting or scaring them.”<br>-Inclusion of the disclaimer “please calculate the score only if both you and your parrot feel comfortable while performing the assessment.”	Caregivers
	Presence of diagnosed disease	-Specified whether a potential disease had been diagnosed by an avian veterinarian.<br>-Inclusion of the footnote “could also be affected with a non-pathogen without showing any symptoms”	Experts
	Signs of illness	-Inclusion of a footnote for vomiting or regurgitation: “[1] This does not include regurgitation performed as part of courtship behaviour or chick-feeding”<br>-Separation all feather abnormalities in single rows<br>-Change “irregular” with “uncoordinated” movement<br>- Integration of a follow-up question in the table to determine whether an illness had already been diagnosed by an avian veterinarian.	Experts and caregivers
Housing and physical activity	Enclosure position	-Removal of the answer option “not applicable”<br>-Inclusion of the disclaimer “please select only the answer options that apply to your parrot and leave the remaining ones blank.”	Caregivers
	Enclosure size	-Inclusion of the answer option “my parrot has less than 2 wingspans of space but can at least extend the wings in all directions”<br>Removal of “with limited space to move” from the last answer option.	Experts
	Enclosure bars orientation	Switched the position of the vertical and horizontal bars in the welfare gradient, with horizontal bars indicating progression toward a more positive welfare gradient.	Caregivers and experts
	Enclosure material	Inclusion of the disclaimer “The choice of enclosure material should take into account the parrot’s tendency and ability to chew on the bars, since parrots with stronger beaks can break off fragments that may be ingested, creating a risk of injury or toxicity.”	Experts
	Provision of opportunities that allow movement and	Combination of “boings” with “atoms balls” with	Authors

	climbing (swings, ladders, etc.)		
	Frequency of cage, bowls, toys cleaning	Inclusion of the answer options “enclosure furnishings”, “other toys” and “Climbing materials (for example, perches or ropes)”	Experts and authors
	Access to outdoor spaces	Removal of “if environmental circumstances permit (no risk of predation or mosquitoes’ bites, appropriate temperature)” from the question	Caregivers
	Safety of outdoor spaces	Question created based on feedback received for the indicator “access to outdoor spaces”	Caregivers
	Opportunity to fly	Removal of “in a safe space” from the question	Caregivers
	Safety of the space where the parrot is allowed to fly	Question created based on feedback received for the indicator “opportunity to fly”	Caregivers
	Level of activity (time spent inactive vs active)	Reverse of the order of the answer options	Experts
Provision of enrichment and exploration	Provision of foraging and cognitive enrichment	-Change answer option “few times per month or less” to “monthly”<br>-Change “auditory enrichment (e.g. radio, music)” to “Auditory enrichment (for example, recordings of natural sounds)” and inclusion of the footnote “it should be played at a low volume and avoided when the bird is sleeping or resting”<br>-Change “visual enrichment (e .g. tv or tablets)” to “visual enrichment (for example, view out of the window)”	Caregivers, Experts and Authors
	Interaction with enrichment	-Removal of “showing continued interest until the item is destroyed (in case of destructible items) or food/contents is removed (if applicable)” from the first answer option<br>-Change answer option “shows brief interest in the enrichment, and ignores it at other times” to “occasionally interacts with the enrichment, explores and/or manipulates it briefly but ignores it at other times”<br>--Change answer option “does not use or interact with the enrichment at all, and completely ignores it” to “does not use or interact with the enrichment at all, either ignoring it completely or actively avoiding it”<br>-Change “auditory enrichment (e.g. radio, music)” to “Auditory enrichment (for example, recordings of natural sounds)” and inclusion of the footnote “it should be played at a low volume and avoided when the bird is sleeping or resting”<br>-Change “visual enrichment (e .g. tv or tablets)” to “visual enrichment (for example, view out of the window)”	Caregivers, Experts and Authors
	Time spent foraging	Reverse of the order of the answer options	Authors
	Provision of enrichment in relation to the daily food ration	-Change “more than half of” with “the majority to the entire” in the first answer option<br>-Answer options “for treats and about half of the daily food ration and “for treats and a small portion of of the daily food ration” combined in one single answer option: “For treats and for less than half of the daily food ration”<br>-Inclusion of the answer option “foraging enrichment not provided”	Authors
	Response to novel objects	The wording of the answer options was modified to improve clarity and comprehension.	Authors
	Alertness	-Removal of the second answer option because it was redundant with the first one<br>- The wording of the answer options was modified to improve clarity and comprehension.	Authors
	Composition of the diet	- The wording of the food descriptions was modified to improve clarity and comprehension.	Caregivers and authors

		-Inclusion of an icon to indicate foods appropriate for consumption in moderate to large quantities only by certain species<br>-Inclusion of the food option “fresh grass” combined with “sprouted seed”<br>-Inclusion of “dried fortified egg food” combined with “fresh egg”	
Nutrition and maintenance behaviours	Diet appropriateness	-Inclusion of “or it is advertised by the food manufacturer for my parrot species” in the second answer option	Authors
	Availability of clean fresh water	Change of the question “how often do you change your parrot's water?” to “how often do you empty and refill your parrot’s water bowl with fresh water?”	Experts
	Daytime (morning, afternoon, evening) spent sleeping/resting	Inclusion of the answer option “midday”	Experts
	Opportunity to bathe	-Inclusion of the disclaimer “please note that the frequency of bathing varies greatly depending on the species and the season.”<br>-Answer options “everyday” and “weekly” combined in the answer option “1-3 times per week”<br>-Answer option “few times per month or less” modified to “less than weekly”	Caregivers
Social and reproductive behaviours	Social housing (alone vs pair vs group)	-Removal of whether parrots are housed together from the answer options	Caregivers
	Reproductive behaviours	Answer option “few times per month or less” modified to “monthly”	Authors
Parrot-human interactions	Time spent in presence of a human	Reverse of the order of the answer options	Authors
	Behaviours allowed by humans	-Answer option “few times per month or less” modified to “monthly”<br>- “Sitting on shoulder, lap, or another part of the body” modified to “sitting on shoulder, lap, or another part of the human body excluding the hands”<br>-“Gently nibbling or grooming hair, beard, eyelashes, or skin with its beak (preening)” modified to “gently nibbling or grooming hair, beard, eyelashes, or human skin with its beak (preening)”	Experts and authors
	Types of interaction with human (training, mouth to beak feeding, etc.)	-Answer option “few times per month or less” modified to “monthly”<br>-Answer option “taking the parrot along when going out” removed<br>-Inclusion of the footnote “It should be played at a low volume and avoided when the bird is sleeping or resting” for “playing music for my parrot”<br>-Inclusion of the footnote “excluding briefly checking under the wings to assess feather condition or presence of injuries” for “petting the parrot under the wings”<br>-Inclusion of the footnote “excluding when the pectoral muscle condition score is being assessed.” for “petting the parrot’s chest”<br>-Inclusion of the footnote “excluding brief handling for physical condition assessments or medical examinations” for “holding the parrot’s body in the hands (with or without gloves or a towel)”<br>-Inclusion of the footnote “excluding brief handling for physical condition assessments or medical examinations” for “grabbing the parrot with a net or a towel”	Experts and authors

	Behaviours directed towards humans	-Answer option “rarely to never” modified to “never”<br>-Behaviour “crouching with the head down” removed<br>-Inclusion of the behaviour “mating solicitation posture (head lowered, tail raised)”	Caregivers and authors
	Response upon contact with caregiver	-Response “my parrot accepts my presence near the enclosure or stand” removed<br>-Inclusion of the response “my parrot seeks proximity to me when I am around”	Authors
	Response upon contact with familiar person	-Response “my parrot accepts their presence near the enclosure or stand” removed<br>-Inclusion of the response “my parrot seeks proximity to me when they are around”	Authors

Table S10. Table summarising the refinements made to the welfare indicators based on feedback received by expert panel following the final review of the tool.

Section	Indicator	Refinement
Housing and physical activity	Enclosure bars orientation	Inclusion of the disclaimer "please do not answer this question if your parrot has a dedicated room and/or does not have an enclosure"
	Access to outdoor spaces	-Disclaimer was modified to improve clarity and comprehension
	Safety of outdoor spaces	-Change "mosquito bites" to "biting or stinging insects" in the disclaimer
	Safety of the space where the parrot is allowed to fly	- Change "mosquito bites" to "biting or stinging insects" in the disclaimer
	Wing trimming	- Inclusion of a link to an Association of Avian Veterinarians article on the welfare implications of wing trimming.<br>- Added a description explaining the wing trim illustrations.
Provision of enrichment and exploration	Opportunities to select items based on preference (e.g., for colour, shape or type of material)	Question reworded to "Is your parrot offered a range of toys that differ in shape, size, and colour, allowing it to choose which ones to interact with?"
Social and reproductive behaviours	Social housing (alone vs pair vs group)	Answer options reworded based on the opportunity for physical interactions

References

Piseddu A, van Zeeland YRA, and Rault J-L 2025 Evaluation of welfare indicators for companion parrots: a Delphi consultation survey. *Applied Animal Behaviour Science* **283:** 106526. https://doi.org/10.1016/j.applanim.2025.106526
